# Modeling tool for calculating dietary iron bioavailability in iron-sufficient adults^1^,^2^,^3^

**DOI:** 10.3945/ajcn.116.147389

**Published:** 2017-04-05

**Authors:** Susan J Fairweather-Tait, Amy Jennings, Linda J Harvey, Rachel Berry, Janette Walton, Jack R Dainty

**Affiliations:** 4 Norwich Medical School, University of East Anglia, Norwich, United Kingdom;; 5 Institute of Food Research, Norwich Research Park, Norwich, United Kingdom; and; 6 School of Food and Nutritional Sciences, University College Cork, Cork, Ireland

**Keywords:** bioavailability model, dietary iron absorption, dietary iron intake, dietary reference values, iron, iron bioavailability, iron intake, serum ferritin

## Abstract

**Background:** Values for dietary iron bioavailability are required for setting dietary reference values. These are estimated from predictive algorithms, nonheme iron absorption from meals, and models of iron intake, serum ferritin concentration, and iron requirements.

**Objective:** We developed a new interactive tool to predict dietary iron bioavailability.

**Design:** Iron intake and serum ferritin, a quantitative marker of body iron stores, from 2 nationally representative studies of adults in the United Kingdom and Ireland and a trial in elderly people in Norfolk, United Kingdom, were used to develop a model to predict dietary iron absorption at different serum ferritin concentrations. Individuals who had raised inflammatory markers or were taking iron-containing supplements were excluded.

**Results:** Mean iron intakes were 13.6, 10.3, and 10.9 mg/d and mean serum ferritin concentrations were 140.7, 49.4, and 96.7 mg/L in men, premenopausal women, and postmenopausal women, respectively. The model predicted that at serum ferritin concentrations of 15, 30, and 60 mg/L, mean dietary iron absorption would be 22.3%, 16.3%, and 11.6%, respectively, in men; 27.2%, 17.2%, and 10.6%, respectively, in premenopausal women; and 18.4%, 12.7%, and 10.5%, respectively, in postmenopausal women.

**Conclusions:** An interactive program for calculating dietary iron absorption at any concentration of serum ferritin is presented. Differences in iron status are partly explained by age but also by diet, with meat being a key determinant. The effect of the diet is more marked at lower serum ferritin concentrations. The model can be applied to any adult population in whom representative, good-quality data on iron intake and iron status have been collected. Values for dietary iron bioavailability can be derived for any target concentration of serum ferritin, thereby giving risk managers and public health professionals a flexible and transparent basis on which to base their dietary recommendations. This trial was registered at clinicaltrials.gov as NCT01754012.

See corresponding editorial on page 1255.

## INTRODUCTION

The bioavailability of dietary iron can be defined as the proportion (or percentage) of ingested iron that is absorbed and used within the body. A value for dietary iron bioavailability (sometimes referred to as the bioavailability factor) is required to transform physiologic requirements (i.e., absorbed iron) into dietary intakes and, hence, to derive dietary reference values (DRVs)^8^ and to develop dietary recommendations and public health policies. Initially, bioavailability factors were derived from predictive algorithms on the basis of intakes of heme iron and enhancers of nonheme-iron absorption (1). This method was followed by more complex algorithms that included inhibitors as well as enhancers of nonheme-iron absorption (2, 3) whereby the magnitude of the effect of modifiers of nonheme-iron absorption was determined from single-meal studies. Because the effect of enhancers and inhibitors may be exaggerated in single-meal studies (4), the mean absorption of nonheme iron from >1 meal was used to more closely reflect the whole diet (5, 6). However, this assessment does not reflect the diet that is consumed over time, and also, an adjustment has to be made to take into account the heme content of the diet with an assumed absorption value.

We recently developed a novel predictive model, to our knowledge, for the estimation of dietary iron bioavailability on the basis of measurements of total iron intake (heme and nonheme iron), the serum ferritin (SF) concentration, and factorial calculations of iron requirements (7). The latter measurements were derived with the use of the National Academy of Medicine approach for the estimation of iron losses (8). Individual data of 495 men and 378 premenopausal women were used for a model that estimated the prevalence of dietary intakes that were assumed to be insufficient to meet the needs of men and women (separately) on the basis of their daily iron intakes and a series of absorption values. The prevalence of SF concentrations less than selected cutoffs was derived, and an estimate of dietary iron absorption that was required to maintain specific SF values was calculated by matching the observed prevalence of insufficiency with the prevalence that was predicted for the series of absorption estimates. Therefore, it was possible to estimate dietary iron absorption (bioavailability) at a population level from the individual measurements of total iron intake and the SF concentration. In this article, we describe the results of the application of the model to other studies and present a refined interactive model that can be used as a tool to predict dietary iron bioavailability in populations in whom iron intakes and SF concentrations have been measured.

## METHODS

Data were used from the following 3 studies: the National Diet and Nutrition Survey (NDNS), the National Adult Nutrition Survey (NANS), and the New Dietary Strategies Addressing the Specific Needs of Elderly Population for a Healthy Ageing in Europe (NU-AGE). Briefly, the NDNS (9) and NANS (10) used nationally representative samples of adults (with the exclusion of pregnant women and breastfeeding women) in the United Kingdom (aged 19–64 y) and Republic of Ireland (aged ≥19 y), respectively. The NU-AGE study was a randomized, controlled, multicenter trial of healthy, independent older people (without frailty, heart failure, or serious chronic illness) aged 65–79 y with the aim of assessing the effects of a 1-y dietary intervention on markers of inflammation and health (11, 12). We used baseline data from United Kingdom participants only because their dietary patterns were likely to be similar to those in the other UK surveys; the data were collected between September 2012 and January 2014. Detailed methods of the data collection have been previously published (9–12), but the information that is pertinent to this article (dietary assessment and analytic methods) is summarized as follows.

Dietary intake was assessed with the use of 7-d food diaries in the NDNS and NU-AGE with the use of 4-d semiweighed food records in the NANS. Participants were asked to record detailed information on the amounts and types of all foods and drinks that were consumed over consecutive days. To ensure the accuracy of recordings, participants were interviewed or a researcher visited participants in their homes to review the food records and clarify any inconsistencies.

Height was measured to the nearest 0.1 cm with the use of the a Leicester Height Measure in all 3 studies, and weight was measured to the nearest 100 g with the use of calibrated scales [in the NDNS: Quantratronic scales (Soehnle); in the NANS: Body Composition Analyzer BC-420MA (Tanita); and in the NU-AGE study: Electronic Column Scales (Seca)].

Blood samples reached laboratories within 5 h of collection and were processed and stored at −80°C until required for further analysis. SF was measured either with the use of a microparticle enzyme immunoassay assay (IMxl; Abbott Laboratories) in the NDNS, an automated analyzer (RX Daytona; Randox) in the NANS, or an electrochemiluminescence immunoassay (cobas 6000; Roche Diagnostics) in the NU-AGE study. Hemoglobin concentrations were determined with the use of a Bayer H3 automated analyzer (Bayer-Diagnostik) in the NDNS, a Coulter LH700 series analyzer (Beckman Coulter Diagnostics Ltd, Co.) in the NANS, or Sysmex XN analyzer (Sysmex America Inc.) in the NU-AGE study.

SF is an acute-phase reactant, and therefore, in the presence of infection or inflammation, the concentration does not accurately reflect iron stores. C-reactive protein (CRP) and α-1-antichymotrypsin are 2 of the biomarkers that are used to detect the presence of infection or inflammation and, hence, enable the exclusion of individuals with artificially high SF values (13). Serum CRP [high-sensitivity CRP (hs-CRP)] concentrations were measured with the use of an automated analyzer (RX Daytona; Randox) in the NANS and ProcartaPlex kits (Affimetrix) in the NU-AGE study, and any participant with a raised hs-CRP concentration (>5 mg/L) was excluded. In the NDNS, the acute-phase reactant α-1-antichymotrypsin was measured.

In the NANS, 1500 individuals were recruited to the study, and hs-CRP was measured in 849 subjects. Subjects with a CRP concentration <5 mg/L (*n* = 719), who were not taking supplements that contained iron (*n* = 656), and in whom SF had been measured (*n* = 650) were included in the analysis. In the United Kingdom arm of the NU-AGE study, 272 participants were recruited. Complete data on all relevant variables were available for 246 participants, but 13 participants (5%) with raised hs-CRP concentrations (>5 mg/L) were subsequently excluded, and 37 participants were excluded because they were taking supplements that contained iron; these exclusions left 196 subjects whose data were included in the current analysis. In the NDNS data, we used the same exclusion criteria as in the other 2 studies (i.e., subjects were excluded if they were taking supplements that contained iron or had raised inflammatory markers), as described previously (7).

Iron absorption was estimated from measured iron intakes along a scale of assumed iron-absorption values (1–40%). Requirements for absorbed iron were predicted with the use of the Institute of Medicine’s distribution of dietary intake requirements with values interpolated to derive iron-absorption requirements for each 0.5th percentile (9). These values were compared with each individual’s absorbed iron estimate at each point on the 1–40% scale, and the average absorption for the population was calculated. The subtraction of these values from 100 gave the estimated percentage of the population who required higher iron absorption to meet their requirements (i.e., the estimated prevalence of inadequate iron intakes). A model was created for the prediction of dietary iron absorption at each SF concentration with the use of the assumption that the estimated prevalence of inadequate intakes would be equivalent to the observed prevalence of iron insufficiency as defined by the SF concentrations.

### Ethics

Ethical approval was granted by The South Thames Multicentre Research Ethics Committee (https://www.gov.uk/government/uploads/system/uploads/attachment_data/file/216484/dh_128550.pdf) for the NDNS, by University College Cork Clinical Research Ethics Committee of the Cork Teaching Hospitals [ECM 3 (p) 04/11/08] for the NANS, and by the National Research Ethics Committee East of England (12/EE/0109) for the NU-AGE study. Written informed consent was obtained from all participants.

### Statistics

In view of the effects of sex and menstrual blood loss in women on iron status, all analyses were stratified by sex and menopausal status. Differences in the characteristics of the participants in the 3 study cohorts were compared with the use of a 1-factor ANOVA. The distribution of SF was calculated individually for each sex and menopausal group, and the cumulative frequencies were calculated.

We examined associations between estimated iron intake from meat and SF concentrations. Quintiles of intake were calculated, and an ANCOVA was used to calculate adjusted means and to evaluate statistical trends with adjustments for age, BMI, total iron intake, and study cohort. The statistical analysis was performed with the use of Stata version 14 software (StataCorp LP) and R version 3.2.3 software (R Foundation for Statistical Computing) (14).

## RESULTS

A flowchart of the numbers of participants who were recruited and excluded at different stages of the 3 studies is available is shown in **Supplemental Figure 1**. Details of the 3 studies, including study subjects, exclusion criteria, analytic methods, and dietary assessment are summarized in **Supplemental Table 1**. The characteristics of participants from the 3 studies are presented in **Table 1**, and individual data are given in **Supplemental File 1**. The percentages of individuals with acute-phase reactant values that were indicative of inflammation or infection (hs-CRP concentration >5 mg/L or α-1-antichymotrypsin concentration >0.65 g/L) were 0% in the NDNS, 15% in the NANS, and 5% in NU-AGE study. These individuals were excluded from the analysis because their SF concentrations may have been elevated and, therefore, might not have reflected iron stores accurately.

**TABLE 1:** Characteristics, iron status, and dietary intake of participants stratified by study, sex, and menopausal status^1^

	Men				Premenopausal women			Postmenopausal women			
	NANS	NDNS	NU-AGE	*P*	NANS	NDNS	*P*	NANS	NDNS	NU-AGE	*P*
*n*	336	494	77		197	363		117	158	119	
Age, y	42.8 ± 16.4	42.4 ± 12.0	70.2 ± 3.8	<0.001	34.8 ± 9.4	34.9 ± 7.4	0.80	62.4 ± 8.9	57.5 ± 4.1	69.8 ± 3.9	<0.001
Weight,^2^ kg	87.1 ± 13.6	83.7 ± 14.1	82.4 ± 12.2	0.001	69.7 ± 12.1	67.8 ± 14.3	0.13	72.0 ± 12.7	71.2 ± 13.2	68.7 ± 10.9	0.09
BMI,^2^ kg/m^2^	28.0 ± 4.0	27.1 ± 4.3	27.0 ± 3.7	0.01	25.9 ± 4.4	25.9 ± 5.5	0.97	28.0 ± 4.7	27.7 ± 5.1	26.4 ± 3.7	0.02
Hemoglobin,^3^ g/dL	15.2 ± 1.1	15.1 ± 1.1	14.7 ± 0.9	<0.001	13.3 ± 1.1	13.4 ± 1.0	0.69	13.4 ± 1.0	13.5 ± 1.1	12.9 ± 3.6	0.07
Serum ferritin, μg/L	172 ± 135	119 ± 92.5	146 ± 102	<0.001	57.9 ± 57.8	44.7 ± 37.0	0.001	116 ± 90.8	77.0 ± 55.3	104 ± 67.6	<0.001
Iron, mg/d	13.8 ± 5.7	13.4 ± 5.1	14.3 ± 3.4	0.37	11.1 ± 4.6	9.8 ± 3.8	0.001	10.2 ± 3.3	10.9 ± 3.8	11.6 ± 3.1	0.01
Iron from meat, mg/d	2.8 ± 1.7	2.6 ± 1.6	1.3 ± 0.8	<0.001	1.8 ± 1.4	1.5 ± 1.1	0.002	1.7 ± 1.2	1.5 ± 1.1	1.0 ± 0.7	<0.001

1 Values are means ± SDs. *n* = 1861. NANS, National Adult Nutrition Survey; NDNS, National Diet and Nutrition Survey; NU-AGE, New Dietary Strategies Addressing the Specific Needs of Elderly Population for a Healthy Ageing in Europe.

2 Missing data for *n* = 21.

3 Missing data for *n* = 20.

The combined mean ± SD iron intakes were 13.6 ± 5.2, 10.3 ± 4.1, and 10.9 ± 3.5 mg/d in men, premenopausal women, and postmenopausal women, respectively. For postmenopausal women, mean intake was very close to the population reference intake (PRI) of 11 mg/d, and for men, it was higher than the PRI of 11 mg/d, but for premenopausal women, intake was lower than the PRI of 16 mg/d (15). However, all groups had intakes above the average requirement (6, 7, and 6 mg/d for men, premenopausal women, and postmenopausal women, respectively).

**FIGURE 1:**
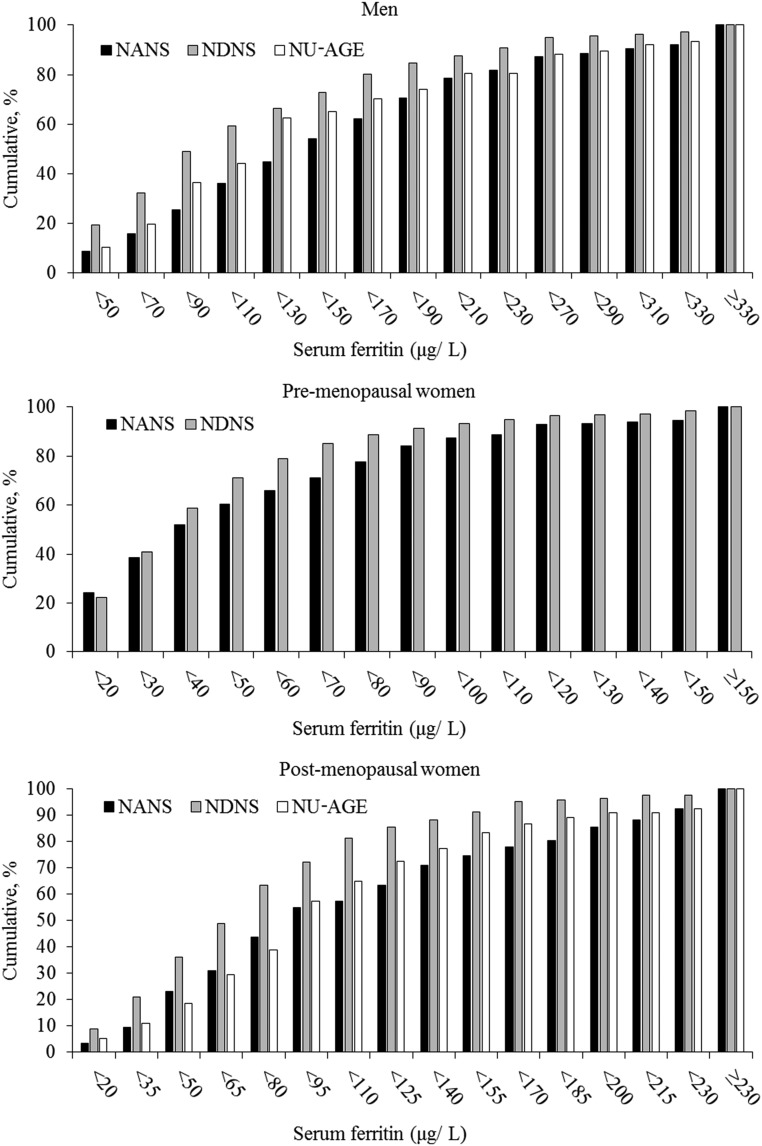
Cumulative distribution (percentage of participants in each group) of serum ferritin concentrations for men, premenopausal women, and postmenopausal women by study. The number of participants were as follows—men: black bars, *n* = 336; gray bars, *n* = 494; and white bars, *n* = 77; premenopausal women: black bars, *n* = 197; and gray bars, *n* = 363; and postmenopausal women: black bars, *n* = 117; gray bars, *n* = 158; and white bars, *n* = 119. Mean ± SD serum ferritin values were 140.7 ± 113.6, 49.4 ± 45.8, and 96.7 ± 72.8 μg/L in men, premenopausal women, and postmenopausal women, respectively. NANS, National Adult Nutrition Survey; NDNS, National Diet and Nutrition Survey; NU-AGE, New Dietary Strategies Addressing the Specific Needs of Elderly Population for a Healthy Ageing in Europe.

The majority of participants (95%) were iron sufficient (SF concentration >15 μg/L). Mean ± SD SF values were 140.7 ± 113.6, 49.4 ± 45.8, and 96.7 ± 72.8 μg/L in men, premenopausal women, and postmenopausal women, respectively; the cumulative distributions of SF concentrations are shown in **Figure 1**. There was a significant difference in mean SF concentrations between the 3 cohorts with higher values reported in the NANS across all sex- and menopausal-status groups (*P* < 0.001 in men and postmenopausal women; *P* = 0.001 in premenopausal women). Despite higher SF concentrations, iron intake was not higher in the NANS than in the other 2 cohorts, although iron intake from meat was significantly higher (*P* < 0.001 in men and postmenopausal women; *P* = 0.002 in premenopausal women).

**FIGURE 2:**
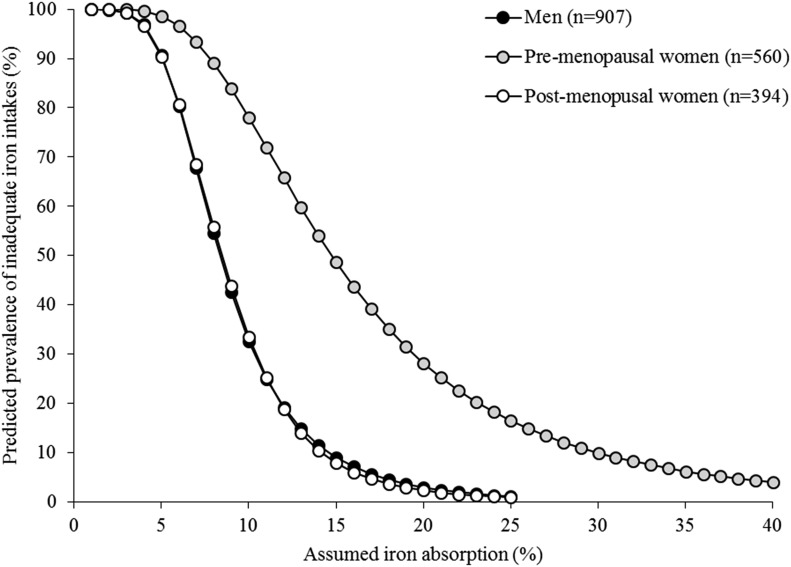
Predicted prevalence (percentage) of inadequate iron intakes at different iron absorptions in men, premenopausal women, and postmenopausal women. Dietary absorption values ranged from 0% to 40%.

**FIGURE 3:**
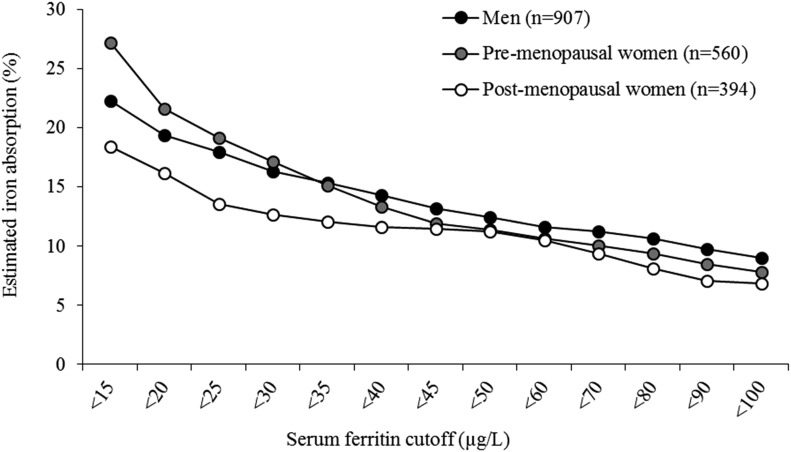
Percentage of estimated dietary iron absorption for selected serum ferritin values for men, premenopausal women, and postmenopausal women. Serum ferritin concentrations ranged from <15 to 100 μg/L.

**Figure 2** shows the predicted prevalence of inadequate iron intakes at different estimated iron absorptions with the use of combined data from the 3 cohorts. When iron absorption was 18%, the predicted prevalence of inadequate iron intakes were 5%, 35%, and 3% in men, premenopausal women, and postmenopausal women, respectively. These data reflect the capacity of the diet to meet iron requirements and, when combined with SF values, allow for the prediction of the dietary absorption that is required to maintain a specific iron status (**Supplemental File 2**). For example, at SF concentrations <15 μg/L, the mean dietary iron absorption ranges from 19% in postmenopausal women to 27% in premenopausal women compared with 11–12% for SF concentrations of 60 μg/L (**Figure 3**).

**FIGURE 4:**
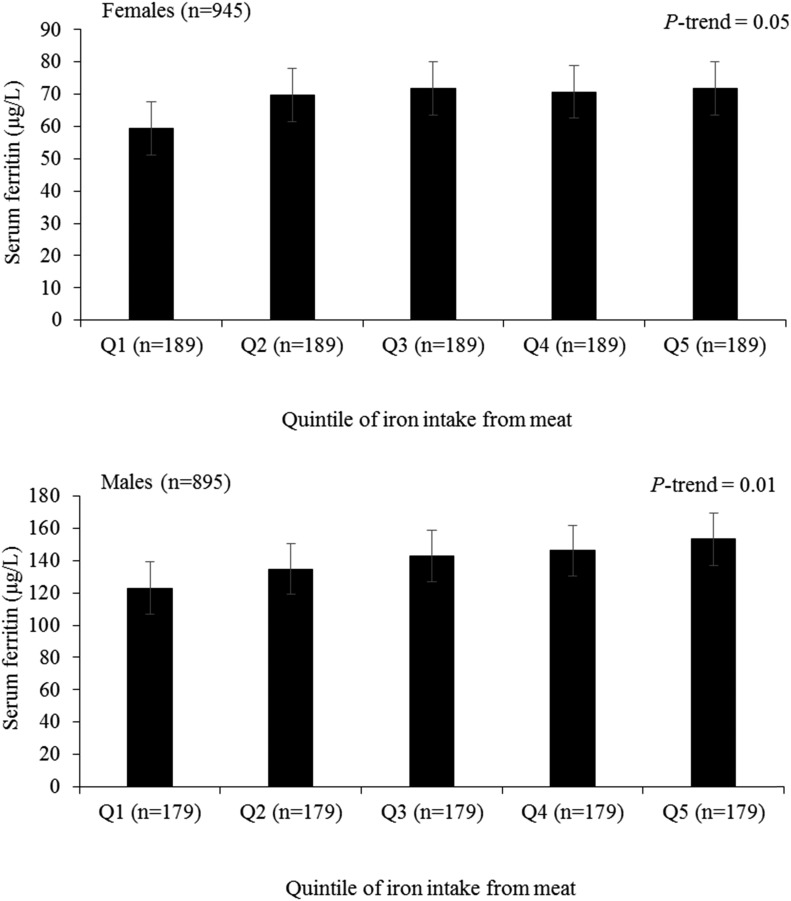
Adjusted mean ± SE serum ferritin concentrations by Q of iron intake from meat stratified by sex and adjusted for age (years), BMI (in kg/m^2^), total iron intake (milligrams per day), and study cohort. Mean ± SD iron intakes from meat in each Q were as follows; women: 1, 0.2 ± 0.2 mg/d; 2, 0.8 ± 0.1 mg/d; 3, 1.3 ± 0.1 mg/d; 4, 1.9 ± 0.2 mg/d; and 5, 3.2 ± 1.2 mg/d; men, 1, 0.6 ± 0.3 mg/d; 2, 1.5 ± 0.2 mg/d; 3, 2.3 ± 0.2 mg/d; 4, 3.1 ± 0.3 mg/d; and 5, 5.0 ± 1.5 mg/d. *P*-trend values were calculated with the use of an ANCOVA. Q, quintile.

In both men and women, there was a positive association between iron intake from meat and SF after adjustments for total iron intake, age, and BMI (**Figure 4**). There was a difference in iron intake from meat between extreme quintiles of intake of 4.3 mg for men and 3.0 mg for women. The mean ± SD SF concentration was 32.0 ± 11.8 μg/L higher in quintile 5 than in quintile 1 of intake for men (*P*-trend = 0.02) and 14.9 ± 6.1 μg/L higher for women (*P*-trend = 0.01). The program for calculating dietary iron absorption at any SF concentration is shown in Supplemental File 2.

## DISCUSSION

In our model, differences in iron status between the 3 study population groups were partly explained by age (compared with premenopausal women, postmenopausal women had lower iron status because of their lower iron requirements) and also by diet (i.e., higher intake of meat in the NANS groups was associated with higher SF concentrations). When adequate body iron stores were present at an SF concentration of 60 μg/L, the efficiency of iron absorption was no longer upregulated (16), and the computed differences in dietary iron absorption were minimal, but with a lower SF concentration, the effect of the diet became more marked, thereby illustrating the importance of applying iron intake and SF data that are collected in populations with different dietary patterns. In particular, it appears that meat consumption is a key determinant of body iron status.

Although iron requirements for individuals can be estimated reasonably accurately (15), dietary intake needed to supply this quantity of absorbed iron is notoriously difficult to estimate because of the uncertainty about dietary iron absorption. In healthy individuals, key determinants of fractional iron absorption are dietary factors (17) and body iron status (18) plus the short-term regulation that is related to previous exposure of mucosal cells to iron (19). However, when reliable measures of total iron intake and body iron status exist, the unknown variable (dietary iron absorption) can be computed by taking into account calculated physiologic requirements. A strength of our study is the use of high-quality data of iron intake and iron status. Furthermore, individuals with raised inflammatory markers were removed from the data set that was used to derive the model because they may have had an artificially high SF concentration that did not accurately reflect body iron stores. We also excluded individuals who had been taking supplements that contained iron because it was impossible to quantify the contribution of the supplements to total iron intake.

There are some limitations that should be considered when the model is used. Although the 3 data sets that were used for this study were obtained from four 7-d dietary intakes (Supplemental Table 1), the participant burden should be considered, particularly for large-scale epidemiologic studies or surveys. Data that are collected with the use of other dietary assessment methods, such as a 24-h recall or food-frequency questionnaire, may still be applied to the model, but the limitations of these intake methods should be acknowledged in the conclusions. Although we were able to exclude users of supplements that contained iron and also individuals with elevated inflammatory markers from the data sets, there was insufficient information available to assess whether any individuals were taking prescribed or over-the-counter medicines or had particular medical conditions that could have affected iron absorption or body iron status. Individuals with chronic conditions were generally excluded from participation in the studies, although the aim was to select a cohort that was representative of the population group. Furthermore, evidence for the effect of specific medical conditions and medicines on iron absorption or status has been limited, and a large proportion of the general population routinely take some form of medication; therefore, the exclusion of these individuals is not practical and would result in a very limited data set. However, it remains important to consider all of these potential issues when collecting data for the model and interpreting the results.

Although it is not possible to measure iron requirements accurately in large numbers of individuals, particularly in women of child-bearing age whose requirements are largely dictated by the magnitude of menstrual blood loss, population means can be computed, and these means are needed to set DRVs and to develop dietary guidelines and public health policies.

When setting DRVs for adults, the National Academy of Medicine (2001) used an iron bioavailability value of 18%. This value was computed by assuming 10% of dietary iron was heme iron with an absorption of 25% and that the absorption of the remaining 90% of iron (nonheme) was 16.8% in individuals with an SF concentration of 15 μg/L (4). The WHO/FAO took variations in the properties of the diet into account when proposing bioavailability figures and set DRVs on the basis of 4 different values as follows: 15% and 12% for Western-type diets, depending mainly on the amount of meat intake, and 10% and 5% for developing countries (20). In Europe, the European Food Safety Authority (15) applied the probability model that was developed by Dainty et al. (7) to derive values of 16% for men and 18% for premenopausal women with a population mean SF concentration of 30 μg/L. The UK Committee on Medical Aspects of Food Policy (21) selected 15% absorption as being typical in industrialized countries, and the Nordic countries (22) also applied an iron-absorption value of 15% when setting DRVs.

In conclusion, the lack of consensus in values for dietary iron absorption reflect in part differences in the type of diet that is considered representative for the adult population in the country (or group of countries) under consideration but also illustrates differences in the selection and interpretation of evidence on which to base the value. We have further evaluated the model developed by Dainty et al. (7) with the use of survey data from populations consuming Western diets, and the interactive model is provided in Supplemental File 2. The use of this model would facilitate harmonization in deriving values for dietary iron absorption and thereby reduce uncertainty. The model can be applied to any adult population in whom representative, good-quality data on iron intake and iron status have been collected. Furthermore, dietary iron–bioavailability values can be derived for any target concentration of SF, thereby giving risk managers and public health professionals a flexible and transparent basis on which to base their dietary recommendations.

## Supplementary Material

Online Supporting Material

## Footnotes

8 Abbreviations used: CRP, C-reactive protein; DRV, dietary reference value; hs-CRP, high-sensitivity C-reactive protein; NANS, National Adult Nutrition Survey; NDNS, National Diet and Nutrition Survey; NU-AGE, New Dietary Strategies Addressing the Specific Needs of Elderly Population for a Healthy Ageing in Europe; PRI, population reference intake; SF, serum ferritin.

## References

[1] Monsen ER, Hallberg L, Layrisse M, Hegsted DM, Cook JD, Mertz W, Finch CA Estimation of available dietary iron. Am J Clin Nutr 1978;31:134–41. 619599 10.1093/ajcn/31.1.134

[2] Hallberg L, Hulthen L Prediction of dietary iron absorption: an algorithm for calculating absorption and bioavailability of dietary iron. Am J Clin Nutr 2000;71:1147–60. 10799377 10.1093/ajcn/71.5.1147

[3] Reddy MB, Hurrell RF, Cook JD Estimation of nonheme-iron bioavailability from meal composition. Am J Clin Nutr 2000;71:937–43. 10731500 10.1093/ajcn/71.4.937

[4] Cook JD, Dassenko SA, Lynch SR Assessment of the role of nonheme-iron availability in iron balance. Am J Clin Nutr 1991;54:717–22. 1897479 10.1093/ajcn/54.4.717

[5] Armah SM, Carriquiry A, Sullivan D, Cook JD, Reddy MB A complete diet-based algorithm for predicting nonheme iron absorption in adults. J Nutr 2013;143:1136–40. 23700342 10.3945/jn.112.169904

[6] Collings R, Harvey LJ, Hooper L, Hurst R, Brown TJ, Ansett J, King M, Fairweather-Tait SJ The absorption of iron from whole diets: a systematic review. Am J Clin Nutr 2013;98:65–81. 23719560 10.3945/ajcn.112.050609

[7] Dainty JR, Berry R, Lynch SR, Harvey LJ, Fairweather-Tait SJ Estimation of dietary iron bioavailability from food iron intake and iron status. PLoS One 2014;9:e111824. 25356629 10.1371/journal.pone.0111824 PMC4214798

[8] Food and Nutrition Board, Institute of Medicine. Dietary Reference Intakes for vitamin A, vitamin K, arsenic, boron, chromium, copper, iodine, iron, manganese, molybdenum, nickel, silicon, vanadium and zinc. Washington (DC): National Academy Press; 2001. 25057538

[9] Hoare JHL, Bates CJ, Prentice A, Birch M, Swan G, Farron M National Diet and Nutrition Survey: adults aged 19 to 64 years. Vol. 5: summary report. London: TSO; 2004.

[10] Irish Universities Nutrition Alliance (IUNA). National Adult Nutrition Survey. 2011 [cited 2016 Sep 1]. Available from: http://www.iuna.net.

[11] Berendsen A, Santoro A, Pini E, Cevenini E, Ostan R, Pietruszka B, Rolf K, Cano N, Caille A, Lyon-Belgy N, Reprint of: a parallel randomized trial on the effect of a healthful diet on inflammageing and its consequences in European elderly people: design of the NU-AGE dietary intervention study. Mech Ageing Dev 2014;136–7:14–21. 10.1016/j.mad.2014.03.001 24657127

[12] Santoro A, Pini E, Scurti M, Palmas G, Berendsen A, Brzozowska A, Pietruszka B, Szczecinska A, Cano N, Meunier N, Combating inflammaging through a Mediterranean whole diet approach: the NU-AGE project’s conceptual framework and design. Mech Ageing Dev 2014;136–137:3–13. 10.1016/j.mad.2013.12.001 24342354

[13] WHO. C-reactive protein concentrations as a marker of inflammation or infection for interpreting biomarkers of micronutrient status. Vitamin and Mineral Nutrition Information System. (WHO/NMH/NHD/EPG/14.7.) Geneva (Switzerland): WHO; 2014 [cited 2016 Dec 5]. Available from: http://apps.who.int/iris/bitstream/10665/133708/1/WHO NMH NHD EPG 14.7 eng.pdf?ua=1.

[14] R Development Core Team. 2015 R: A language and environment for statistical computing [Internet]. Vienna (Austria): R Foundation for Statistical Computing [cited 2015 May 23]. Available from: https://www.R-project.org/.

[15] EFSA NDA Panel (EFSA Panel on Dietetic Products, Nutrition and Allergies). Scientific opinion on dietary reference values for iron. EFSA J 2015;13:4254.

[16] Hallberg L, Hultén L, Gramatkovski E Iron absorption from the whole diet in men: how effective is the regulation of iron absorption? Am J Clin Nutr 1997;66:347–56. 9250114 10.1093/ajcn/66.2.347

[17] Hurrell R, Egli I Iron bioavailability and dietary reference values. Am J Clin Nutr 2010;91:1461S–7S. 20200263 10.3945/ajcn.2010.28674F

[18] Ganz T Systemic iron homeostasis. Physiol Rev 2013;93:1721–41. 24137020 10.1152/physrev.00008.2013

[19] Viteri FE, Xunian L, Tolomei K, Martin A True absorption and retention of supplemental iron is more efficient when iron is administered every three days rather than daily to iron-normal and iron-deficient rats. J Nutr 1995;125:82–91. 7815180 10.1093/jn/125.1.82

[20] WHO/FAO. Vitamin and mineral requirements in human nutrition: report of a Joint FAO/WHO expert consultation. Geneva (Switzerland): WHO; 2004.

[21] Department of Health. Dietary reference values for food energy and nutrients for the United Kingdom. Report of the Panel On Dietary Reference Values of the Committee on Medical Aspects of Food Policy. London: HMSO; 1991. 1961974

[22] Nordic Nutrition Recommendations. Integrating nutrition and physical activity. Copenhagen (Denmark): Narayana Press; 2012.

