# Supplementary material for: Modeling tool for calculating dietary iron bioavailability in iron-sufficient adults
Source: Am J Clin Nutr. 2017 Apr 5;105(6):1408–14. doi: 10.3945/ajcn.116.147389 (PMC5533300; doi:10.3945/ajcn.116.147389)
Supplement: Online Supporting Material [file supp_105_6_1408__index.html]

Modeling tool for calculating dietary iron bioavailability in iron-sufficient adults — Online Supporting Material 

# Modeling tool for calculating dietary iron bioavailability in iron-sufficient adults

## Online Supporting Material

- Online Supporting Material - Figure 1
- Online Supporting Material - Table 1
- Online Supporting Material - File 1
- Online Supporting Material - File 2
