# Supplementary material for: Modeling tool for calculating dietary iron bioavailability in iron-sufficient adults
Source: Am J Clin Nutr. 2017 Apr 5;105(6):1408–14. doi: 10.3945/ajcn.116.147389 (PMC5533300; doi:10.3945/ajcn.116.147389)

**Supplemental Figure 1: Participant flow chart**

Numbers of participants recruited and excluded at various stages (missing data, raised CRP or ACT) in NANS (National Adult Nutrition Survey), NDNS (National Diet and Nutrition Survey), and NU-AGE (New dietary strategies addressing the specific needs of the elderly population for healthy aging in Europe), and the number of participants included in the final data analysis.

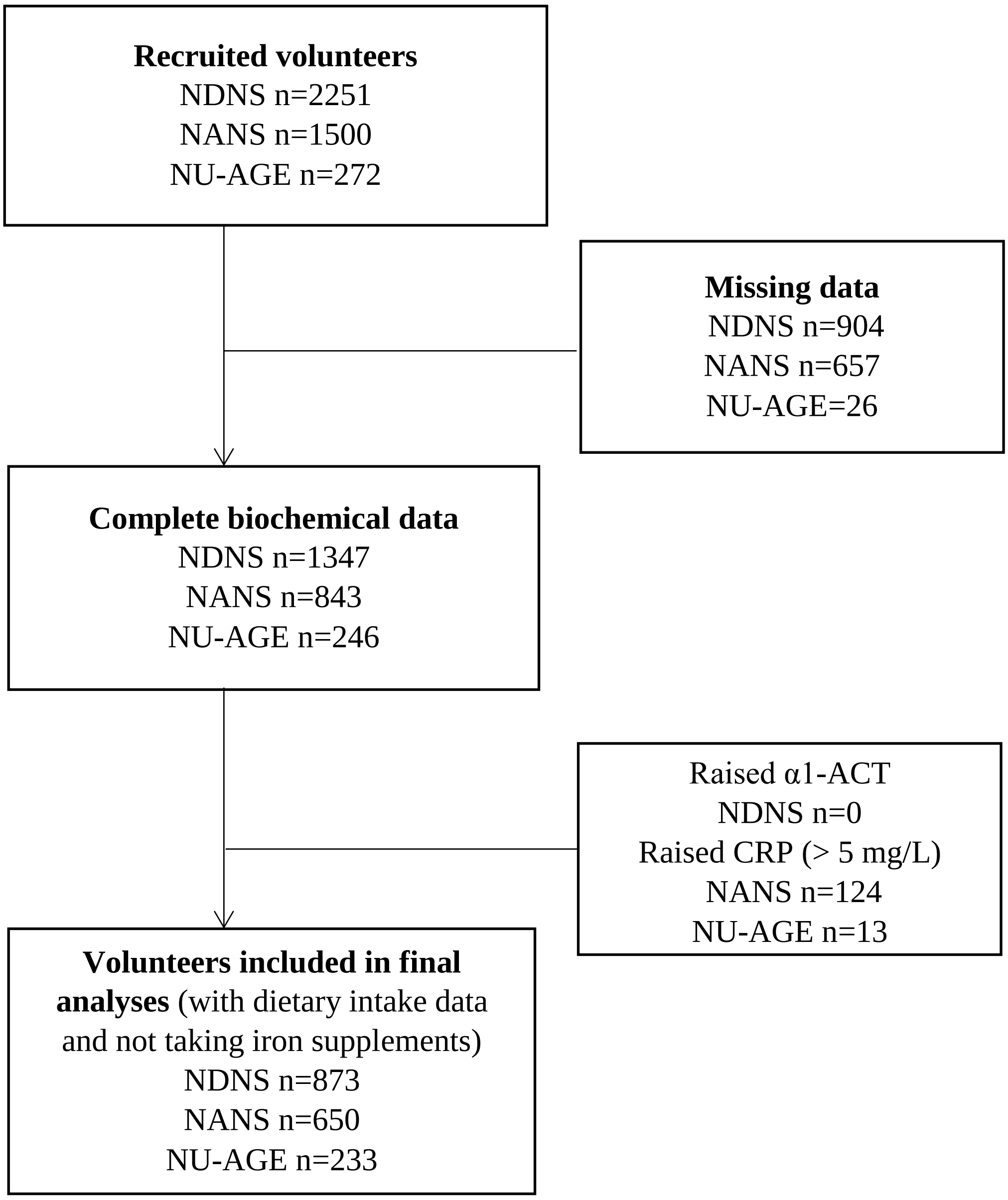

Supplement: Online Supporting Material [file 116.147389_ajcn147389SupplementaryData1.pdf]
