# Supplementary material for: Modeling tool for calculating dietary iron bioavailability in iron-sufficient adults
Source: Am J Clin Nutr. 2017 Apr 5;105(6):1408–14. doi: 10.3945/ajcn.116.147389 (PMC5533300; doi:10.3945/ajcn.116.147389)
Supplement: Online Supporting Material [file 116.147389_ajcn147389SupplementaryData2.pdf]

Supplemental Table 1. Description of the main methods and characteristics for each of the included studies
NDNS, National Diet and Nutrition Survey; NANS, National Adult Nutrition Survey; NU-AGE, New Dietary Strategies Addressing the Specific Needs of the Elderly Population for Healthy Ageing in Europe study

	**NDNS**	**NANS**	**NU-AGE**
**General**			
Full study title	National Diet and Nutrition Survey: adults aged 19 to 64 years	National Adult Nutrition Survey	New Dietary Strategies Addressing the Specific Needs of the Elderly Population for Healthy Ageing in Europe study
Country	Great Britain	Republic of Ireland	Great Britain
Responsible Institution(s)	NatCen Social Research and MRC Human Nutrition Research	Irish Universities Nutrition Alliance (IUNA)	University of East Anglia
Funding	Food Standards Agency, the Ministry of Agriculture, Fisheries and Food, the Department of Health	Department of Agriculture, Food & the Marine as part of the Food Institutional Research Measure under the Food for Health Research Initiative (FHRI).	European Union Seventh Framework Programme
Year(s)	2000-2001	2008-2010	2012-2014
Ethical approval	South Thames Multi-Centre Research Ethics Committee (2000) and subsequent approval from 93 NHS Local Research Ethics Committees	Clinical Research Ethics Committee of the Cork Teaching Hospitals, University College Cork and the Human Ethics Research Committee of University College Dublin	National Research Ethics Committee East of England
**Study design & participants**			
Study design	Cross-sectional survey	Cross-sectional survey	Cross sectional survey of baseline randomised controlled trial data.

Study duration or length of follow-up (if applicable)	N/A	N/A	1 year
Total number of participants recruited	2251 participants<br>1347 provided a blood sample and food diary	1500 participants<br>1138 provided a blood sample and food diary	272
Inclusion criteria (participant characteristics)	Living in private households, aged between 19 and 64 years. Where more than one eligible person was living in an address, one was randomly selected to participate.	Free-living adults aged 18 years or over	Independently living adults aged 65-79 y.
Exclusion criteria	Pregnant or breastfeeding.	Pregnant or breastfeeding	Clinically diagnosed chronic disease, use of corticosteroids or insulin medications, recent use of antibiotics or vaccinations, recent change in habitual medication, presence of food allergy or intolerance necessitating a special diet, presence of frailty or malnutrition.
Recruitment method	Multi-stage random probability design using the Postcode Address File. 152 postal sectors were selected, with 40 postal addresses randomly selected from each	Quota sampling following the approach used in the national census, utilising the names and addresses in the National Postal Service database.	Local advertisements, publicity, and general practitioner surgeries
**Dietary assessment**			
Dietary assessment method(s)	Weighed food intake record and initial dietary interview	Semi-weighed food diary	Estimated food intake record and initial dietary interview.

Number of days/recalls	7 day record	4 consecutive days including 1 week-end day	7 days
**Biomarkers**			
Samples collected	24hr urine collection, blood sample	Urine sample, blood sample	24hr urine collection, blood sample
Iron biomarkers and methods	**Serum ferritin**: Abbott IMx semi-automated analyser using a standard Microparticle Enzyme Immunoassay (MEIA) kit.<br>**Hemoglobin**: Bayer H3 Haematology Analyser using a colorimeter at 546nm.	**Serum ferritin:** Automated analyser (RX Daytona, Randox).<br>**Hemoglobin:** Coulter LH700 series analyser.	**Serum ferritin**: Electrochemiluminescence immunoassay (Cobas 6000, Roche Diagnostics)<br>**Hemoglobin**: Sysmex XN analyser
Markers of inflammation and methods	**α-1-antichymotrypsin**: Hitachi 912-based nephelometric assay	hs-CRP: Automated analyser (RX Daytona, Randox).	**CRP:** ProcartaPlex kits (Affimetrix)
**Additional information**			
Key references and web-pages	All reports and appendices can be found at: http://tna.europarchive.org/20110116113217/http://www.food.gov.uk/science/dietarysurveys/ndnsdocuments/ndnspreviousssurveyreports	Reports and appendices can be found at: http://www.iuna.net/?p=106	
