# Supplementary material for: Modeling tool for calculating dietary iron bioavailability in iron-sufficient adults
Source: Am J Clin Nutr. 2017 Apr 5;105(6):1408–14. doi: 10.3945/ajcn.116.147389 (PMC5533300; doi:10.3945/ajcn.116.147389)
Supplement: Online Supporting Material [file 116.147389_ajcn147389SupplementaryData3.pdf]

CODE	GENDER	AGE	WEIGHT	BMI	HAEMOGL(	SERUM FEI	IRON	IRON_MEA	CALCIUM	SAMPLE
105	Male	54	83.7	23.09	15.5	132.12	43.15	0.56	954.16	NANS
114	Female	76	73.6	28.22	14.3	134.51	5.02	0.43	737.02	NANS
121	Male	58	92.1	30.7	14.2	242.38	13.67	1.13	1186.76	NANS
122	Male	33	72.7	23.47	13.9	165.85	18.24	3.07	803.51	NANS
123	Female	47	66	22.84	13.4	78.93	14.76	7.1	860.69	NANS
134	Female	72	54	22.59	12.1	49.21	4.92	0.71	1852.17	NANS
135	Female	73	87.6	30.38	14.2	342.58	11.73	1.96	797.17	NANS
139	Male	80	70.9	29.43	15.1	242.58	11.76	3.61	840.54	NANS
143	Female	49	75.1	23.94	13.5	119.18	22.96	3.76	1448.96	NANS
147	Male	62	99.6	30.1	15.1	117.67	19.17	2.86	1390.24	NANS
149	Male	49	72.9	29.54	12.1	14.8	12.48	0.74	1018.97	NANS
153	Male	35	72.6	25.51	14.7	195.21	13	0.71	828.53	NANS
156	Male	41	103.2	32.64	14.2	108.3	14.96	0.18	2107.32	NANS
170	Male	51	89.15	32.51	14.6	170.62	15.31	4.46	831.34	NANS
171	Female	76	55	22.06	11.5	135.3	3.22	0.32	226.23	NANS
174	Male	52	98.25	31.9	16.6	674.66	15.02	2.41	680.6	NANS
175	Female	40	56.1	23.08	13.5	20.14	8.89	0.84	599.16	NANS
177	Male	77	59.35	21.67	14.3	289.87	10.57	2.12	761.39	NANS
178	Male	45	107.05	33.41	15	485.96	26.74	3.42	934.72	NANS
179	Female	49	82	29.16	12.5	27.47	12.62	1.18	607.99	NANS
181	Male	55	99.25	26.53	14.2	200.13	17.35	1.44	1274.09	NANS
201	Female	41	62	22.77	12.4	18.7	16.09	3.31	807.51	NANS
205	Female	49	61.7	26.22	9.9	12.49	6.73	1.2	1134.65	NANS
206	Female	52	73.15	27.07	13.6	83.55	12.36	0.65	898.65	NANS
210	Female	35	69.8	28.1	13.8	25.54	10.03	0.48	509.8	NANS
211	Female	60	54.2	21.28	12.5	134.94	13.56	2.64	1860.55	NANS
224	Male	43	97.2	31.27	15.6	143.9	10.77	3.95	725.03	NANS
226	Female	52	63.1	22.41		80.89	16.1	0.37	1355.79	NANS
240	Female	48	58.1	22.53	13	43.19	7.19	0.84	713.58	NANS
241	Male	79	66.85	25.22	13.9	165.97	8.82	2.53	532.64	NANS
245	Male	58	93.85	28.02	14.5	208.24	15.28	3.34	788.14	NANS
246	Male	44	94.4	27.76	12.2	7.23	9.01	2.57	1052.49	NANS
267	Male	66	70.65	25.67	15.5	66.55	9.5	0.46	902.57	NANS
270	Male	65	80.6	26.84	16	182.41	11.07	2.62	1167.26	NANS
271	Male	62	95	30.12	16.5	101.17	8.4	1.72	776.32	NANS
282	Male	73	91.15	29.13	14.1	61.14	15.69	6.22	980.97	NANS
283	Male	65	78.5	29.04	16.1	96.21	10.74	1.38	648.21	NANS
285	Male	77	68.8	22.11	14.5	175.52	10.91	2.52	978.64	NANS
287	Female	39	69.8	25.33	12.3	13.55	7.16	1.1	909.92	NANS
302	Female	43	67.35	22.69	11.2	57.65	11.07	0.17	1689.49	NANS
303	Female	41	73.5	30.05	12.2	9.4	14.24	0	670.75	NANS
305	Male	59	90.6	28.98	15.7	255.13	8.63	2.56	744.65	NANS
314	Female	61	76.5	28.07	13.9	143.98	8.53	2.81	712.43	NANS
317	Male	48	79.8	27.64	13.6	121.05	10.12	2.8	695.75	NANS
334	Female	31	47.1	18.54	14	43.07	9.25	1.72	974.99	NANS
342	Male	56	87.7	27.22	12.7	232.78	7.54	1.88	519.98	NANS
343	Female	31	68.4	24.58	11.8	19.14	10.41	2.2	518.66	NANS
344	Female	42	63.7	24.73	12.7	85.8	4.76	1.2	549.51	NANS
368	Male	45	72.2	27.99	13.3	135.44	5.29	0.47	638.87	NANS
371	Female	50	85.75	31.19	11.6	130.53	8.43	2.21	610.99	NANS
373	Female	45	88.1	33.69	13.8	330.86	10.77	6.71	929.96	NANS
374	Male	61	86	29.69	14.7	56.32	12.95	1.54	934.2	NANS
380	Male	26	84.3	26.61	14.8	15.18	10.87	4.48	716.26	NANS
381	Male	48	84.3	26.61	13.8	57.21	15.09	4.89	2180.92	NANS
384	Female	69	70.45	27.49	14	218.76	6.87	0.33	631.49	NANS
385	Male	54	73.2	25.66	14.3	27.88	12.73	2.03	885.24	NANS
386	Male	49	105.45	34.08	14.4	58.48	8.96	3.4	770.9	NANS

401	Female	45	62.3	24.96	13	164.21	10.54	0.88	764.72	NANS
402	Male	43	67.6	25.2	14.7	81.2	16.26	1.69	707.66	NANS
404	Female	60	66.6	26.21	13.5	79.37	9.87	1.18	488.31	NANS
405	Male	41	83.3	26.44	14.3	41.91	11.5	5.35	955.82	NANS
406	Male	48	89.35	31.73	16	15.92	19.88	7.89	1147.1	NANS
407	Female	70	68.45	25.89	12.7	96.54	8.43	1.43	1253.01	NANS
409	Male	49	82.75	25.97	15.3	199.69	16.1	1.07	1129.41	NANS
411	Female	62	84.5	34.9	14.1	71.71	9.79	2.56	617.93	NANS
413	Female	45	61.6	21.62	12.8	84.46	9.02	1.71	556.97	NANS
414	Male	50	86.1	26.14	14.1	87.53	14.49	1.31	1305.36	NANS
415	Male	52	84.7	26.29	14.8	96.51	18.88	0.46	1258.98	NANS
416	Male	47	92.75	29.47	14.8	92.02	18.02	2.51	940.19	NANS
418	Female	32	66.9	25.62	12.5	13.55	7.26	2.26	750.37	NANS
435	Male	47	99.05	28.94	14.2	131.87	14.41	5.95	699.14	NANS
439	Female	44	61.1	21.02	14.1	58.2	5.47	0.86	670.05	NANS
441	Female	66	56	21.71	13.8	53.12	10.91	1.42	2609.64	NANS
442	Male	37	72.6	24.15	14.9	91.75	11.08	1.49	924.24	NANS
444	Female	46	78.1	32.13	12.7	100.19	16	0.47	862.31	NANS
445	Male	27	84.7	28.63	15.7	153.11	9.61	0.9	1349.82	NANS
454	Male	45	98	31.18	15.4	137.4	8.59	2.21	837.57	NANS
467	Female	71	81.25	33.26	13.4	226.99	12.42	1.15	792.62	NANS
469	Male	33	88.35	26.82	14.6	141.44	22.14	1.68	732.1	NANS
471	Male	63	74.85	26.65	15.1	30.55	13.64	0.57	1289.52	NANS
473	Female	35	58.55	22.37	12	76.2	13.89	2.44	895.73	NANS
474	Female	32	52.55	20.45	12.8	147.98	20.75	2.14	1036.28	NANS
478	Male	47	84.75	25.45	14.3	44.53	26.09	0.94	2246.6	NANS
480	Male	63	75.5	22.37	15	176.79	10.35	1.88	1174.13	NANS
484	Female	47	68.4	23.72	13.7	12.29	29.23	0.41	1192.41	NANS
486	Male	26	71.6	22.55	14.5	130.27	14.49	5.46	2204.24	NANS
487	Female	38	58.95	23.38	12.8	26.87	8.95	2.27	458.92	NANS
488	Female	24	62.95	23.26	13.9	115.59	6.33	0.44	590.6	NANS
489	Male	41	79	26.83	14.3	71.39	16.8	0	1570.17	NANS
490	Male	40	81.2	28.8	14.8	88.49	12.59	3.54	718.44	NANS
502	Male	48	59.3	24	14.5	89.58	9.79	1.37	723.99	NANS
503	Male	49	93.6	32.16	15.2	295.89	14.22	0.74	1136.13	NANS
504	Female	52	70.2	28.66	14.2	182.92	12.88	2.16	756.29	NANS
505	Female	42	71.9	27.06	13.7	24.04	9.95	1.45	484.24	NANS
506	Female	47	63.1	26.92	10.4	9.42	10.21	1.77	752.29	NANS
508	Female	51	74.5	31.41	14	116.71	7.77	0.89	539.71	NANS
509	Female	62	84.6	33.13	14	262.89	8.52	1.15	395.35	NANS
510	Female	54	63.85	23.74	14.7	187.04	11.51	2.23	902.97	NANS
513	Female	32	54.3	20.41	13.1	30.13	9.34	2.46	824.24	NANS
515	Female	54	87.35	29.19	13.7	57.72	9.92	1.61	586.43	NANS
516	Male	56	84.9	28.2	14.3	94.61	14.2	1.86	826.54	NANS
519	Female	48	74.1	24.2	12.1	41.32	8	0	771.54	NANS
521	Female	33	80.1	31.49	12.4	44.31	20.38	0.57	1789.47	NANS
525	Female	47	85.55	30.6	10.6	11.17	5.92	0.97	588.11	NANS
526	Female	51	76	26.74	12.6	39.3	6.32	0.78	903.82	NANS
534	Female	51	94	33.87	13	58.16	11.44	3.14	862.31	NANS
535	Male	45	72.4	22.8	15.4	126.61	18.43	5.35	1681.63	NANS
536	Male	49	109	35.71	15.5	679.25	25.07	5.86	1575.21	NANS
539	Male	49	98.4	32.84	16.1	325.72	9.72	4.98	971.72	NANS
540	Female	26	68.6	24.69	12.4	8.67	22.77	0.27	912.52	NANS
542	Male	60	105.35	31.08	15	182.56	15.61	2.26	1560.84	NANS
545	Female	20	64.1	25.91	12.8	38.47	15.74	3.23	905.62	NANS
547	Female	55	65.5	27.83	13.4	208.69	9.1	0.98	752.21	NANS
548	Female	29	51.15	20.78	12.9	114.73	10.53	3.04	1882.01	NANS
552	Female	55	60.75	29.01	13.7	58.52	6.43	0.83	741.11	NANS

554	Female	45	65.05	27.43	13.1	121.48	5.74	0.62	470.49	NANS
555	Male	51	85.2	29.76	16.6	107.64	10.68	6.03	1604.05	NANS
557	Female	23	67.3	22.91	13.3	21.04	7.86	1.76	619.66	NANS
558	Female	44	57.3	22.67	12.3	12.57	13.21	1.61	1214.46	NANS
568	Male	21	101.3	31.79	14.5	113.28	12.89	1.73	1104.21	NANS
570	Female	66	60	23.58	13.3	122.35	13.58	0.44	2306.24	NANS
573	Female	48	71.3	23.71	13.8	68.05	15	4.78	897.8	NANS
574	Male	56	91	27.96	15.5	142.74	17.46	3.76	667.99	NANS
577	Male	56	91.95	28.89	15	94.8	10.9	1.75	908.54	NANS
579	Male	64	91.1	29.48	16.5	284.36	11.71	1.22	1116.75	NANS
581	Female	46	67.6	26.74	14.1	89.51	6.72	0.79	717.43	NANS
582	Male	44	90	27.81	15	290.6	15.51	0.86	1366.74	NANS
585	Female	57	66.9	24.07	14.5	293.54	12.79	2.73	544.94	NANS
586	Male	55	101.8	29.91	15.1	208.49	20.52	2.84	1148.33	NANS
588	Male	65	79.5	27.09	14.8	124.59	10.79	1.15	1040.49	NANS
589	Female	49	50.3	20.86	9.3	10.76	14.77	2.02	1404.55	NANS
590	Female	52	76.8	28.52	13.3	191.34	9.3	0.38	922.5	NANS
591	Male	28	67.95	21.81	14.1	270.05	19.91	2.2	1440.03	NANS
593	Male	57	75.2	28.44	14.6	254.29	19.7	0.36	1200.34	NANS
594	Female	29	57.4	23.23	13.9	32.3	13.38	1.38	1231.11	NANS
595	Male	51	104	32.1	13.3	111.36	12.98	3.28	576.02	NANS
602	Female	64	96.45	36.08	13.7	187.37	12.76	1.94	543.79	NANS
605	Female	39	83.3	29.83	12.9	15.81	11.11	2.97	865.76	NANS
606	Male	54	107.7	33.24	14.9	636.47	10.27	1.83	469.95	NANS
607	Female	46	53.15	21.24	12.3	36.34	3.41	1.02	298.7	NANS
608	Female	67	76.1	30.48	12.9	36.79	9.61	0.6	1254.73	NANS
611	Female	56	66.55	26.62	15.3	36.92	8.01	2.17	809.87	NANS
612	Female	49	65	22.84	13.8	89.01	13.65	3.01	983.42	NANS
613	Male	44	90.3	30.35	15.9	293.84	17.51	3.59	914.59	NANS
617	Male	60	99.9	35.14	15.5	139.23	9.72	1.69	827.09	NANS
622	Female	40	90.6	33.64	15.2	115.75	5.85	1	361.85	NANS
624	Female	58	86	30.08	12.8	86.45	15.24	4.64	1253.48	NANS
625	Female	43	65.6	23.21	11.1	14.65	10.36	0.51	946.52	NANS
626	Male	51	99.4	32.09	15.4	199.74	10.58	2.61	435.99	NANS
627	Female	30	59.4	21.32	14.7	33.25	7.81	2.45	579.48	NANS
628	Female	39	79.4	30.67	14.8	86.62	11.05	0.92	843.97	NANS
634	Female	46	80.3	31.17	14.6	381.28	4.24	1.37	294.44	NANS
637	Female	67	60.9	25.68	14.4	77.93	11.23	5.22	562.28	NANS
638	Female	44	64.2	24.43	13.4	14.54	10.58	0.76	768.53	NANS
639	Male	44	90.1	29.02	15.8	234.18	23.82	4.3	1547.63	NANS
640	Female	61	58.9	21.2	12	45.56	10.89	0.12	1033.72	NANS
642	Female	42	67.9	23.61	13.6	39.14	19.11	2.29	714.24	NANS
646	Female	48	95.7	38.09	14.2	40.65	10.06	0.77	652.15	NANS
648	Male	36	113.3	33.5	15.3	167.6	8.89	0.83	791.51	NANS
652	Female	40	77.35	25.73	12.1	94.6	18.53	1.42	722.61	NANS
653	Male	51	86.45	28.07	14.7	202.57	18.27	1.3	1868.49	NANS
654	Male	58	115.4	33.36	15.8	85.18	13.73	2.91	1069.7	NANS
659	Male	29	68.6	22.5	14.2	137.53	10.94	2.76	642.79	NANS
667	Female	51	66.7	25.92	12.6	35.72	12.97	2.21	1707.27	NANS
669	Male	24	107.85	31.68	16	237.45	17.32	7.96	739.4	NANS
671	Male	61	101.3	34.85	15.7	172.77	12.66	1.93	1292.58	NANS
676	Male	41	93.1	25.25	15.9	327.49	9.63	2.49	540.23	NANS
677	Female	41	83.6	30.71	14.7	138.88	6.12	1.08	978.97	NANS
678	Female	58	67.8	22.55	13.9	122.31	9.36	1.57	1357.88	NANS
681	Male	33	96.1	27.48	16.3	88.41	7.81	3.57	354.31	NANS
683	Female	36			14	41.21	11.09	3.23	838.33	NANS
684	Female	43			14.3	33.32	16.19	3.22	1029.34	NANS
685	Male	48	98.95	33.84	15.2	341.05	11.88	3.27	435.65	NANS

689	Female	20	76.9	24.22	15.3	20.45	21.34	2.82	601.79	NANS
701	Female	38	71.5	25.92	13.5	28.77	8.76	2.54	734.89	NANS
702	Female	37	78.5	29.65	14.2	18.71	14.98	2.75	881.21	NANS
703	Female	38	80.5	31.84	15.1	47.58	7.97	3.87	1010.86	NANS
706	Male	35	84.35	29.22	15.3	182.92	18.77	2.32	1566.46	NANS
707	Female	35	71.9	25.6	13.3	112.48	11.98	1.32	714.5	NANS
708	Female	44	65.2	25.44	12.3	26.53	13.46	9.06	699.06	NANS
711	Female	46	68.7	28.6	13.1	43.88	6.59	0.05	1728.19	NANS
712	Male	37	103.3	33.08	16.9	148.65	17.32	6.37	1201.39	NANS
716	Female	40	51.3	18.82	14	75.4	9.83	4.5	619.09	NANS
717	Female	43	75.1	26.58	12.8	26.21	11.67	0.98	940.09	NANS
719	Male	25	87	28.77	16.3	197.9	8.05	0.3	641.43	NANS
723	Female	44	82.2	27.12	13.5	83.65	19.88	3.38	1157.28	NANS
734	Male	32	85.85	26.06	14.9	112.2	13.25	1.82	1172.82	NANS
736	Male	38	82	26.59	15.5	164.97	15.09	2.65	946	NANS
737	Male	43	84.7	25.1	14.4	172.11	11.02	2.32	446.83	NANS
738	Female	30	74.7	27.91	14.1	11.78	9.61	3.08	778.86	NANS
740	Female	43	65.6	25	13.8	79.81	8.04	1.26	434.32	NANS
741	Male	47	85.2	28.34	15.2	146.82	9.04	1.21	1830.01	NANS
742	Female	39	80.7	29.01	13	7.29	5.49	0.73	522.61	NANS
743	Male	50	117.1	37.21	14.7	99.83	18.37	4.58	912.52	NANS
745	Female	47	69.7	24.29	13.3	114.02	7.12	0.99	724.58	NANS
746	Female	35			14.6	90.1	6.88	0.86	1068.96	NANS
748	Female	35	90.3	31.1	13.1	61.95	18.23	2.98	807.42	NANS
750	Female	41	65	25.45	13.5	111.87	7.94	0.48	457.86	NANS
751	Male	39	92	29.5	15	49.4	11.08	0.79	659.77	NANS
753	Male	29	61.3	21.64	15.3	31.07	7.64	1.04	973.22	NANS
754	Female	24	59.5	23.1	14.2	56.78	10.43	1.41	491	NANS
756	Female	51	81	33.5	17.1	158.73	10.86	3.32	583.33	NANS
767	Male	45	75.3	23.01	14.7	326.43	20.99	3.16	1912.81	NANS
771	Female	43	111.6	37.25		271.47	17.09	2.36	756.44	NANS
773	Male	44	89.55	29.82	15.6	108.53	14.95	2.81	1012.52	NANS
777	Female	36			14.3	71.57	8.59	1.44	488.95	NANS
778	Female	31	68	24.86	13.8	38.14	10.21	1.44	893.24	NANS
779	Female	42	90.05	36.16	13	13.97	6.67	1.29	924.54	NANS
780	Male	77	82.7	28.96	14.9	134.14	4.57	0.71	658.48	NANS
782	Female	23	51.7	20.07	12.5	38.77	9.26	1.53	857.3	NANS
803	Male	23	68.1	22.24	16.4	158.52	52.12	2.35	1251.69	NANS
804	Male	26	71.5	23.11	13.8	155.32	8.62	2.83	1021.38	NANS
807	Male	27	70.9	23.42	15.6	106.9	17.22	6.06	1393.82	NANS
810	Male	24	89.3	25.78	14.6	181.65	25.75	3.13	1111.78	NANS
811	Female	25	80.95	30.92	14.8	96.13	6.39	1.07	430.12	NANS
812	Male	26	88.7	28.34	15.5	221.19	8.6	2.49	605.92	NANS
815	Male	26	76.3	24.41	14.3	132.94	11.88	1.74	869.06	NANS
816	Female	29	83	26.58	15	57.56	12.79	1.03	1874.89	NANS
818	Male	26	95.15	30.79	13.9	192.09	11.09	2.01	1052.99	NANS
822	Female	27	63.2	22.47	13.8	68.61	11.29	1.28	541.69	NANS
825	Male	37	102.5	30.51	14.4	123.88	14.21	2.94	1477.23	NANS
826	Male	34	91.4	32.38	15.4	156.61	13.98	2.22	1022.81	NANS
827	Female	25	75.5	21.25	14.6	42.19	5.25	1.46	381.04	NANS
828	Female	34	69.5	24.77	11.7	24.79	8.25	0.29	805.33	NANS
829	Female	32	67.5	23.25	12.8	49.39	12.06	3.07	1314.25	NANS
832	Female	35	69.7	25.63	12.9	30.32	16.18	0	1261.35	NANS
834	Male	23	91	26.02	14.9	79.13	17.17	0.86	980.17	NANS
837	Female	37	71.5	27.58	12.9	24.13	11.07	0.84	566.83	NANS
838	Male	36	79.8	28.24	15.7	187.87	15.97	3.09	997.86	NANS
840	Male	29	123.5	37.12	15	334.04	7.85	2.68	467.62	NANS
841	Female	27	73.6	24.88	13.8	70.09	7.95	1.06	1117.08	NANS

842	Male	32	95.1	30.02	15	190.85	5.83	2.47	698.48	NANS
844	Male	32	95.8	27.4	15.7	95.31	18.98	3.46	1562.02	NANS
847	Male	36	85.2	28.97	16.9	217.96	13.08	3.42	758.3	NANS
849	Male	37	75.7	24.63	14.7	154.46	7.24	1.22	579.57	NANS
854	Male	31	104.7	33.99	16.2	212.38	16.24	4.69	1092.54	NANS
855	Female	28	59.8	25.55	11.6	35.36	9.81	2.61	699.81	NANS
857	Female	33	106.8	39.71	13.5	36.13	4.93	1.36	262.37	NANS
859	Female	32	68.8	27.39	12.2	22.98	9.58	2.83	639.56	NANS
860	Female	21	55.6	22.13	13.6	55.89	4.18	0.29	490.15	NANS
873	Male	26	71.3	25.05	14	175.72	7.39	1.28	1080.01	NANS
874	Female	27	64.6	21.71	13	80.57	15.72	0	547	NANS
875	Male	26	73.4	21.68	15.4	137.81	17.61	4.37	1141.79	NANS
882	Female	34	73.2	26.47	13.5	62.04	9.11	2.13	566.57	NANS
894	Female	22	45.75	17.35	13.5	80.65	9.8	0.67	652.58	NANS
903	Male	35	97.65	30.48		181.59	10.27	3.71	649.79	NANS
904	Male	31	86.8	24.45	14.7	127.52	3.82	1.62	605.12	NANS
907	Male	64			11.8	146.75	6.55	0.25	1134.37	NANS
909	Male	57	75.3	25.31	14.1	222.31	8.82	2.8	680.5	NANS
910	Male	31	65.45	22.25	13.9	109.64	14.71	3.29	956.94	NANS
913	Male	28			14.1	194.62	11.56	3.43	1236.2	NANS
921	Female	52	63.5	21.59	14.1	231.95	10.42	3.2	450.22	NANS
922	Male	47	87.6	27.8	13.9	16.96	15.71	4.06	898.58	NANS
926	Female	35	78.6	29.05	14.3	36.85	7.56	2.57	725.22	NANS
935	Female	55	61.3	28.96	12.7	82.05	6.59	1.36	720.79	NANS
937	Female	59	86.5	35.96	13.4	46.3	4.89	0.52	877.18	NANS
939	Male	27	90.3	28.53	15.8	80.97	11.78	1.27	1053.16	NANS
945	Female	19	61.95	21.06	11	19.95	12.75	0.65	1388.35	NANS
946	Male	18	80.5	27.43	14	69.49	15.4	1.61	1163.97	NANS
947	Male	37	86.2	28.87	15	159.41	18.07	2.21	1482.87	NANS
950	Male	33	85.2	25.98	15.9	122.89	12.53	3.29	1055.64	NANS
951	Female	20	56.6	21.43	12.8	52.48	12.8	2.2	978.63	NANS
952	Female	31	60.8	20.79	13.4	107.44	6.51	2.65	241.06	NANS
955	Female	18	62.5	22.52	12.7	39.34	15.84	6.81	1415.21	NANS
956	Male	20	83.8	25.55	14.7	68.03	19.26	9.2	1072.72	NANS
959	Female	58	70.4	23.15	14.1	66.42	16.34	2.26	1526.57	NANS
960	Male	44	80.1	28.52	16.8	187.18	9.76	2.48	604.44	NANS
969	Male	36	80.9	24.16	15.5	45.57	8.25	3.39	1209.2	NANS
970	Male	38	81	27.19	16.7	109.92	6.36	2.35	851.76	NANS
971	Male	39	86.2	30.11	16.3	57.06	9.43	2.74	865.86	NANS
974	Male	36	58.4	18.02	15.4	132.37	18.49	3.13	1441.66	NANS
975	Male	20	71	23.08	14.7	87.81	16.51	3.9	1560.73	NANS
978	Male	64	102.35	32.63	15.7	143.05	7.27	2.11	497.29	NANS
980	Male	29	101.6	37.91	13.9	117.44	7.68	2.31	814.73	NANS
981	Male	33	116.3	29.37	15.4	196.71	12.61	4.77	969.26	NANS
1003	Male	82	76.1	26.64	15.5	375.03	9.02	1.88	684.91	NANS
1007	Female	33	82.6	28.85	13.3	95.61	31.57	1.06	971.09	NANS
1008	Male	68	73.8	26.53	14.2	327.52	7.58	2.25	276.05	NANS
1010	Female	72	61.4	26.75	13.7	69.83	6.3	3.26	373.67	NANS
1011	Male	33			13.4	50.63	11.85	1.29	1206.45	NANS
1013	Male	34	79	24.27	14.8	227.37	15.79	3.54	1032.84	NANS
1016	Female	75	63.4	24.46	12	168.15	10.09	1.97	1612.08	NANS
1019	Female	81	83.1	31.63	12.8	56.39	11.42	1.96	609.41	NANS
1020	Female	68	72.9	27.34	12.2	32.76	12.06	1.67	1050.93	NANS
1021	Female	81	71.1	25.9	12.8	65.6	11.5	1.44	665.84	NANS
1022	Female	71	88.2	34.54	11.9	71.57	12.62	1.57	664.86	NANS
1025	Male	28	75.3	24.25		97.86	9.94	2.45	793.43	NANS
1026	Male	62	102.3	35.03	14.1	200	11.19	1.13	548.68	NANS
1029	Female	66	80.5	27.4	11.7	46.62	5.89	1.39	1156.01	NANS

1030	Male	47	105.15	30.33	16	90.45	15.21	1.74	819.44	NANS
1031	Male	49	80.2	23.84	16.2	89.16	25.41	0.05	877.77	NANS
1033	Male	53			14.5	119.75	5.73	1.07	483.03	NANS
1034	Female	63	66.4	31.11	13.5	207.28	7.63	0.72	1457.92	NANS
1036	Male	37	83.4	26.35	15.3	46.01	21.91	5.29	1144.47	NANS
1038	Male	36	103.1	32.91	14	185.37	11.84	4.55	893.66	NANS
1042	Female	30	75.1	27.06	12.1	19.48	8.89	2.78	618.32	NANS
1044	Male	31	69.15	27.25	14.7	91.75	12.21	1.14	990.69	NANS
1046	Female	27	79.3	30.37	12.4	32.88	9.35	1.05	278.25	NANS
1050	Female	27	47.9	21.58	13.1	12.58	7.79	0.86	772.77	NANS
1051	Male	27	71.9	24.62	14.6	276.49	8.13	2.7	731.8	NANS
1052	Male	37	80.4	27.18	14.8	735.54	12.62	4.01	874.05	NANS
1053	Female	75	61	27.15	12.5	47.61	8.34	1.82	728.54	NANS
1054	Male	74	94.3	29.43	14.5	201.72	10.08	1.83	675.76	NANS
1055	Female	77	65	26.27	12.6	116.27	22.3	2.57	1665.22	NANS
1057	Female	67	72.5	28.18	10.3	203.51	12.75	3.62	627.69	NANS
1059	Male	35	86	27.02	15.1	76.41	15.96	12.07	609.6	NANS
1064	Female	77	54.6	23.91	13.1	55.15	7.27	1.7	390.62	NANS
1065	Male	34	65	20.42	14	110.28	3.34	0.08	976.72	NANS
1066	Male	48	71.8	27.06	11.4	10.74	8.74	3.72	763.84	NANS
1068	Male	69	82.3	28.34	15.4	159.51	7.6	1.08	587.05	NANS
1069	Male	31	78.2	25.39	14.7	235	13.59	1.41	1674.51	NANS
1073	Female	71	66.5	27.68	14.1	82.04	5.57	0.75	490.92	NANS
1074	Male	71			13.1	242.96	9.1	1.16	926.93	NANS
1077	Male	31	100.6	27.12	13.1	146.54	35.29	1.11	1318.04	NANS
1079	Male	32	84.6	27.31	13.5	106.31	9.08	2.34	1009.74	NANS
1081	Male	27	80.3	26.58	15.1	223.81	12.63	5.81	556.67	NANS
1084	Male	27	96.2	28.26	14.2	47.88	6.89	0.86	540.67	NANS
1085	Male	49	90.15	28.14	15.9	137.19	16.32	3.31	944.83	NANS
1086	Female	61	60.55	23.51	12.4	84.59	10.99	1.4	649.15	NANS
1089	Male	48	82.3	28.82	14.3	566.07	10	1.9	1158.04	NANS
1092	Male	56	103.55	31.78	14.9	152.35	12.04	4.34	686.42	NANS
1094	Male	21	100.1	31.24	15	42.88	11.38	2.06	1543.29	NANS
1101	Female	75	49.7	20.69	13.3	17.23	10.91	2.93	538.77	NANS
1105	Female	61	73.6	29.78	12.8	303.28	11.13	1.26	915.66	NANS
1108	Male	72	82.35	29.35	16.9	88.41	9.19	3.47	733.92	NANS
1113	Male	35	79.8	28.41	14.4	80.04	10.69	3.42	811.84	NANS
1117	Male	24	67.3	21.9	15.3	164.24	13.77	1.38	873.23	NANS
1124	Female	68	48.4	20.87	13.6	123.39	10.02	2	2323.7	NANS
1127	Male	27	95.3	32.36	14.6	225.49	13.97	0.31	1135.5	NANS
1128	Male	18	66.9	21.57	14.4	107.61	15.49	2.9	1538.82	NANS
1129	Female	57	76.8	27.47	15.2	68.82	12.83	2.74	1123.33	NANS
1130	Male	27	92.9	28.7	13.9	89.63	15.51	1.53	1321.16	NANS
1131	Female	26	60.2	22.52	12.5	40.01	9.64	0.56	614.58	NANS
1133	Female	24	60.7	21.51	14.4	26.11	11.08	0.61	426.27	NANS
1136	Female	69	69.3	29.26	13.1	91.96	9.16	1.8	642.86	NANS
1137	Male	75	76.85	27.92	13.7	94.4	5.85	0.22	936.46	NANS
1150	Male	21	74.05	20.88	15.1	121.84	12.3	1.45	1568.49	NANS
1151	Male	21	88.8	27.59	13.3	125.42	13.19	1.85	1546.85	NANS
1152	Female	21	57.45	21.2	13	76.02	6.71	1.22	811.13	NANS
1155	Male	32	86.7	28.47	15.3	48.11	4.09	2.54	399.66	NANS
1156	Male	49	85.1	28.17	14.2	169.9	8.2	2.4	734.86	NANS
1604	Male	72	79.6	26.29	14.4	59.96	10.29	1.91	647.19	NANS
1607	Female	77	65.3	26.09	12.2	129.59	12.6	2.2	1056.43	NANS
1608	Female	76	69.8	25.21	14.5	257.81	21.22	6.59	1183.66	NANS
1609	Male	56	92	31.28	15.8	162.76	7.04	0.89	1151.73	NANS
1610	Female	67	53.9	23.77	15.6	23.3	10.76	3.79	1069.32	NANS
1612	Female	81	56.4	22.65	13.7	71.31	17.4	2.23	950.84	NANS

1620	Male	73	86.3	27.39	16.7	477.28	12.54	2.4	863.44	NANS
1621	Male	50	90.8	28.5	15	181.2	13.39	4.09	1352.38	NANS
1622	Male	76	66.15	22.13	13.2	195.78	8.99	1.81	1079.99	NANS
1634	Female	71	59.6	22.77	13.4	74.65	16.53	0	1118.01	NANS
1636	Female	64	66.55	24.59	14.6	136.93	9.02	1.05	661.59	NANS
1639	Male	71	99.7	29.23	15	64.86	16.35	3.48	888.85	NANS
1641	Male	69	87.6	24.84	14.2	145	14.83	2.44	994	NANS
1644	Female	50	60	21.46	13.4	135.68	19.56	1.13	953.63	NANS
1646	Male	51	103	31.61	15.4	128.79	12.48	2.54	923.6	NANS
1648	Female	60	80.2	25.34	14.4	95.24	10.23	1.26	1211.65	NANS
1649	Female	27	69.8	24.7	13.7	35.36	9	0.7	723.31	NANS
1650	Female	63	69.5	28.05	12.9	91.2	11.51	0.03	1700.04	NANS
1651	Male	52	89.4	27.72	15.6	96.06	10.73	0.56	1456.23	NANS
1652	Female	66	76.15	25.41	13.2	37.11	14.63	1.37	2736.49	NANS
1653	Female	65	73.6	32.11	14.1	84.09	8.66	3.87	602.39	NANS
1655	Male	46	110.65	34.69	15.6	610.26	14.07	3.98	926.32	NANS
1658	Female	75	69.1	26.62	13.9	125.29	10.9	0.93	979.76	NANS
1669	Male	27	89.55	30.3	13.3	73.85	13	1.9	740.52	NANS
1673	Female	41	57.6	19.72	12.6	19.15	11	1.23	909.76	NANS
1677	Male	54	85.7	28.73	14.3	229.8	7.9	3.52	980.02	NANS
1681	Female	47	60.45	25.03	13.3	11.85	13.85	2.36	416.52	NANS
1682	Female	52	57.2	25.25	12.9	38.46	6.36	1.58	311.34	NANS
1687	Male	64	88.6	31.81	14.9	175.91	9.43	2.22	448.88	NANS
1688	Male	40	85.1	27.13	16.1	188.7	15.19	4.72	1235.63	NANS
1689	Female	56	92.7	33.2	11.9	21.21	7.96	0.28	447.76	NANS
1702	Male	68	95	29.32	16.3	362.45	11.6	1.42	1191.6	NANS
1704	Female	45	66.2	24.26	14.2	42.69	6.56	0.77	657.74	NANS
1706	Male	41	79.6	23.23	14.3	175.1	19.42	2.73	1353.05	NANS
1707	Male	29	71.4	24.28	15.8	152.19	11.83	4.56	854.2	NANS
1709	Male	66	80.2	25.31	17.5	57.1	15.02	1.87	1086.79	NANS
1711	Female	63	128.3	47.99	12.5	708.06	9.64	1.2	617.85	NANS
1712	Male	72	96.55	33.25	15.8	634.5	11.38	3.38	639.62	NANS
1715	Male	37	85.7	25.48	15.4	197.49	21.91	4.01	1611.64	NANS
1717	Female	43	80.4	29.86	13.7	12.51	12.89	2.35	1069.13	NANS
1718	Female	65	53.1	23.6	13.7	66.9	6.68	0.74	699.81	NANS
1719	Male	43			16.4	79.97	8.62	3.85	687.67	NANS
1735	Male	63	57.9	21.63	11	336.07	14.73	5.89	999.76	NANS
1736	Female	67	71.9	27	14.1	237.87	9.35	3.64	535.69	NANS
1738	Male	45	69.85	21.39	14.3	180.63	9.89	3.37	1613.33	NANS
1739	Male	26	91.9	29.4	14.9	148.39	14.8	2.13	766.24	NANS
1742	Male	28	99.9	31.82	16.2	160.96	11.41	2.96	1624.11	NANS
1745	Male	53	98.6	35.31		84.84	12.71	3.54	921.72	NANS
1746	Male	30	108.6	32.43	16.1	182.46	17.97	3.39	1192.31	NANS
1747	Male	78	79.7	25.44	14.3	131.08	7.03	1.48	456.34	NANS
1748	Female	41	76.7	28.17	13.6	39.73	24.84	0.79	792.86	NANS
1749	Male	56	88.15	30.15	15.9	306.62	11.1	1.62	696.11	NANS
1758	Male	42	102.45	28.26	16.4	97.46	13.94	3.63	840.41	NANS
1767	Female	60	69.5	29.27	15.6	105.61	10.01	4.05	1087.77	NANS
1770	Female	48	80.9	29.64	13.4	155.51	11.22	2.04	1057.28	NANS
1771	Female	45	81.8	30.71	12.3	15.24	6.97	1.86	528.42	NANS
1774	Female	39	59	23.81	14.4	59.59	11.27	1.49	657.22	NANS
1776	Female	25			13.8	50.36	9.52	1.14	929.33	NANS
1778	Male	65	85.6	29.31	13.2	651.6	12.93	5.91	718.33	NANS
1780	Male	47	79.8	29.63	17.4	199.6	25.13	2.27	1060.25	NANS
1781	Female	52	70	30.14	12.5	41.7	7.52	0.93	639.67	NANS
1782	Male	82	66.6	25.07	16.3	68.28	5.37	0.89	414.31	NANS
1784	Male	76	106.4	38.38	14.3	246.81	11.91	0.67	610.05	NANS
1787	Male	67			14.9	40.55	9.51	2.92	2061.69	NANS

1790	Male	33			16.1	628.71	12.94	5.71	1259.13	NANS
1804	Male	50	90.3	29.02	15	166.82	6.81	2.3	620.08	NANS
1815	Female	74	71.35	25.43	11.9	111.99	9.16	1.04	635.33	NANS
1822	Female	33	68.1	27.8	13	72.88	3.94	1.48	960.27	NANS
1823	Male	74	72.3	27.82	10.1	7.68	15.82	0.95	1440.35	NANS
1824	Female	46	62	22.39	13.5	62.14	21.17	0.52	1223.82	NANS
1834	Male	62	119.15	46.37	14.3	220.51	12.85	0.83	1102.68	NANS
1835	Female	61	73.9	31.12	13.6	52.12	7.68	1.32	478.97	NANS
1836	Female	65	68.8	28.82	14	81.79	5.86	2.06	504.41	NANS
1838	Female	59	73.7	27.07	13.7	106.87	9.06	1.49	585.1	NANS
1841	Female	64	77.1	28.15	13.1	142.04	9.12	2.12	579.34	NANS
1845	Female	29	52.5	20.64	14	39.77	8.21	2.45	523.43	NANS
1848	Female	27	61.3	21.26	13.9	15.11	16.35	0.81	992.75	NANS
1849	Female	49	64.6	25.3	13.8	92.54	14.88	1.18	827.94	NANS
1850	Female	40	53.8	22.8	13	25.07	8.79	1.64	577.63	NANS
1869	Male	38	109.5	31.65	15.6	164.22	19.62	2.93	999.84	NANS
1870	Female	43	65.6	27.23	14.1	195.83	7.35	1.7	776.92	NANS
1872	Male	58	96.15	31.79	15.2	116.52	9.58	1.79	764.34	NANS
1877	Female	26	85.1	34.79	13.7	69.22	6.42	0.94	674.58	NANS
1881	Male	66	79.5	26.56	15.9	112.75	6.94	2.63	600.21	NANS
1882	Female	44	76.2	28.33	14.2	260.31	17.91	1.99	1324.36	NANS
1884	Male	41	78.2	26.56	16.2	126.96	8.92	1.31	745.68	NANS
1886	Male	37	113.5	39.46	16.2	203.9	7.68	3.19	761.55	NANS
1904	Male	38	68.8	25.64	15.7	96.68	8.43	2.37	931.61	NANS
1905	Male	47	78.2	24.16	16.2	94.64	14.55	1.25	1669.93	NANS
1907	Male	47	95.5	30.83	14.9	84	21.04	2.12	1260.01	NANS
1908	Female	41	69.3	27.69		76.25	9.42	1.21	960.67	NANS
1917	Female	58	67.9	23.69	14	47.91	16.05	2.04	1448.24	NANS
1918	Male	41	102	27.67	13.8	184.95	26.09	9.07	1225.39	NANS
1935	Female	42	58.1	25.52	13.1	75.25	16.03	3.05	915.93	NANS
1939	Female	28	66.95	23.44	13.1	42.42	10.22	0.94	859.75	NANS
1942	Male	38	83.55	29.43	15.5	174.6	13.74	1.25	1321.36	NANS
1943	Male	43	93.4	30.19	15.8	271.9	9.93	2.09	895	NANS
1948	Female	39	67.75	25.22	14.1	102.29	14.89	2.86	1092.94	NANS
1951	Male	56	84.3	29.14	15.7	155.37	25.68	4.79	1236.76	NANS
1953	Female	68	69.6	30.12	13.5	248.06	9.61	2.3	1390.73	NANS
1955	Female	32	80.55	32.93	14.8	14.88	13.97	1.08	560.92	NANS
1969	Male	61			15.1	249.99	10	1.35	1164.97	NANS
1973	Female	50	69.05	26.67	14.1	156.99	8.62	0.44	832.13	NANS
1976	Female	47	74.4	27.53	12.2	17.45	7.04	2.22	666.44	NANS
1977	Female	70	86.65	32.41	13.8	171.87	7.36	0.68	966.69	NANS
1978	Male	61	137.95	37.46	17.9	122.57	12.56	2.66	1468.62	NANS
1983	Male	64	88.5	29.95	16.6	99.3	8.94	1.69	1112.04	NANS
1984	Male	59	120.5	38.07	15	353.91	15.04	0.84	868.5	NANS
1986	Female	55	60.4	23.51	13.9	220.7	7.23	1.27	1838.58	NANS
1988	Female	45	86	26.81	12.6	34.07	10.47	1.32	1119.01	NANS
1989	Male	60	97.5	27.24	14.7	786.84	29.29	3.43	1155.27	NANS
2001	Female	59	83.9	32.61	15	71.48	7.95	1.18	1124.37	NANS
2003	Male	61	87	32.31	15.3	241.84	10.1	0.45	746.8	NANS
2004	Female	40	69.9	26.05	14.6	93.01	14.62	5.23	807.08	NANS
2005	Male	41	71.9	23.91	14.4	37.43	12.76	3.27	899.54	NANS
2007	Female	39	60.3	27.02	14.1	216.68	9.18	1.19	638	NANS
2010	Female	43	59.75	23.49	13.4	273.67	8.53	2.31	686.68	NANS
2011	Male	33	78.7	25.93	16.4	79.32	12.58	3.46	1641.34	NANS
2012	Female	26	74.45	27.58	14.1	82.03	11.73	1.58	1225.25	NANS
2013	Male	48	80.6	26.14	14.4	639.83	12.27	1.52	1214.12	NANS
2014	Female	39	69.6	24.75	13.5	12.83	19.01	3.02	604.18	NANS
2015	Female	51	68.55	26.45	13.1	10.43	9.21	1.02	438.55	NANS

2020	Male	53	63.9	21.83	14.7	342.61	16.13	1.97	1166.38	NANS
2022	Male	28	80.5	24.87	13.9	131.61	17.88	4.15	1188.79	NANS
2023	Female	45	69.1	23.49	13.1	17.42	12.79	0.74	857.86	NANS
2025	Female	53	62.3	25.37	12.6	27.47	12.23	3.22	1104.6	NANS
2034	Female	63	64.5	25.51	13.6	32.68	7.15	1.56	914.35	NANS
2041	Male	52	97.8	30.63	14.8	406.24	14.4	1.54	955.34	NANS
2042	Male	62	94.8	28.81	14.2	576.53	12.24	3.06	813.72	NANS
2051	Female	21	78.4	24.91	13.4	20.24	8.64	0.12	670.74	NANS
2052	Female	75	67.2	26.58	13.6	155.72	7.79	0.97	614.96	NANS
2054	Female	40	56.9	22.12	15.9	33.04	14.77	3.82	705.64	NANS
2060	Female	52	68.8	24.88	13.2	91	12.13	1.44	1355.18	NANS
2062	Female	20	57.9	21.42	12.1	13.48	11.26	0.49	1188.36	NANS
2075	Female	32	98.5	34.9	13.9	10.64	7.16	1.67	713.34	NANS
2077	Female	20	59.5	24.23	13.4	27.93	8.04	1.79	799.96	NANS
2078	Female	51	83.7	33.7	13.2	45.13	13.99	1.14	870.4	NANS
2081	Male	48	119.6	37.75	17	187.46	8.88	1.52	814.41	NANS
2083	Male	55	88.3	26.95	16.1	166.17	12.17	1.01	834.87	NANS
2084	Female	18	43.85	18.11	12.9	40.41	7.98	0.43	509.18	NANS
2091	Female	21	98.3	34.62	13.6	162.06	10.8	0.82	622.79	NANS
2092	Male	20	89.6	28.76	16.6	165.55	10.33	1.63	517.81	NANS
2101	Female	55	98.35	34.39	14.6	68.6	8.07	0.6	586.74	NANS
2106	Male	44	86.3	29.21	15.4	658.04	11.26	3.68	846.7	NANS
2107	Female	58	89.8	35.57	12	126.71	8.08	2.54	579.87	NANS
2110	Female	30	63	26.53	13.8	19.88	12.33	2	530.11	NANS
2115	Female	20	82	30.16	14.1	31.23	11	3.21	848.7	NANS
2116	Male	20	99.4	30.04	15.4	196.77	8.99	4.02	350.98	NANS
2120	Male	23	100.6	32.89	15.6	149.15	7.08	0.92	888.97	NANS
2121	Female	40	68.9	27.05	14.9	103.01	7.47	2.28	739.01	NANS
2124	Female	35	70.1	29.6	14.5	62.14	8.23	1.57	734.28	NANS
2125	Female	50	73.2	30.19		15.41	11.59	3.1	990.42	NANS
2129	Female	46	68.1	27.91	10.5	15.83	9.2	2.45	433.78	NANS
2131	Female	54			13.9	192.97	9.78	3.34	597.05	NANS
2134	Male	21	72.3	26.36	15.8	37.71	15.05	1.95	855.58	NANS
2135	Female	50	81.65	22.98	12.3	39.59	6.3	1.79	382.81	NANS
2136	Female	45	82.3	32.72	14	28.96	6.59	2.56	377.9	NANS
2137	Male	46	102.3	31.02	17.2	202.03	17.96	4.47	800.11	NANS
2138	Female	38			12.4	11.98	6.59	0.3	444.78	NANS
2153	Female	43	77	32.94		17.63	6.05	1.48	479.8	NANS
2154	Female	60	113.4	43.8	12.6	181.61	14.37	1.97	926.8	NANS
2160	Female	50	96.3	41.41	13.5	197.39	4.62	0.82	331.37	NANS
2165	Female	54	81.65	29.17	12.9	229.45	9.33	0.88	755.5	NANS
2167	Female	23	61.1	20.9	13	22.81	7.23	3.08	985.42	NANS
2169	Female	30	65.8	24.05	14.3	7.26	6.37	2.36	238.46	NANS
2171	Male	28	58.6	21.14	15.4	208.83	11.14	3.84	570.18	NANS
2172	Female	70			13.4	230.4	9.78	2.92	527.62	NANS
2173	Male	30	95.7	32.5	16	119.61	14.06	3.46	946.6	NANS
2176	Male	60	86.1	30.29	14.5	172.91	7.39	2.72	770.49	NANS
2180	Male	24	104.1	29.14	15.3	107.72	9.26	3.34	724.83	NANS
2181	Female	50	77.1	27.85	13.3	24.34	12.03	0	1388.71	NANS
2183	Female	50	54.1	22.37	13.4	30.25	8.27	0.76	656.23	NANS
2184	Female	24	53.5	20.56	14	24.6	14.27	1.59	963.87	NANS
2185	Male	28	105.5	27.97	15.7	686.99	9.22	1.1	1518.22	NANS
2188	Female	41	91.2	32.12	13.6	52.53	10.69	2.73	1221.58	NANS
2191	Female	20	83.5	32.78	14.1	35.16	12.37	3.74	814.45	NANS
2194	Female	46	121.1	48.33	11.9	15.75	12.63	0.54	722.31	NANS
2201	Male	20	74.3	23.35	15	79.37	17.88	6.85	896.51	NANS
2203	Male	54	97.7	33.1	14.2	250.11	16.81	2.59	1063.54	NANS
2204	Male	52	96.25	29.91	15.9	85.93	13.36	3.43	1572.13	NANS

2205	Female	23	49	19.36	13.8	42.31	9.37	1.87	930.09	NANS
2208	Male	46	73.3	22.23	16.4	141.18	6.89	1.65	683.01	NANS
2210	Male	43	81.5	24.85	16.9	269.77	13.73	1.92	1134.19	NANS
2211	Male	55	111.3	33.97	14.8	83.46	11.93	1.18	1085.36	NANS
2212	Female	30	75.6	26.19	12.6	85.03	12.59	1.88	792.57	NANS
2213	Male	52	103.6	33.14	17	698.14	12.07	0.79	1477.18	NANS
2214	Female	38	76.8	25.25	12.7	15.74	9.98	1.77	724.78	NANS
2218	Male	40	93.2	30.33	16.1	203.46	11.13	0.54	959.97	NANS
2219	Female	40	80.6	30.94		40.26	12.91	0.93	1237.68	NANS
2235	Male	34	80.5	27.69	16.4	75.29	13.79	2.42	674.45	NANS
2236	Male	37	75	25.41	13.5	119.12	12.34	1.76	856.2	NANS
2238	Female	32	71.75	23.75	12	30.75	10.4	1.34	777.26	NANS
2240	Male	51			15.5	293.18	17.02	4.22	1268.11	NANS
2243	Female	35	84.3	30.89	14.2	86.95	15.84	0.26	1187.41	NANS
2244	Male	44	102.9	29.97	17.5	156.4	16.69	2.41	1148.46	NANS
2246	Male	38	113	35.99	16.7	134.64	6.78	1.6	709.61	NANS
2249	Female	46	64.4	23.94	14.8	23.06	12.2	1.67	985.89	NANS
2255	Male	47	92.8	33.56	16.3	136.5	21.73	4.76	694.55	NANS
2256	Female	24	66	25.02	14.7	41.7	8.63	0.63	616.83	NANS
2272	Male	45	93.2	29.09	15.2	77.58	17.49	4.28	695.23	NANS
2276	Male	31	78	23.81	14.8	170.96	12.63	1.79	614	NANS
2277	Male	39	97.6	32.24	15.9	181.73	16.94	3.3	1332.17	NANS
2278	Female	43	100.5	36.04	12.5	9.7	8.13	1.96	546.71	NANS
2282	Male	48	80.5	27.34	16.6	84.19	23.77	2.32	1336.2	NANS
2284	Male	51	83.4	30.63	17	202.95	9.12	4.89	668.23	NANS
2286	Male	47	80.8	27.15		162.03	14.4	2.58	855.74	NANS
2288	Male	45			15.8	210.17	24.39	6.7	981.57	NANS
2303	Female	44	68.8	24.7	13.9	73.6	9.73	1.7	247.77	NANS
2304	Male	30	80.95	26.61	18.9	349.77	10.82	2.5	749.3	NANS
2308	Female	43	72.55	23.69	11.2	7.21	9.33	2.2	719.81	NANS
2310	Male	18	66.5	20.82	15.9	124.42	17.99	1.82	1002.21	NANS
2311	Male	24	75.6	26.44	15.7	108.09	14.34	2.53	789.48	NANS
2313	Male	21	86	27.33	14.1	72.38	9.06	2.47	1063.75	NANS
2314	Female	47	73	24.5		26.16	13.56	3.42	1752.78	NANS
2315	Male	24	79.3	24.26	14.7	91.06	15.08	2.69	1309.83	NANS
2316	Male	31	83.8	23.96	16.8	116.52	13.48	4.3	1013.61	NANS
2317	Male	25	80.6	26.62	15.5	59.86	14.64	2.93	1078.97	NANS
2335	Male	59	79.4	26.71	15.4	198.06	13.34	1.84	1359.51	NANS
2341	Male	54	74.2	24.56	16.7	165.41	17.59	4.27	1617.63	NANS
2342	Male	20	81.4	26.34	17.2	32.03	11.9	4.01	570.05	NANS
2344	Female	23	72.1	28.31	13.9	78.89	9.22	1.2	1065.89	NANS
2347	Female	44	67.85	26.7	15	34.78	15.79	0.36	820.94	NANS
2348	Male	42	86.65	31.33	15.8	198.14	9.19	2.76	1090.49	NANS
2352	Female	26	63.1	24.31	12.8	17.98	6.57	2	595.87	NANS
2353	Male	22	81.45	24.75	16	69.16	14.73	5.5	1537.73	NANS
2354	Male	59	96.5	33.55	15.1	119.44	12.47	2.51	910.35	NANS
2355	Male	53	108.45	30.3	14	10.85	18.05	1.21	1168.88	NANS
2356	Male	65	87.9	28.03	16.6	588.87	12.91	5.64	1363.6	NANS
2367	Female	56	64.4	25.67	12.6	15.52	14.7	3.98	853.31	NANS
2368	Female	61	74.8	28.47	14.8	185.54	10.03	0.96	1340.54	NANS
2369	Male	45	88	28.41	15.6	121.94	13.05	2.42	863.85	NANS
2370	Male	50	87.4	28.18	15.5	227.39	19.1	3.97	798.39	NANS
2372	Female	21	63.9	22.06	14.3	24.54	19.63	6.94	1160.52	NANS
2373	Male	21	101.9	29.9	15.2	94.09	22.48	3.56	1654.67	NANS
2378	Female	39	81.8	30.45	14.5	296.78	7.8	0.31	426.78	NANS
2385	Female	51	55.6	22.33	13.7	49.94	11.57	0	1387.81	NANS
2388	Female	24	53.4	21.64	12.8	21.21	9.98	4.58	804.13	NANS
2393	Male	21	65.3	20.52	14.8	50.18	24.9	3.72	1597.78	NANS

2395	Male	19	81.3	24.52	17.9	109.31	18.15	2.51	1972.28	NANS
2396	Female	37	73.2	27.65	11.6	14.88	16.7	1.11	1310.59	NANS
2401	Male	56	143.55	44.16	13.6	321.81	20.85	4.42	1483.02	NANS
2403	Male	20	72.4	21.04	14.8	68.57	24.87	2.84	1547.39	NANS
2404	Female	26	54.35	22.48	13.3	29.18	21.84	0.18	658.5	NANS
2406	Male	27	64	22.15	14.5	103.44	10.22	2.53	1704.91	NANS
2409	Female	28	59.65	22.34	12.8	28.96	18.4	0.97	1016.96	NANS
2411	Male	19	70	24.92	14.1	180.08	16.71	3.29	1442.4	NANS
2412	Male	19	62.8	20.07	12.8	245.47	15.38	4.36	1313.17	NANS
2418	Male	18	108.3	32.2	15.3	33.49	8.35	1.76	493.8	NANS
2421	Female	19	69	25.31	10.8	16.72	12.08	2.06	1025.2	NANS
2424	Male	19	73.9	23.88	15.5	78.81	11.86	2.13	1127.47	NANS
2428	Male	20	92.5	28.74	15	140.29	9.65	3.6	831.33	NANS
2437	Female	25	69.6	26.07	13.5	47.22	7.01	2.29	670.05	NANS
2438	Male	23	89.4	26.81	15.9	52.87	17.67	5.56	2280.24	NANS
2439	Female	23	53.95	19.21	13.9	114.16	5.85	1.22	662.03	NANS
2442	Female	19	70.6	25.16	13.9	50.57	11.67	2.09	822.42	NANS
2443	Female	19	60.1	21.78	13.7	25.73	11.16	1.46	944.68	NANS
2444	Male	22	82.5	24.69	16.1	132.94	19.41	0.57	965.59	NANS
2445	Male	21	80.3	24.73	17.1	74.26	15.34	0.98	1876.66	NANS
2446	Male	22	105.9	27.65	15.6	35.96	35.98	1.77	1735.23	NANS
2451	Male	25	89.6	25.49	15.5	90.79	24.74	4.76	1516.23	NANS
2452	Male	24	88.8	26.69	16.2	54.21	7.57	2.78	771.68	NANS
2454	Female	20	66.2	24.35	13.9	54.41	7.34	1.8	546.67	NANS
2456	Female	20	96.4	31.02	14.1	50.19	4.68	0	622.4	NANS
2458	Female	21	76.95	24.31	13.6	85.78	10.81	5.1	890.93	NANS
2470	Female	28	64.7	23.31	13.1	73.99	8.66	2.41	526.63	NANS
2474	Male	28	93.4	28.35	14.9	185.31	15.37	3	1852.32	NANS
2476	Female	22	63.8	21.69	13.8	60.68	5.06	0	927.32	NANS
2484	Male	26	70.4	22.47	15.4	68.11	31.55	3.72	1698.83	NANS
2487	Male	26	86.4	24.81	15.9	61.11	18.89	1.38	561.33	NANS
2489	Female	23	76.7	31.28	13	27.65	6.86	1.44	526.12	NANS
2491	Female	23	63	21.15	13.1	27.64	16.25	1.62	904.79	NANS
2492	Female	22	67.5	24.06	15.4	68.23	9.67	0.69	523.45	NANS
2496	Male	33	79.7	24.44	15.3	117.27	11.1	5.45	967.23	NANS
2501	Male	25	74.55	22.14	16.1	17.77	19.35	5.59	1082.31	NANS
2505	Female	66	70.7	29.2	12.5	63.21	10.53	3.31	618	NANS
2506	Female	73	71.2	31.43	14.5	131.41	7.19	0.9	561.66	NANS
2507	Female	74	90	33.1	14.2	75.68	7.41	1.34	482.86	NANS
2508	Female	74	61.3	30.15	17	60.48	11.16	1.48	1293.66	NANS
2509	Female	23	75.1	27.16	13	58.25	12.84	2.86	626.29	NANS
2511	Male	21	79.7	22.53	14.8	162.27	9.75	2.24	1185.61	NANS
2512	Female	55	81.2	29.86	13.1	153.03	11.94	2.18	613.51	NANS
2513	Female	57	78.6	28.73	14	67.55	8.99	1.58	1009.53	NANS
2516	Male	76	71	24.54	14.7	227.18	8.42	0.7	661.59	NANS
2517	Male	84			15	45.46	6.29	2.76	385.35	NANS
2525	Female	22	68.1	21.96	14.1	35.91	16.99	3.12	569.91	NANS
2526	Male	25	93.3	25.52	17.3	249.94	20.56	3.78	2493.77	NANS
2527	Male	21	95.5	25.35	15.3	60.12	16.6	3.43	1669.26	NANS
2529	Male	43	79.55	23.86	16.4	69.73	16.78	3.98	1198.92	NANS
2530	Male	21	85.3	28.9	18	143.02	7.87	0.39	547.42	NANS
2535	Male	37	88.1	29.07		231.07	22.75	3.51	975.32	NANS
2536	Female	72	81.1	31.4	12.6	146.97	11.75	1.94	570.01	NANS
2538	Male	37	107.9	32.11	17	297.81	15.09	5.48	1032.69	NANS
2541	Male	18	69.1	21.83	15.8	72.69	17.28	5.81	954.1	NANS
2545	Male	72	77.35	27.94	15.4	204.06	13.81	2.45	1385.19	NANS
2547	Male	19	81.8	25.56	15.7	109.55	13.15	2.81	902.19	NANS
2548	Male	38	84.45	25.38		93.3	16.31	3.24	890.39	NANS

2553	Male	19	79.9	23.1	16.8	107.6	12.39	3.16	831.97	NANS
2554	Male	18	75.2	24.08	15.9	43.93	17.99	3.28	1354.78	NANS
2557	Male	20	76.8	25.6	13.8	60.26	11.49	1	821.26	NANS
2565	Male	24	101.3	32.48	14.5	168.09	10.91	1.72	1196.81	NANS
2575	Male	77	81.4	30.08	13.8	143.71	17.05	0.78	725	NANS
2577	Male	21	66.8	22.48		121.99	22.79	9.88	751.82	NANS
2579	Male	22	69.4	21.66	15.5	63.69	13.57	2.15	807.49	NANS
2582	Female	22	70.95	24.32	15.3	78.57	7.97	1.28	637.93	NANS
2586	Male	23	85.2	27.69	15.7	306.15	5.56	1.22	860.97	NANS
2589	Male	22	93.35	29.27	15	114.47	14.38	9.34	996.6	NANS
2593	Male	20	69.95	21.23	15.9	79.24	10.01	2.13	1595.53	NANS
2595	Male	39	91.8	31.47	16.8	135.42	9.76	1.74	1327.38	NANS
2596	Female	77			11.2	87.37	8.11	0.98	666.25	NANS
10120	Female	46	97.2	34.13	13.4	42	8.2786	0	840.3	NDNS
10124	Female	33	72.7	26.92	13.7	44	12.8332	2.8492	937.2	NDNS
10130	Male	43	101.4	35.69	14.1	257	18.1305	4.2811	978.6	NDNS
10132	Male	53	78.4	26.95	13.9	9	13.9714	1.5132	1312.4	NDNS
10211	Female	42	67.4	24.46	10.4	11	15.4419	0.6183	1064	NDNS
10225	Male	22	78.9	24.72	15.2	110	13.8829	3.2925	861.8	NDNS
10229	Female	41	107.65	47.91	13.5	25	9.3092	1.0716	1116.6	NDNS
10301	Female	45	97.95	41.65	14.1	98	11.716	1.9599	1026.3	NDNS
10303	Male	44	74.55	24.91	14.9	20	21.7756	0.9721	886	NDNS
10311	Female	36	60.95	23.68	12.5	25	10.214	0.7514	1091.2	NDNS
10312	Male	37	73.5	25.54	16	87	12.3254	0	952.2	NDNS
10320	Female	47	100.35	39.84	14	93	17.6957	0.7752	946.7	NDNS
10326	Female	21	51.25	18.94	14	59	4.4638	1.0862	250.8	NDNS
10328	Female	33	71.3	26.43	13.2	25	10.9391	1.3252	758.8	NDNS
10329	Male	43	73.85	22.31	17	402	6.5369	2.241	672.1	NDNS
10331	Male	59	68.5	21.52	16.7	219	8.3502	2.0942	582.2	NDNS
10338	Male	24	114.7	34.55	14	80	8.3644	2.6801	752.3	NDNS
10340	Female	31	70.35	23.97	14	50	10.0803	2.4285	1016.8	NDNS
10401	Male	39	63.9	24.81	15	17	16.8064	4.0819	1251.3	NDNS
10402	Female	36	94.2	40.99	12.9	226	18.1342	0.5512	1397.9	NDNS
10403	Female	24	62.5	22.8	14.1	103	9.0779	2.5112	604.6	NDNS
10412	Male	36	67.45	22.75	14.1	119	24.1811	2.6109	1783.4	NDNS
10422	Female	39	63.7	22.22	12.9	40	11.0421	2.8815	1017.9	NDNS
10440	Female	38	79.2	32.01	12.8	38	10.5054	1.0929	813.9	NDNS
10506	Male	56	117.25	38.79	15.1	71	9.062	3.6838	539.4	NDNS
10522	Male	43	120.5	36.84	15.1	139	16.8176	4.6866	1263	NDNS
10527	Male	61	86.6	27.61	15.8	223	10.6531	1.4554	1134	NDNS
10528	Male	53	90.6	28.24	14.3	149	16.63	6.0378	1364.3	NDNS
10534	Female	30	74.25	30.07	13.6	30	12.8852	0	1080.1	NDNS
10537	Female	26	54.35	20.61	14	32	5.7255	0.8602	501.5	NDNS
10538	Female	26	61	22.02	12.7	48	6.4255	0.2986	650.2	NDNS
10540	Male	51	65.1	20.98	13.6	47	11.0873	0.623	1226.1	NDNS
10602	Female	62	94.8	36.41	14.4	50	11.5945	1.4193	969.6	NDNS
10614	Female	39	80.1	30.11		29	9.5955	5.7091	365.5	NDNS
10616	Female	62	57.2	20.91	14.7	48	5.78	2.2442	751.3	NDNS
10617	Male	38	75.9	26.67	16.6	79	13.2273	4.1287	1067.1	NDNS
10618	Female	29	71.3	28.91	14.4	26	15.1575	0.2537	642.9	NDNS
10624	Female	43	54.25	21.66	13.9	39	5.835	1.3246	824.8	NDNS
10626	Male	25	107.3	38.68	14.3	131	7.3534	3.1805	748.8	NDNS
10706	Male	47	65.2	19.93	14.8	13	13.1344	2.0773	1120.8	NDNS
10709	Male	41	75.8	26.81	14.8	19	15.504	0.9964	1214	NDNS
10710	Female	64	85.7	36.87	13.8	46	8.0746	1.3154	680.7	NDNS
10713	Male	45	63.7	22.34	14.9	47	10.2036	3.5814	890.8	NDNS
10717	Female	62	85.15	33.45	12.9	70	11.3358	2.8056	979.8	NDNS
10719	Female	35	67.3	27.85	14.6	57	5.4599	1.2075	509.8	NDNS

10722	Male	61	82.1	25.9	13.9	178	14.3775	2.8925	978	NDNS
10726	Male	43	122.35	37.12	14.7	134	14.594	1.0503	1040.9	NDNS
10731	Male	38	93.6	30.72	14.5	125	10.3246	4.4448	382.3	NDNS
10734	Male	48	81.5	26.54	14.2	63	12.0903	2.9145	852.4	NDNS
10736	Female	36	69.85	27.03	15.1	23	5.952	1.3092	499.4	NDNS
10810	Male	31	100.1	31.02	15.2	74	13.3361	5.5374	772.2	NDNS
10815	Male	37	82	26.28	14.7	140	15.857	4.7425	879.6	NDNS
10816	Female	39	102.6	39.56	13.4	24	10.0002	0.9226	465.5	NDNS
10905	Female	30	57.05	21.33	13.3	62	18.4591	9.6032	793.9	NDNS
10909	Male	32	84.4	31.55	16.7	77	30.1935	6.6249	2696.8	NDNS
10912	Male	46	66.2	23.32	15.5	114	19.857	2.0777	1116.8	NDNS
10913	Female	53	60.85	22.31	12.5	32	12.3763	0.7449	1233.1	NDNS
10922	Female	53	76.7	31.02	13.2	142	9.2157	0.9804	660.7	NDNS
10928	Female	40	76.75	25.76	13.6	25	10.6853	2.0052	1101.5	NDNS
10936	Male	59	87.5	25.76	14.6	81	17.3296	3.531	1343.3	NDNS
10937	Male	63	82	27.27	13.8	97	24.4546	1.2647	2283.2	NDNS
11001	Male	50	79.3	26.12	15.2	32	15.679	3.8739	1347.5	NDNS
11002	Male	40	79.75	24.16	14.1	119	7.9932	1.564	855.7	NDNS
11004	Male	53	74.3	23.33	15.4	137	12.2881	2.2414	1121.5	NDNS
11006	Female	58	79.2	32.19	12.9	273	29.4573	1.8952	617.4	NDNS
11007	Male	41	65.4	22.52	15.5	94	12.901	2.5308	921.2	NDNS
11009	Male	21	54.25	20.32	15.4	86	6.1932	2.3741	495	NDNS
11010	Male	40	80.4	26.07	14.4	73	14.397	4.9096	1130.3	NDNS
11016	Male	42	87.35	31.41	16.1	58	11.2679	2.3807	1113.3	NDNS
11017	Male	62	69.05	26.56	15.1	194	6.6936	2.1727	458.8	NDNS
11022	Male	55	77.8	26.61	16.4	68	8.9991	3.028	941.5	NDNS
11029	Female	30	53.65	19.95	14.6	36	9.2229	0.5219	554.7	NDNS
11103	Female	25	103.9	36.27	12.9	42	8.9547	0	971.9	NDNS
11106	Female	32	77.3	28.97	14	75	7.3515	2.2865	599.8	NDNS
11109	Female	54	53.45	20.66	13.5	78	12.3853	2.4254	926.7	NDNS
11116	Male	25	109.55	35.05	15.6	169	20.7877	5.8142	1777	NDNS
11120	Male	45	88.4	25.8	16.5	100	14.4734	3.5841	1298.6	NDNS
11132	Male	49	94.25	26.14	15.8	237	12.312	2.7133	737.9	NDNS
11133	Female	63	71.55	24.83	12.4	36	12.0852	0.774	838.1	NDNS
11134	Female	39	56.4	21.45	13.6	16	18.913	2.8966	781	NDNS
11207	Male	35	97.05	28.65	16.2	56	5.8032	0.2969	779	NDNS
11210	Male	19	55.3	20.24	16.6	45	5.0576	1.4488	255	NDNS
11212	Female	39	70.05	24.75	12	14	1.6355	0.2828	170.8	NDNS
11216	Male	29	78.2	24.27	15.8	118	9.002	1.3995	886.5	NDNS
11221	Female	61	80.7	37.04	12.6	81	10.398	1.4272	611.8	NDNS
11228	Female	20	57.55	23.97	12	41	9.6196	0.6151	737.2	NDNS
11231	Female	20	50.55	19.37	14	15	6.0187	0.1985	586.3	NDNS
11240	Female	44	59.95	22.62	11.8	31	14.4487	2.7531	700.1	NDNS
11301	Female	42	63.5	22.7	12.9	24	12.9349	2.8344	615.9	NDNS
11306	Male	59	87.9	29.89	17.3	93	12.2223	0.5245	925	NDNS
11330	Male	43	104.4	30.69	13.8	57	11.826	1.7824	1351.8	NDNS
11417	Female	34	57.7	22.61	13.7	45	5.2402	0.9353	387.7	NDNS
11421	Male	42	101.8	32.53	14.9	32	13.4256	2.4486	1559.2	NDNS
11422	Female	35	63.4	26.49	14.6	43	7.1532	1.9882	851.6	NDNS
11430	Female	54	62.6	33.65	15.1	78	5.9817	0	882.7	NDNS
11510	Male	30	70.85	23.37	14.1	83	14.1052	2.8967	1609.5	NDNS
11512	Male	56	95.3	30.01	14	26	18.0476	1.6236	1216.8	NDNS
11516	Male	60	80.2	26.77	15.5	183	10.1349	2.0286	1208.8	NDNS
11517	Female	29	75.3	31.75	13.9	15	6.2281	0.779	669.4	NDNS
11522	Male	39	101.85	39	13.9	38	12.6115	3.3324	877.1	NDNS
11536	Female	23	71.15	27.09	14.3	118	5.6698	1.7609	371	NDNS
11538	Female	21	78.8	30.92	13.7	86	6.3285	0.9312	879.3	NDNS
11539	Female	37	52.8	21	13.4	19	9.9404	0.6062	592.6	NDNS

11607	Male	60	75.5	27.93	15.2	88	17.7091	4.3843	938.6	NDNS
11625	Male	36	84.2	26.37	14.1	99	16.7669	3.6849	1062.1	NDNS
11628	Male	51	90.9	29.15	17.1	211	6.5318	1.0382	623.4	NDNS
11631	Male	49	83.85	26.46	15.1	108	16.0708	6.4725	841.4	NDNS
11632	Male	24	73.7	23.79	14.6	79	8.1609	3.2279	482.6	NDNS
11725	Male	56	85.85	30.97	13.3	22	10.4943	3.7947	837	NDNS
11735	Male	41	78.65	25.32	14.7	413	20.1502	6.6394	1029.6	NDNS
11803	Male	40	93.45	30.74	16.1	91	13.0588	4.8045	1361.6	NDNS
11804	Female	62	65.65	25.77	13.1	66	8.5288	1.5375	742.8	NDNS
11807	Female	40	64.6	26.16	13.6	37	7.2152	1.3713	697	NDNS
11810	Female	34	74.35	27.01	13.6	39	11.2841	1.2118	537.9	NDNS
11814	Female	59	65.5	26.56	12.9	46	8.8579	1.2595	1205.6	NDNS
11817	Female	48	60.7	23.83	13.3	22	5.8703	0.7092	1206.6	NDNS
11819	Male	25	53	17.94	15.4	37	7.6231	2.6468	468.4	NDNS
11821	Male	41	62.4	21.59	16.1	173	13.9797	3.7701	1034.1	NDNS
11908	Female	63	62.4	24.13	13.2	39	10.0477	1.7822	1319.2	NDNS
11915	Female	24	76.95	27.23	13.4	14	4.5182	1.0509	492.2	NDNS
11921	Female	59	82.55	32.05	12.1	75	8.416	0.8222	628.2	NDNS
11929	Female	25	70.8	26.7	13.5	29	5.3368	0.4596	755	NDNS
11931	Female	55	69.7	26.59	13.9	60	11.1735	0.6839	899.2	NDNS
11940	Female	47	58.1	20.61	12.6	85	6.129	1.5581	533.8	NDNS
12004	Female	53	72.15	26.7	14.1	77	11.9741	3.2797	607.1	NDNS
12005	Female	45	54.3	20.01	12.8	22	7.4218	1.9265	838.8	NDNS
12006	Male	42	66.75	23.11	14.4	107	12.9198	3.1901	445.3	NDNS
12007	Male	39	72.5	25.15	16.2	80	5.4189	1.9181	576	NDNS
12011	Male	20	69.2	19.64	16.8	80	4.8438	2.1294	307.6	NDNS
12012	Female	62	62.7	24.93	12	43	11.5453	1.5955	836.6	NDNS
12021	Female	34	61.5	22.88	13.9	26	3.5212	0.8954	270.9	NDNS
12025	Female	38	95.35	37.11	14.4	150	6.2709	0.4571	473.6	NDNS
12026	Female	58	87.45	31.95	13.4	25	11.7057	2.9257	382.8	NDNS
12032	Male	46	76.6	26.32	15	608	10.7243	1.992	754.2	NDNS
12037	Female	62	90.1	32.82	14.3	196	7.2044	0.8107	321.3	NDNS
12107	Male	41	80.6	24.19	16.1	107	6.1576	1.3444	1050.7	NDNS
12121	Female	53	66.95	26.57	14.8	53	11.6876	1.8514	838.4	NDNS
12122	Male	46	79.1	25.11	14.9	80	13.5011	3.8602	967.6	NDNS
12123	Female	61	66.95	26.25	12	43	7.7022	0.5632	415.9	NDNS
12124	Male	58	80.9	27.33	15.7	42	11.4826	1.7439	1352.7	NDNS
12127	Male	45	64	22.74	14.1	76	7.9778	2.2081	731.1	NDNS
12130	Female	29	58	22.38	14.2	61	10.4717	3.3383	935.3	NDNS
12131	Male	32	85.3	29.12	16.4	106	15.7855	3.0939	1327.2	NDNS
12132	Female	24	103.85	34.88	14.1	65	12.4525	1.6712	458.1	NDNS
12139	Male	27	102.9	30.79	14.4	105	13.2547	3.5074	959.8	NDNS
12208	Female	52	66.55	29.23	14.1	98	6.4601	0.4735	612	NDNS
12210	Female	32	84.4	31.65	13.4	38	7.7469	0.5638	734.3	NDNS
12211	Female	35	53.1	20.52	13.3	36	12.3243	2.4695	649.7	NDNS
12213	Female	29	67.7	23.92	16.2	215	8.0362	1.627	614.9	NDNS
12214	Male	63	79.1	26.74	12.7	20	11.1822	2.886	860.6	NDNS
12215	Male	26	98.8	28.16	15.2	69	11.3636	3.6751	708.7	NDNS
12223	Male	34	71.1	23.81	14	67	20.0811	3.7116	1323.6	NDNS
12227	Female	37	53.2	20.26	12.6	13	2.7496	0.9162	254.3	NDNS
12228	Female	62	66.5	24.19	13.6	43	10.0753	1.3887	982.9	NDNS
12303	Male	44	99.8	35.28	15	322	8.5595	2.954	671.2	NDNS
12306	Male	22	83.6	28.46	16.5	89	4.1952	1.4641	550.6	NDNS
12320	Female	26	57.8	22.05	12.8	17	9.4942	1.9826	731.2	NDNS
12325	Male	27	88.75	29.62	14.6	119	14.2772	4.0872	987.5	NDNS
12331	Female	38	59.4	22.56	14.9	28	10.6178	0.6746	941.9	NDNS
12332	Male	58	73.35	23.31	15.1	299	12.624	3.141	639.9	NDNS
12335	Female	38	69	25.19	13.9	40	11.8087	4.1607	859.5	NDNS

12338	Female	31	65.8	21.87	13.3	58	10.825	1.8603	826.6	NDNS
12339	Male	38	83.2	23.05	15.7	90	15.8602	4.2537	1237.6	NDNS
12403	Male	56	83.8	26.87	12.9	20	12.4745	2.9021	2288.3	NDNS
12405	Male	62	97.85	30.56	14.4	236	19.6194	0.1171	824.5	NDNS
12408	Female	43	75.3	27.33	12.7	27	12.3376	0.385	806	NDNS
12410	Female	45	55.7	22.14	13.2	17	12.0945	2.7019	579.6	NDNS
12418	Female	35	61.2	19.96	13.5	12	10.8442	0	1077.5	NDNS
12427	Male	52	80.7	26.64	15.4	66	11.5187	0	894.2	NDNS
12440	Female	53	90.9	33.33	12.1	38	13.4031	2.3982	722.9	NDNS
12501	Male	43	62.4	21.73	14.1	124	18.2061	2.2836	554	NDNS
12504	Male	52	93.4	30.96	15.6	71	12.6747	3.4506	989.2	NDNS
12506	Female	25	65.4	23.27	13	61	5.6305	0	511.3	NDNS
12512	Female	26	59.4	24.25	13.7	43	8.996	2.3017	822.6	NDNS
12522	Male	23	85.55	27.84	14.8	120	24.7036	1.7246	1055.9	NDNS
12536	Female	59	66.8	26.69	13.3	102	12.4172	1.2534	1149.8	NDNS
12538	Male	26	52.8	18.9	16.9	199	10.9884	1.3171	563.2	NDNS
12609	Male	29	72.2	23.12	16	59	16.1219	2.612	1196.4	NDNS
12611	Female	38	66.45	25.21	12.9	37	9.2565	0.9806	1261.7	NDNS
12616	Male	45	114.35	38.16	13.9	65	4.919	1.6474	746.5	NDNS
12618	Female	30	76.6	28.81	13.4	18	10.1241	1.6213	646	NDNS
12625	Female	26	110.25	43.94	16.1	119	7.4436	1.4525	220	NDNS
12628	Male	48	83.5	27.98	15.7	91	5.16	0.8857	588.9	NDNS
12629	Female	42	68.2	23.85	14.3	63	10.5481	1.5444	799.9	NDNS
12704	Female	43	65.55	22	14.9	72	10.9973	1.9244	781.5	NDNS
12710	Female	30	56.5	19.97	12.9	31	6.3688	0	829.6	NDNS
12711	Female	30	86.1	29.09	14.3	37	14.5341	4.0477	1089.2	NDNS
12714	Male	52	90.85	31.57	14.6	262	12.381	0.5855	685.9	NDNS
12721	Female	56	63.3	24.53	12.7	51	15.1091	0.8126	1017	NDNS
12730	Female	56	65.1	26.38	15.5	73	11.1512	0.4582	943.1	NDNS
12731	Female	56	71.25	26.56	13.1	37	15.2335	2.0979	777.2	NDNS
12732	Female	60	63.65	24.36	13.2	160	8.1661	1.4529	691.4	NDNS
12738	Male	46	77.15	24.15	15.5	87	13.8669	3.048	825.4	NDNS
12802	Female	31	108.9	41.19	13.1	23	7.0158	2.3756	693.2	NDNS
12803	Female	35	79.7	34.25	12.9	63	10.8751	0.12	816.1	NDNS
12809	Female	31	59.3	20.84	12.6	44	5.8776	1.6415	490.3	NDNS
12823	Male	39	84.6	25.29	16	24	19.9571	1.3593	1601.4	NDNS
12829	Female	38	103.8	33.76	12.9	38	14.2365	0.1302	1384.4	NDNS
12839	Female	53	66	26.98	13	44	3.2061	0.1	443.1	NDNS
12907	Male	54	65.8	22.25	12.6	24	7.5081	2.3451	831.6	NDNS
12908	Female	38	58.35	24.8	12.6	19	6.4675	1.6576	667.1	NDNS
12923	Male	44	72.4	23.63	16.2	50	7.235	1.8643	945.3	NDNS
12926	Female	53	58.6	22.47	13.7	75	13.6395	4.0287	780.9	NDNS
12930	Female	48	89.05	32.65	13.5	46	9.4282	0.8825	1322.3	NDNS
12932	Male	56	99.55	31.12	15	49	8.9275	1.0241	794.5	NDNS
12934	Female	28	57.55	20.82	15.1	41	8.0408	0	1215.6	NDNS
13008	Male	53	55.1	19.83	15.9	152	15.165	7.1573	1446.8	NDNS
13009	Female	31	70.4	28.09	14.3	112	5.0734	0.598	708.8	NDNS
13014	Female	33	69	26.26	13.3	50	11.1598	0.9636	1065.7	NDNS
13015	Female	28	57.9	22.28	13.2	57	6.8144	1.0085	1178.7	NDNS
13020	Male	43	98.3	29.97	16.4	240	12.6026	2.7252	966.7	NDNS
13028	Male	64	84	28.31	16.1	185	5.4104	1.5432	675	NDNS
13103	Male	39	81.8	25.59	14.2	15	16.5542	2.1517	1454.2	NDNS
13106	Female	35	64.5	21.93	15.3	266	17.2603	1.8979	1420.9	NDNS
13108	Female	59	75.9	28.69	13.7	71	15.2441	3.0077	1070.2	NDNS
13110	Male	22	105	30.76	16	77	14.9333	1.6458	1402.5	NDNS
13114	Male	63	84.8	28.14	15.1	114	9.2912	3.2286	443.4	NDNS
13118	Female	55	79.7	28.2	14.1	47	14.5358	0	1570.7	NDNS
13127	Female	57	57.25	21.65	14.6	16	8.8529	1.2862	849.8	NDNS

13133	Female	47	73.7	28.33	12.9	52	11.3684	0.9966	1033.9	NDNS
13135	Male	44	66.2	21.44	15.1	69	10.9181	0.1685	764.8	NDNS
13136	Male	62	84.1	25.94	15.4	42	24.1418	1.8591	1121.4	NDNS
13139	Male	45	80.1	26.29	17	67	20.1433	0.3645	1232.1	NDNS
13211	Male	45	90.45	27.56	14.8	110	19.8847	2.008	859.7	NDNS
13215	Female	25	54.45	22.68	11.5	10	5.0207	0.3335	579.6	NDNS
13223	Female	29	58.85	20.41	13.1	107	11.245	3.3079	1054.4	NDNS
13228	Female	47	60.1	22.84	13.8	13	11.9526	1.8725	1000	NDNS
13240	Male	55	85.95	27.37	15.8	120	11.8134	1.3038	1299.4	NDNS
13302	Female	58	71.9	27.03	15.3	59	8.9811	1.6226	475	NDNS
13303	Female	39	66.7	22.92	13.8	19	9.5065	2.4262	990	NDNS
13306	Female	53	78.2	28.85	13.3	154	7.0448	1.4172	692.8	NDNS
13307	Female	64	66.25	23.51	14.6	53	8.8874	0.1	823.6	NDNS
13313	Male	50	96.3	32.95	14.7	102	20.2137	3.3276	1328.6	NDNS
13315	Male	39	82.6	26.26	14.1	37	13.4296	1.5243	1213.6	NDNS
13320	Female	41	67.3	24.62	13.8	55	13.7131	1.166	1119.5	NDNS
13324	Male	30	79.5	27.88	14.5	170	14.1399	2.2014	1246.5	NDNS
13327	Male	55	111.8	35.53	16.3	506	13.9543	4.8199	1100.7	NDNS
13405	Female	35	87.5	33.3	13.7	35	10.0583	1.759	697.3	NDNS
13408	Male	42	85	30.03	16.5	18	4.4219	0.9738	325.5	NDNS
13417	Male	31	66.9	21.15	16.8	175	10.1729	1.1517	1107	NDNS
13423	Male	57	88	31.8	15.8	183	20.6574	4.3248	1067.4	NDNS
13503	Female	34	69.65	28.18	12.4	5	13.5819	2.2742	1254.7	NDNS
13504	Male	33	81.15	28.18	15.2	55	13.5019	0.9562	1316.1	NDNS
13508	Male	19	74.6	23.88	16	19	12.0177	0.8843	1417.1	NDNS
13509	Male	44	90.45	31.1	15.1	92	10.1133	2.8477	1063.7	NDNS
13510	Male	24	72.9	25	15.2	53	11.4975	1.1171	1231.1	NDNS
13514	Female	45	68.3	26.45	13.7	15	6.6104	1.956	476.2	NDNS
13517	Male	39	77.2	25.51	15.3	26	14.9878	2.7901	1145.7	NDNS
13524	Female	23	59.7	21.23	13.2	32	23.4526	2.281	1549.8	NDNS
13525	Male	34	99.95	30.53	14.8	81	27.7032	4.392	1189.1	NDNS
13527	Male	29	90.8	28.79	15.1	30	11.7968	4.7347	777.8	NDNS
13536	Female	47	73.6	25.32	12.5	34	12.8633	1.333	951.1	NDNS
13539	Male	61	72.6	24.26	16.2	176	8.4653	1.3762	956.2	NDNS
13618	Female	22	53.5	20.36	11	8	7.4567	1.2479	905.8	NDNS
13629	Female	39	50.75	20.58	13.8	59	7.2476	1.3157	827.3	NDNS
13710	Female	19	42.8	15.89	13.7	59	5.3001	2.0074	463.6	NDNS
13813	Male	59	117.9	35.26	14.5	170	11.1161	1.2416	1021.9	NDNS
13824	Female	56	67.2	26.87	13	113	10.851	1.2693	1088.3	NDNS
13903	Female	45	71.05	24.69	12.8	36	12.5647	1.7723	1138.4	NDNS
13904	Female	63	45.35	18.15	15.5	13	3.3414	0.6538	164.4	NDNS
13907	Female	36	57.65	22.7	15.6	109	15.1831	3.5706	462.7	NDNS
13937	Female	38	51.2	20.67	12.8	116	9.2939	1.3291	740	NDNS
13939	Female	35	50.1	22.12	14.6	44	10.2088	1.519	625.2	NDNS
14006	Female	46	71.8	25.68	13.4	73	10.1394	2.2782	733.6	NDNS
14101	Female	51	72.8	29.63	13.6	28	17.8188	3.0595	888.4	NDNS
14102	Male	48	88.9	29.76	15.7	624	10.3906	2.8461	922.5	NDNS
14105	Male	36	90.1	27.72	15.6	15	22.9346	3.5453	1555.4	NDNS
14106	Female	63	79.2	32.38	13	180	10.7174	1.9738	907.2	NDNS
14107	Male	54	95.1	27.4	14.8	23	6.5946	1.2525	743.4	NDNS
14112	Female	64	65.2	25.11	12	24	10.0401	4.005	835.6	NDNS
14116	Male	41	75.9	23.48	15.5	87	10.698	2.4975	981.8	NDNS
14117	Female	37	70.9	26.97	13.8	51	4.4197	1.3277	875.2	NDNS
14122	Male	56	86.6	26.33	12.8	211	13.2775	1.918	1243.8	NDNS
14127	Female	39	62	22.79	15.2	58	5.1556	0.657	499.9	NDNS
14306	Female	52	60.2	24.24	12.2	18	5.824	1.736	975.8	NDNS
14310	Female	41	47.9	19.89	13.9	39	11.924	0.7601	1019.3	NDNS
14313	Female	54	86.15	32.19	13.5	121	12.5928	0.5066	551.7	NDNS

14315	Male	19	49.6	16.9	15.3	27	6.183	1.1446	668	NDNS
14317	Male	54	121.6	37.36	14.4	247	12.2227	3.6017	996.6	NDNS
14328	Female	48	77.5	30.81	14.6	28	9.4955	2.2046	559.3	NDNS
14332	Female	62	49.3	21.74	13	10	12.322	0.4876	859.2	NDNS
14336	Female	28	53.3	20.67	12.8	63	8.906	0.3856	909.3	NDNS
14340	Female	55	73.3	27.09	14	52	15.4607	1.2807	1109.7	NDNS
14404	Male	62	63.25	20.05	15.4	71	20.8709	1.867	1210.4	NDNS
14406	Male	53	86.1	26.56	17.4	213	18.7232	2.1959	1783.4	NDNS
14412	Female	48	75.75	30.61	13.3	147	7.947	0.9958	689.2	NDNS
14416	Male	64	103.3	30.93	16.4	110	12.4635	3.4651	1061.8	NDNS
14420	Female	61	63.85	27.89	14.7	257	6.9421	0.9314	448.5	NDNS
14435	Female	31	55.7	22.27	13.5	16	6.5442	1.3414	516.1	NDNS
14506	Male	39	81.95	26.76	13.8	107	21.0866	1.9795	1511.8	NDNS
14509	Female	31	71.6	26.88	12.9	145	3.9613	0.8265	359.7	NDNS
14511	Female	32	67.1	23.55	12.8	45	13.9767	2.3957	857.6	NDNS
14514	Female	43	55.75	21.03	12.8	13	10.0091	3.2836	732.8	NDNS
14515	Female	35	112.5	44.03	13.3	27	4.5311	1.9773	430.3	NDNS
14528	Female	35	105.7	41.37	13.5	64	7.3166	0.3465	946.2	NDNS
14529	Male	39	80.45	27.01	16.2	48	11.5884	0.9478	749.9	NDNS
14534	Male	48	90.6	29.22	16.3	90	13.5826	2.0336	1377.5	NDNS
14536	Female	45	80.7	30.37	13.2	18	14.4029	0	1108.6	NDNS
14537	Male	44	107.6	36.86	16.9	324	15.2256	0.8855	1090.9	NDNS
14539	Male	32	99.25	34.4	14.4	123	13.3112	3.792	1014.2	NDNS
14601	Male	60	96.7	26.48	14.3	72	13.0347	2.4615	1148.7	NDNS
14605	Female	34	70.45	27.78	14.5	31	5.7718	1.9667	498.2	NDNS
14610	Female	32	67.4	25.7	13.4	47	10.0152	1.7957	582.7	NDNS
14613	Female	55	58.5	23.76	14.3	99	7.5097	4.6222	325.4	NDNS
14618	Female	51	60.2	22.81	13.2	34	14.4434	0.6336	1288.8	NDNS
14622	Female	60	66.4	27.06	10.4	5	10.2263	1.6153	955.6	NDNS
14623	Female	44	52.25	19.43	14.1	48	9.9724	2.0565	581.4	NDNS
14624	Male	34	90.05	28.23	15	191	12.7532	5.1071	1060.6	NDNS
14626	Male	50	73	24.07	15.6	37	13.5672	2.0778	855.5	NDNS
14627	Male	43	98.95	30.07	16.3	108	15.2299	2.1221	1314.1	NDNS
14630	Female	33	64.5	23.97	12.4	27	9.3661	0.3387	576.1	NDNS
14714	Female	55	76.7	28.97	15.2	114	8.7958	2.8767	437	NDNS
14716	Female	44	79.6	27.51	14.2	21	8.253	2.0427	1012.1	NDNS
14724	Male	62	99.45	33.99	17.1	222	4.176	1.3591	225.9	NDNS
14725	Male	20	62.9	20.41	16.9	129	8.8898	1.8432	1116.5	NDNS
14730	Male	44	81.6	27.26	15	202	21.7363	1.1374	849.9	NDNS
14802	Male	41	98.7	33.02	14.4	6	11.5368	2.7552	994.3	NDNS
14803	Female	29	58.05	23.67	12.5	45	4.8459	1.4053	294.1	NDNS
14805	Male	28	74.2	21.26	14.4	49	7.5988	2.4061	754.8	NDNS
14807	Male	41	91.3	28.54	14.7	95	6.7463	2.0784	971.2	NDNS
14808	Male	44	92.75	29.54	14	226	6.7448	1.2184	413.2	NDNS
14810	Male	35	69.55	23.33	14.3	134	6.8718	2.8834	548.6	NDNS
14816	Male	24	77	26.21	15.7	58	10.4826	0.652	1230.2	NDNS
14818	Male	28	101.9	29.87	15.5	73	13.2659	2.9919	1489.7	NDNS
14823	Male	21	61.6	18.46	13	93	16.046	4.006	1048.9	NDNS
14834	Male	31	96.4	29.9	15.6	70	11.8156	3.5406	836.4	NDNS
14910	Female	29	67.2	23.78	12.8	42	11.787	1.457	984.6	NDNS
14913	Female	34	78.6	32.63	13.1	29	10.5503	1.1667	799.6	NDNS
14914	Male	57	91.8	34.94	14	51	18.538	6.6211	1214.9	NDNS
14918	Female	57	59.45	21.21	13.2	63	11.9341	1.3626	769.5	NDNS
14922	Female	37	74.05	28.23	13.3	11	5.716	0.3516	1169.1	NDNS
14932	Female	44	81.2	30.38	10.8	9	9.7244	1.0463	1074.5	NDNS
15014	Male	33	90.35	30.43	16.2	235	17.8786	2.8743	1442	NDNS
15016	Female	31	76.35	30.11	15	80	6.6402	2.2692	546.1	NDNS
15020	Male	34	69.55	26.7	15.2	93	6.5178	2.5395	410.3	NDNS

15027	Male	50	68.15	21.19	17.3	214	11.4548	3.7864	673.7	NDNS
15107	Male	35	95.85	29.96	14.8	103	14.8144	2.5496	1376.5	NDNS
15108	Male	32	67.8	22.97	15.5	64	7.7138	1.8018	858.1	NDNS
15110	Female	59	78.1	30.99	13.4	161	5.1319	0.5901	724.1	NDNS
15112	Male	30	97.45	31.8	15.2	45	22.2299	3.1823	2284.6	NDNS
15113	Female	32	86.7	30.04	14.6	57	8.8613	0.9634	1095.9	NDNS
15114	Male	48	83.35	28.62	15.4	121	10.5949	2.2757	756.8	NDNS
15117	Male	35	94.45	30.16	14.2	103	14.9562	3.0481	1975.5	NDNS
15118	Female	53	68.7	25.36	14.1	64	13.8557	3.6976	864.2	NDNS
15121	Male	51	81.2	27.26	15.3	38	22.3326	1.9395	1455.8	NDNS
15127	Male	49	65	20.39	15.5	48	19.7524	0.8748	1327.3	NDNS
15130	Female	53	52	20.62	15.7	126	8.4291	1.8502	938.1	NDNS
15131	Male	40	84.1	25.22	14.4	105	16.5328	3.9375	922.6	NDNS
15133	Male	53	83.7	30.73	13.5	84	9.4511	1.2667	1108.6	NDNS
15136	Male	50	85.7	27.39	14.8	49	16.1701	0	865.7	NDNS
15204	Male	64	72.05	25.39	15	124	17.5459	1.2745	1088.5	NDNS
15208	Male	56	76.1	24.95	15.4	57	15.6065	3.4828	999	NDNS
15211	Female	45	86.15	28.83	13.7	12	7.6109	2.4386	967.4	NDNS
15213	Female	22	81.35	26.69	13	29	7.362	2.609	586.3	NDNS
15226	Female	52	58.35	23.05	12.8	21	7.3095	0.3735	936.8	NDNS
15302	Female	31	66.7	26.38	15.3	23	5.5669	1.1436	609.9	NDNS
15303	Female	24	83.65	28.46	14.5	65	17.9642	3.0446	992.4	NDNS
15306	Female	62	53.6	22.2	13.8	93	5.9406	1.0002	696.5	NDNS
15312	Female	36	63.5	23.3	14.1	43	11.0315	1.7634	590.5	NDNS
15317	Male	31	84.1	24.57	15.5	117	9.5231	3.5711	1045	NDNS
15318	Male	21	89.55	30.13	15	179	14.6653	5.522	806.3	NDNS
15332	Female	35	60.9	22.87	13.4	15	12.6154	1.7931	863.3	NDNS
15336	Female	33	74.1	26.67	14.5	95	4.0195	2.2304	362.9	NDNS
15404	Male	27	84.65	25.95	14.4	89	13.6343	4.0564	1001.3	NDNS
15405	Female	31	55	21.96	11.8	25	13.5076	0	953.1	NDNS
15407	Male	36	81	24.29	13.7	87	16.0042	2.36	1074.5	NDNS
15415	Female	43	58.1	21.83	12.5	15	12.4501	1.8481	970.9	NDNS
15421	Male	29	84.9	24.37	16.1	68	19.8474	1.4246	872.6	NDNS
15422	Male	52	73.85	24.83	13.8	141	18.6414	2.5559	1197	NDNS
15423	Male	35	64.75	21	14.7	53	14.269	2.7136	1670.3	NDNS
15501	Male	34	61.6	17.63	15.6	88	10.126	1.8424	684	NDNS
15502	Female	46	56.4	21.5	10	13	9.0494	2.588	996.8	NDNS
15504	Male	50	107	31.4	15.6	148	13.6068	4.0385	855.2	NDNS
15514	Male	31	94.9	33.62	14.7	30	10.5521	2.4603	514.9	NDNS
15515	Male	44	78.4	28.8	16.1	162	12.9403	2.3947	1199.7	NDNS
15518	Male	38	89.7	27.65	15.4	162	20.1535	6.9569	1730.8	NDNS
15533	Male	45	90.3	26.47	13.8	134	15.0355	5.7787	983.5	NDNS
15601	Female	40	60.5	22.98	12.7	18	10.9803	0.5303	805.1	NDNS
15602	Female	56	57.9	24.07	12.8	46	8.9614	0.6948	911.9	NDNS
15616	Male	36	67.5	23.89	12.7	62	9.527	3.3215	1130.3	NDNS
15621	Female	31	54.85	19.19	11.9	65	9.5467	1.8363	798.1	NDNS
15632	Female	60	68.35	23.14	13.9	106	12.1303	1.2137	981.3	NDNS
15638	Female	20	57.85	19.92	11.6	19	9.9023	1.7315	673.9	NDNS
15640	Female	34	71.8	27.79	11.9	5	7.9259	0	750.3	NDNS
15709	Male	30	90.45	26.44	15.4	122	13.6763	3.2862	851.1	NDNS
15710	Male	23	63.4	20.61	14.1	77	10.4097	2.4651	895.5	NDNS
15720	Male	39	86.25	26.3	14.5	82	17.2875	1.7217	1201.1	NDNS
15728	Male	25	103.25	32.1	16.7	190	12.4942	1.9469	934.5	NDNS
15815	Male	27	99.5	30.01	14.7	49	7.062	1.1849	707.9	NDNS
15816	Male	31	80.65	25.37	16.1	39	16.5803	2.5896	1094.8	NDNS
15821	Female	36	56.7	24.05	12.9	69	5.7455	1.2136	773.5	NDNS
15824	Female	60	60.5	25.88	13.6	96	8.1297	1.2467	443.8	NDNS
15827	Male	57	95.5	32.04		133	13.3119	1.9025	1006.3	NDNS

15917	Male	56	74.55	23.73	14.8	162	12.2718	0.2349	990.5 NDNS
15938	Female	35	47.1	20.29	13.5	35	10.1746	1.1778	614.1 NDNS
15940	Male	35	90.2	29.59	15.3	50	6.6614	0.7438	808.3 NDNS
16007	Female	45	83.2	27.11	11.5	14	11.4154	1.9252	1173.7 NDNS
16013	Male	45	81.9	24.39	14.1	49	16.3005	1.8738	1608.2 NDNS
16015	Female	43	66.3	25.55	12	18	10.9601	2.3301	833.4 NDNS
16017	Male	24	77.4	23.84	14.5	17	9.5075	1.9632	963.7 NDNS
16021	Female	29	62.3	23.53	14	25	5.2958	1.758	319.7 NDNS
16037	Female	63	69.2	27.83	13.7	126	9.2927	1.7163	573.2 NDNS
16040	Male	40	91.8	27.94	14.8	118	9.5193	1.8051	783.2 NDNS
16113	Female	29	65.1	25.59	11.8	16	12.0092	0.3862	498.5 NDNS
16213	Female	24	56.15	21.04	12.9	76	9.2235	0.446	989.6 NDNS
16224	Male	54	98.7	35.52	14.3	235	3.5023	0.89	263.4 NDNS
16301	Male	24	89.65	26.96	16.1	178	14.1597	2.3709	1272 NDNS
16303	Female	26	75.7	26.76	12.2	17	5.4122	0.9236	671 NDNS
16309	Male	51	104.2	32.67	14.2	111	21.9034	7.4459	1604.7 NDNS
16311	Female	36	58.45	21.28	14.3	31	14.5331	1.5262	1306 NDNS
16313	Female	45	76.65	31.74	14.4	56	8.7223	0.5394	582.2 NDNS
16319	Male	29	81.4	29.4	13.2	141	7.8782	3.3979	342.1 NDNS
16327	Female	32	73.75	29.88	13.2	14	7.8719	1.2387	963.7 NDNS
16329	Female	29	56.25	20.45	13	26	9.0022	1.0427	823.3 NDNS
16332	Female	60	62.45	26.08	13.2	29	14.9338	0.5631	1428.8 NDNS
16333	Female	42	79.15	29.21	12.4	31	7.096	2.4617	783.8 NDNS
16334	Female	23	78.2	29.18	14	83	12.4512	1.2347	913.3 NDNS
16414	Male	49	79.4	27.98	14.5	297	13.0088	2.3447	727.6 NDNS
16423	Female	36	66.2	25.26	14.2	22	4.7641	0.721	652.5 NDNS
16424	Female	36	47.7	17.92	10.6	10	5.6323	1.218	421.1 NDNS
16425	Female	42	84.2	32.28	15.4	36	10.7259	0.4214	1217.9 NDNS
16427	Female	35	62.1	26.07	13.5	88	10.9393	1.2268	759.8 NDNS
16428	Male	39	89.2	29.19	15.3	61	12.1043	2.1816	717.9 NDNS
16501	Female	50	51.8	21.63	12	86	9.2306	2.7431	894.4 NDNS
16503	Female	58	88.05	35	14.4	214	20.8951	1.075	1127.9 NDNS
16517	Female	64	72	27.32	13.1	77	10.1063	0.8892	1020.5 NDNS
16518	Female	20	52.3	18.16	12.2	43	7.4359	1.8925	755.9 NDNS
16519	Female	50	68.7	30.29	12.7	36	4.0467	0.514	561.2 NDNS
16521	Female	36	96.55	41.68	12.4	50	8.8319	2.1537	624.6 NDNS
16603	Female	60	69	23.53	15.8	161	12.3723	2.3514	852.6 NDNS
16604	Male	32	70.75	21.32	14.6	78	15.9246	2.1077	1149.2 NDNS
16605	Female	64	65.1	26.55	15.7	22	7.9232	1.1698	679.9 NDNS
16606	Male	53	89.45	27.95	14	164	13.6978	3.5214	918.3 NDNS
16615	Male	37	76.4	27.18	16.8	241	17.1221	1.4885	1068.5 NDNS
16626	Male	34	76.05	23.88	14	186	6.9212	1.8694	754.8 NDNS
16630	Male	22	77.15	21.84	15.5	109	8.1384	1.733	1010.9 NDNS
16706	Female	62	72.3	30.47	13.6	102	9.9133	2.6762	559.5 NDNS
16708	Male	20	82.35	24.93	15.2	28	14.2029	1.3789	1234.8 NDNS
16712	Female	57	58.7	20.24	13.9	27	14.3367	0	964.3 NDNS
16715	Female	42	73.55	29.41	13.9	16	10.0308	1.9855	802.1 NDNS
16718	Female	29	61.45	25.45	13.2	29	19.5593	1.6182	612.1 NDNS
16730	Male	46	131.4	34.38	16.3	350	18.5901	2.2056	1996.5 NDNS
16732	Female	41	68.4	27.07	14.1	17	11.4701	1.2618	996.2 NDNS
16735	Female	19	53.35	19.34	13.4	30	15.4712	0.3661	848.1 NDNS
16804	Female	40	65.3	24.09	14.3	17	12.8036	1.6402	810.7 NDNS
16807	Female	42	91.3	38.32	14.4	39	4.3174	1.3774	346.5 NDNS
16808	Male	55	78.4	24.83	13.1	12	14.7724	0.8622	1368.7 NDNS
16811	Female	23	70.3	25.3	12.1	26	5.9004	0.6905	668.6 NDNS
16907	Female	48	70.4	26.22	12.6	31	17.0328	1.2519	936.3 NDNS
16910	Female	34	72.15	25.43	13.4	67	8.2834	0.8398	850.1 NDNS
16913	Female	60	106.15	36.28	15.6	34	11.3475	1.6262	745.4 NDNS

16914	Male	43	76.4	24.95	15.9	41	16.215	0	1458.9	NDNS
16920	Female	52	61.3	21.59	11.8	85	12.0812	1.2137	796.4	NDNS
16924	Male	55	75.95	26.78	14.8	82	6.0704	0.7442	682.7	NDNS
16927	Male	50	68.05	19.21	13	32	15.6851	0.5531	1140.7	NDNS
16933	Female	63	78.35	27.32	11.9	82	12.6998	2.3172	909.7	NDNS
16936	Female	63	70.95	26.77	12.5	85	8.0961	0.2743	1226.3	NDNS
17002	Female	48	63.1	23.46	13	83	6.738	1.0626	1150.9	NDNS
17005	Male	38	74.35	26.19	14.5	71	21.7574	2.1292	1157.5	NDNS
17023	Male	46	93.4	28.64	15	159	10.281	0.901	1161.3	NDNS
17024	Male	53	98.1	29.36	15.4	96	22.7659	2.3895	881.6	NDNS
17025	Female	42	94	30.17	14.7	92	13.2353	2.8972	448.6	NDNS
17028	Female	30	58.25	27.9	11.7	48	10.9329	1.7094	669.1	NDNS
17029	Male	31	67.2	22.98	13.3	42	7.6403	1.9999	532.1	NDNS
17030	Male	55	79.25	26.13	13.7	132	16.1033	1.8446	1078.2	NDNS
17102	Female	44	58.3	23.18	14.1	66	6.9517	1.0553	429.2	NDNS
17104	Female	42	53.15	22.48	14.2	12	11.8802	0.7875	747.8	NDNS
17107	Female	55	69.9	28	11.6	14	8.702	0.8558	1002.7	NDNS
17109	Male	37	107.95	33.26	14.5	151	16.2043	3.6233	1215	NDNS
17110	Male	47	78.6	24.06	13.9	57	10.4811	0.7585	1156.3	NDNS
17114	Female	47	60.7	23.52	13.5	44	11.7053	1.2323	858.3	NDNS
17122	Male	64	82.2	28.71	15.5	65	13.5373	1.6417	824.5	NDNS
17125	Female	38	64.35	26.61	14.2	145	11.1714	1.4007	818	NDNS
17126	Male	38	84.35	26.58	15.1	84	28.3633	4.8544	1708.9	NDNS
17128	Female	45	50.4	22.08	12.9	65	11.7576	1.2875	1099.1	NDNS
17133	Male	27	73.5	22.29	16.9	77	9.0585	3.0348	888	NDNS
17135	Female	61	63.35	23.47	12.1	60	12.6831	2.4901	1783.8	NDNS
17136	Female	37	70.2	25.79	11.7	66	13.2028	1.8659	812.1	NDNS
17137	Female	34	84.3	30.35	13.9	13	8.3384	1.0667	595	NDNS
17207	Male	50	100.9	31.99	15.8	160	19.681	8.6017	1105.4	NDNS
17208	Male	36	118.55	35.63	15.3	84	10.5943	2.5905	438	NDNS
17216	Female	45	59	23.16	12.3	13	14.8527	0	930	NDNS
17224	Male	61	81.15	25.73	15.1	171	13.7122	4.0043	1118.6	NDNS
17231	Female	31	61.05	22.73	13.1	42	17.0889	2.353	686.9	NDNS
17237	Male	63	92.85	27.57	15.3	306	16.2212	1.3912	1643.2	NDNS
17318	Female	62	51.65	19.66	13.6	78	10.6534	2.4772	1345.6	NDNS
17338	Male	36	109.25	37.3	15.3	77	11.0157	2.9629	764.9	NDNS
17401	Female	34	84.9	33.54	14.4	108	9.5274	0	756.5	NDNS
17402	Female	37	71.35	28.22	13.8	45	9.5441	1.3868	666.6	NDNS
17403	Female	29	66.25	27.92	13.5	44	11.1532	2.6838	619.4	NDNS
17408	Female	53	63.6	27.97	12.1	158	9.8626	1.8656	596.8	NDNS
17409	Female	56	51.85	23.17	14.1	55	16.5463	2.9456	952.5	NDNS
17410	Female	40	94.5	37.26	12.7	151	7.4308	2.7644	1042.5	NDNS
17414	Female	21	72.9	26.97	13.2	35	5.7794	2.3787	347.1	NDNS
17416	Male	48	84.8	27.82	14.9	129	9.3675	3.245	516.5	NDNS
17417	Male	27	76.9	26.91	14.8	153	12.6311	2.4899	880.4	NDNS
17418	Female	59	53.4	21.1	11.8	84	6.1182	1.3057	410.2	NDNS
17424	Female	64	61.6	23.23	12.5	132	11.4098	2.6455	831.5	NDNS
17427	Female	33	68.3	25.33	12.7	39	16.5083	3.2879	755.3	NDNS
17514	Female	36	64.1	23.44	12.6	18	11.2309	1.057	799.2	NDNS
17517	Male	32	84.9	26.38	15.1	133	10.7259	0.362	1250.2	NDNS
17522	Female	53	70.7	25.63	12.7	94	11.1521	1.4956	1027.5	NDNS
17524	Female	48	63.9	26.53	13	30	15.4424	0.1085	706.9	NDNS
17527	Male	39	78.9	24.11	15.5	163	10.3743	1.4975	1146.3	NDNS
17531	Male	34	75.9	24.34	15	27	13.0823	2.5854	992.5	NDNS
17535	Female	23	68.6	24.48	13.1	51	7.4095	0.6471	631.9	NDNS
17602	Male	53	91.2	30.99	13.7	54	8.3745	1.1552	1278.8	NDNS
17608	Male	30	97.3	29.47	15.1	154	17.3863	1.0782	1057.1	NDNS
17610	Male	39	68	24.86	17	45	12.9646	2.8214	1083.3	NDNS

17612	Female	30	69.7	25.6	14.1	94	9.6952	2.7234	559.8	NDNS
17615	Male	35	97.55	31.15	15.1	121	4.6117	0.4228	590	NDNS
17631	Male	30	72.5	24.68	14.6	48	8.8158	3.8128	682.9	NDNS
17633	Male	25	65	21.43	15.2	97	11.8699	4.2877	598.8	NDNS
17639	Male	56	79.2	26.86	16	13	18.0329	4.2495	1217.4	NDNS
17701	Male	53	71	23.08	13.3	18	7.4557	2.5113	867	NDNS
17702	Female	35	60.1	24.79	12.2	18	6.6199	1.5725	461.3	NDNS
17707	Male	32	103.2	30.77	14.7	116	6.7315	1.6237	559.7	NDNS
17708	Female	30	54.2	23.44	13.2	17	5.5035	1.897	866.2	NDNS
17709	Female	52	72.2	29.25	12.4	61	12.0425	2.1138	1088.7	NDNS
17711	Male	27	71.4	23.27	15.3	74	7.5327	1.824	856.1	NDNS
17712	Male	37	81.6	29.54	13.6	88	9.159	4.9653	614.5	NDNS
17717	Female	21	81.9	27.88	12.8	16	4.3817	1.6689	444.7	NDNS
17719	Male	34	82	30.16	17.2	43	22.086	1.905	1123.6	NDNS
17732	Female	64	63.35	28.71	14.6	136	11.5085	0.8804	980	NDNS
17811	Female	32	63.4	24.66	11.3	18	11.7097	0	1028.4	NDNS
17812	Male	52	88.2	28.59	14.5	115	11.5539	3.5321	921.9	NDNS
17815	Female	52	65.65	26.57	13.2	16	7.8352	1.2711	463.2	NDNS
17822	Male	31	56.7	19.17	13	51	7.4321	2.1314	687.6	NDNS
17823	Male	19	87.1	25.46	14.7	67	22.284	1.4914	1138.4	NDNS
17826	Female	36	63.5	21.61	11.5	32	13.5366	0	1005.1	NDNS
17906	Male	31	60.6	18.88	15.6	74	15.8425	1.0668	3993.9	NDNS
17910	Female	50	76.2	28.72	13.6	97	10.3884	3.1156	669.6	NDNS
17916	Female	34	73.5	28.43	13.2	14	7.1108	0.3573	506.4	NDNS
17921	Female	28	64.6	22.77	12.8	71	5.0738	0.5529	489.9	NDNS
17938	Female	36	113.7	38.5	12.9	83	3.8062	1.2363	390.4	NDNS
17939	Female	39	66.9	26.63	13.6	19	9.1282	3.0415	859.4	NDNS
18003	Female	51	75	29.7	14.3	68	10.9746	0	830	NDNS
18007	Female	52	97.6	35.33	10.8	5	12.8868	1.6045	746.7	NDNS
18009	Male	40	68.75	24.77	16.8	44	16.0528	0	754.6	NDNS
18010	Male	63	65.2	28.24	13.2	42	5.7564	0	498.4	NDNS
18013	Female	60	69.4	31.79	13.4	63	7.0074	0.8716	499	NDNS
18021	Male	60	87.5	32.04	15.2	36	15.4611	3.9559	1641.9	NDNS
18039	Female	36	62.45	25.68	11.7	34	8.4348	1.18	738.4	NDNS
18118	Male	23	74.3	23.45	17.2	72	9.7774	3.3889	749.5	NDNS
18122	Male	29	74.4	25.59	16	137	14.3581	5.7852	312.3	NDNS
18131	Female	48	102.5	34.89	13.2	55	29.0939	1.3822	1106.8	NDNS
18201	Male	53	89.6	30.52	13.8	180	10.0948	0.6298	952.1	NDNS
18203	Female	44	64.8	26.17	11.2	12	20.3406	6.7357	1080.7	NDNS
18205	Female	39	52.4	18.92	14.8	57	9.3952	5.4637	1078.3	NDNS
18210	Male	33	76.9	26.64	13.5	164	7.3125	2.0109	401.6	NDNS
18212	Male	30	72	23.32	13.1	198	8.3881	2.6183	337.9	NDNS
18215	Male	29	73.2	25.21	16.3	144	20.2007	3.426	1095.6	NDNS
18321	Male	57	81	23.29	14.8	195	11.855	0	679.2	NDNS
18409	Male	30	91.4	26.31	15.4	90	15.2221	2.284	1280	NDNS
18414	Male	45	79.4	23.87	14.8	84	10.4798	1.5189	1021.2	NDNS
18415	Female	58	86.8	31.67	12.9	166	9.6417	0.7305	1063.1	NDNS
18417	Female	33	57.15	22.34	12.6	72	7.95	0.4716	820.2	NDNS
18418	Male	28	84.1	23.85	14.4	52	13.0529	1.8823	1143	NDNS
18422	Female	59	62.65	22.13	13.4	77	9.7289	3.3295	486.7	NDNS
18430	Male	32	98.65	27.41	15.5	51	9.5232	0.967	645.1	NDNS
18433	Male	48	74.35	25.12	15.5	64	18.8902	6.623	962.2	NDNS
18436	Male	35	82.15	23.66	14.6	121	12.1205	2.5364	692.7	NDNS
18440	Female	58	67	24.18	13.4	51	17.3661	0.9055	795.4	NDNS
18502	Male	36	71.2	26.98	15.2	214	10.6457	1.7657	1014.2	NDNS
18505	Male	37	80.9	24.04	15.8	88	27.4731	0.8682	1349.3	NDNS
18508	Male	49	85.3	29.36	14.9	239	10.7746	3.413	370.2	NDNS
18510	Female	42	42.6	17.3	13	60	13.4103	1.2941	867.7	NDNS

18512	Male	35	99.55	31.42	16	256	13.4775	6.4743	1059.1	NDNS
18513	Female	32	87.65	34.67	12.8	67	6.8914	2.8096	449	NDNS
18515	Male	26	74	25.41	13.7	67	13.7654	2.805	722.1	NDNS
18519	Male	24	55.8	17.57	13.1	33	21.2306	0	1294.3	NDNS
18522	Male	46	79.75	23.92	14.3	101	14.2781	1.7625	1016.5	NDNS
18523	Female	37	73.6	29.15	12.3	38	11.9871	0.9794	799.6	NDNS
18526	Female	60	67.7	30.35	12.7	72	9.7011	1.2902	532.1	NDNS
18527	Male	59	79.4	24.84	16.8	225	9.9246	4.7377	623	NDNS
18533	Male	35	66.1	22.45	12.2	13	15.718	2.6036	973.4	NDNS
18536	Male	41	102.25	27.87	15.9	150	26.5425	2.3085	1448.1	NDNS
18537	Female	38	59.1	25.6	13.7	40	8.1058	1.6403	326.5	NDNS
18604	Male	53	101.4	37.45	17.7	105	12.5762	0.7842	602.1	NDNS
18607	Female	56	74	28.02	13	47	8.2347	1.1846	445.8	NDNS
18713	Female	35	62.9	24.15	13.8	29	9.3405	0.9327	758.5	NDNS
18735	Female	50	89.25	35.24	13	88	22.3632	2.1288	1176.9	NDNS
18827	Female	46	99.3	44.43	10.6	21	11.0895	2.2006	953.4	NDNS
18829	Female	47	61.8	22.19	13.7	330	8.8454	1.8448	500.1	NDNS
18904	Female	27	51.1	19.39	12.7	33	7.7388	0.8517	572.4	NDNS
18907	Female	57	71.8	26.79	15.2	58	8.5406	0.5744	841.4	NDNS
18911	Female	40	66.3	23.63	14.1	24	11.0667	2.2158	641.4	NDNS
19007	Female	53	98.7	35.73	12.6	35	9.6152	0	534.8	NDNS
19009	Male	35	94.05	32.07	15.9	407	16.4553	2.8217	984.9	NDNS
19015	Male	27	82.3	26.23	15.2	41	13.7359	3.2018	1116.2	NDNS
19020	Male	34	88.9	27.39	15.7	65	16.0741	2.8584	1263	NDNS
19026	Female	41	62.6	23.75		32	13.8541	3.221	668.4	NDNS
19103	Female	54	66.85	24.07	16.7	212	9.9878	1.3586	391	NDNS
19104	Male	50	87.5	26.29	14.7	166	5.3347	0.373	550.7	NDNS
19107	Female	64	57.6	25.5	13.4	141	3.7121	0.1457	605.8	NDNS
19108	Female	60	72.2	29.63	14.3	150	10.9311	0.0908	476.4	NDNS
19110	Female	53	59.4	23.45	14.8	12	9.8723	0	684	NDNS
19126	Female	49	54	21.69	12.4	6	9.7518	1.4477	694.3	NDNS
19135	Female	55	46.5	16.47	10.4	5	10.1402	1.3869	968.4	NDNS
19206	Male	47	87.95	30.36	14.5	202	18.0936	7.3047	1308	NDNS
19222	Male	53	87.4	25.96	15	101	16.1435	4.4218	493.9	NDNS
19227	Female	49	78.55	31.51	13.6	118	10.8972	0.3485	808.1	NDNS
19231	Male	33	98.4	28.06	15.3	18	13.0419	4.1728	949.4	NDNS
19235	Female	24	53.15	19.83	14.9	29	9.5032	1.6436	855	NDNS
19311	Female	32	93.75	34.64	13.9	78	12.0166	1.2276	722.3	NDNS
19336	Female	41	81.5	33.43	14.8	105	7.8583	2.3262	530.3	NDNS
19338	Female	32	54.3	20.41	13.4	79	4.7274	0.5171	356.5	NDNS
19407	Female	56	77.3	32.81	13.3	49	7.4603	0.5523	1044.2	NDNS
19409	Female	38	70.8	28.83	13.2	31	8.0429	2.0959	324.9	NDNS
19410	Female	20	46.3	18.36	10.8	15	10.7231	0	1299.6	NDNS
19502	Male	41	109.75	32.84	14.2	113	18.8687	1.6987	919.4	NDNS
19507	Female	35	64.5	24.35	14.2	33	10.3483	0	611.4	NDNS
19516	Male	48	84.85	27.66	15.2	22	11.1083	0.891	885.1	NDNS
19522	Female	30	63.4	23.62	12.1	32	15.0977	0.0677	534.8	NDNS
19529	Male	35	116.9	31.22	15.5	105	12.4775	2.5418	1168.6	NDNS
19538	Female	38	81	30.02	13.7	31	17.3003	0	1176.6	NDNS
19604	Male	30	61.15	19.74	13.1	166	14.3167	2.0798	814.4	NDNS
19605	Female	38	53.1	22.07	13.3	5	14.7324	0	1052.9	NDNS
19611	Male	20	95.3	27.88	13.8	85	8.9146	5.2377	431.6	NDNS
19614	Female	39	94.25	38.14	13	23	6.3079	1.627	652.6	NDNS
19616	Male	62	77.35	27.5	13	134	10.0258	0.756	830.8	NDNS
19617	Male	41	75.95	26.02	16.4	55	22.3395	0.572	1304.5	NDNS
19622	Male	47	79.3	25.14	14	187	15.4044	0.3442	1210.3	NDNS
19626	Female	54	65.7	24	12.5	44	16.918	0	689.8	NDNS
19627	Female	33	65	26.9	13.1	35	9.805	0.594	815.3	NDNS

19632	Female	26	47.05	21.42	13.7	33	6.2746	0	719.4	NDNS
19633	Male	36	65.6	26.58	16.2	73	17.0675	3.0229	409.3	NDNS
19707	Male	42	91.1	29.09	15.6	211	9.5926	1.3657	1047.4	NDNS
19710	Female	33	61.3	22.34	15	23	14.8566	1.8231	718.3	NDNS
19713	Female	37	71.3	26.57	14.4	132	12.3371	2.962	619.3	NDNS
19717	Male	46	75.35	21.76	15.7	56	12.0289	0.7774	1228.8	NDNS
19718	Male	20	56.9	19.3		60	4.8187	1.7276	263.4	NDNS
19719	Female	37	76.8	30.63	13	38	3.5247	0.2102	897.9	NDNS
19727	Female	64	70.9	25.58	13	48	10.4716	1.8494	981.4	NDNS
19729	Female	47	58.5	21.53	14	46	7.1018	2.6364	286	NDNS
19737	Female	25	82.4	30.45	13.3	90	10.5877	0.9767	1317.9	NDNS
19739	Male	57	73	26.14	15.9	42	12.4784	1.7817	1145.9	NDNS
19801	Male	54	99.4	28.49	15.3	138	12.3	2.5583	849	NDNS
19806	Male	25	91.15	29.75	15	109	8.8965	5.9692	901.7	NDNS
19810	Male	19	109.05	33.6	16.6	138	14.5247	1.5712	1793.8	NDNS
19815	Female	62	70.35	25.06	12.3	100	11.7367	2.8559	763.4	NDNS
19816	Male	52	109.2	35.7	15.8	383	17.8618	3.3152	1258.4	NDNS
19825	Male	36	58.6	23.18	9.5	686	16.7379	2.951	1851.1	NDNS
20028	Female	59	86.75	29.65	12.9	162	9.8084	0.5858	1133	NDNS
20033	Male	42	86.65	30.61	16.4	65	10.8212	2.6945	677.5	NDNS
20102	Female	31	62.85	23.95	14.7	58	11.0775	1.5936	783.4	NDNS
20103	Male	46	95.75	29.15	15	150	14.8509	2.6583	1427.6	NDNS
20105	Male	59	111	29.45	14.7	29	16.0448	1.9239	1896.8	NDNS
20114	Female	26	46.7	18.4	13.3	51	9.1715	2.8614	567.4	NDNS
20117	Female	39	70.9	27.52	13.7	19	4.0751	0	286.7	NDNS
20119	Female	33	80.05	29.47	14.8	20	8.9919	3.4865	476.8	NDNS
20122	Female	32	71.4	26.29	14.3	25	8.7923	1.8748	815.7	NDNS
20134	Female	20	55.1	20.59	13.6	21	6.8649	0	480.1	NDNS
20136	Female	20	54.8	20.25	14.9	23	6.3313	3.3538	911.1	NDNS
20140	Female	60	70.3	26.99	12.2	39	21.5206	0.2843	1451.2	NDNS
20214	Female	37	49.1	20.36	13.8	38	7.4019	0.5716	738.2	NDNS
20228	Female	20	48.35	18.89	13.5	43	8.1356	0.9391	502.2	NDNS
20307	Male	20	92.35	29.43	15.2	83	11.2259	3.435	623.2	NDNS
20308	Female	62	49.6	19.98	14	15	8.391	2.2798	575.1	NDNS
20309	Female	41	64.75	24.12	11.1	8	13.0281	1.4311	1019.9	NDNS
20311	Male	28	78.7	27.17	14.7	131	14.5381	0.4155	906.4	NDNS
20313	Female	54	69.5	24.86	15.2	90	10.0797	2.0102	612.1	NDNS
20318	Male	30	78.9	26.27	12.7	22	21.8347	2.7438	1070.2	NDNS
20320	Female	36	55	22.2	13.3	134	2.5443	0.495	255.4	NDNS
20322	Female	37	60.7	22.54	12.6	30	12.3628	1.1897	907.6	NDNS
20325	Male	53	95.25	29.89	15.7	101	6.4844	1.3263	952.3	NDNS
20327	Female	44	53.15	23.83	12.9	20	12.7968	0.2864	698.8	NDNS
20330	Female	38	91	34.19	13.2	69	10.1401	1.4803	1061.6	NDNS
20333	Male	58	80.65	24.88	14.4	188	15.9695	3.239	1130.9	NDNS
20339	Female	43	53.55	20.07	13.1	25	14.7171	3.7667	1073.4	NDNS
20403	Female	34	57.45	22.95	14.6	32	9.8836	1.6291	783.2	NDNS
20405	Female	57	61.5	22.16	13.2	90	14.6244	0.6032	853	NDNS
20412	Male	28	70.5	21.76	15.4	173	8.2027	0.9797	633.2	NDNS
20416	Female	56	68.9	22.97	15.2	38	12.2215	0.1452	1450.4	NDNS
20420	Male	37	81.2	25.29	15.2	218	12.495	2.1704	815.6	NDNS
20426	Male	56	100.3	33.19	14.2	104	15.1254	1.9872	1194.6	NDNS
20427	Male	64	77.05	23.54	14.1	79	11.4303	1.02	1166.8	NDNS
20429	Male	64	76.8	24.85	13.8	193	12.3563	1.6595	861.1	NDNS
20438	Male	39	96.85	30.19	16.2	90	21.5608	3.8108	1772.3	NDNS
20508	Female	53	72.7	25.27	13.4	22	17.1362	2.5322	1207	NDNS
20509	Male	63	76.6	23.54	16.8	90	11.6594	0	1324	NDNS
20520	Male	41	70.35	22.84	16.4	156	11.9534	1.2125	1137.1	NDNS
20521	Male	47	73.5	23.69	14.5	91	13.4585	1.3097	958.2	NDNS

20530	Male	47	81.55	28.42	14.9	132	14.3531	3.5153	901.9	NDNS
20536	Male	43	68.4	18.71	14.7	113	6.1248	2.1309	448.9	NDNS
20604	Male	49	83.35	26.95	15.2	200	11.5625	3.4359	1027.6	NDNS
20612	Female	56	55.7	23.84	13.3	96	12.3156	1.7959	993.8	NDNS
20619	Female	44	63.6	23.53	13.1	49	13.9261	3.1379	1510.2	NDNS
20621	Female	58	67.7	24.39	12.2	23	15.6861	0.0531	830.1	NDNS
20624	Male	42	69.2	25.26	15.5	84	8.8541	0.8016	1319.1	NDNS
20626	Male	41	91.6	26.65	16.6	355	12.1038	3.4135	538.2	NDNS
20629	Male	39	84.8	26.82	15.1	82	19.4697	2.1406	1445.5	NDNS
20630	Male	36	74.85	22.72	16.7	127	10.9146	3.1604	948	NDNS
20702	Female	53	58.7	22.83	13.6	40	15.6658	1.5817	946.1	NDNS
20704	Male	43	99.4	28.23	14.3	159	10.3166	1.7928	1008.7	NDNS
20708	Male	27	73.6	21.61	15.8	39	18.8559	1.0341	910.5	NDNS
20713	Female	63	58.8	26.15	15.7	61	6.0789	0.9726	837.4	NDNS
20714	Female	42	52.5	22.15	13.9	45	17.43	0.9265	515.4	NDNS
20716	Male	42	71.8	24.45	12.7	39	17.2507	1.45	1222	NDNS
20717	Male	59	74.7	25.53	14.2	204	13.3296	2.2821	630.4	NDNS
20725	Female	30	51.9	20.12	13.1	7	7.6827	0	538.5	NDNS
20730	Male	30	81.1	25.6	16.9	53	11.1213	2.1286	701.6	NDNS
20732	Female	58	60.95	25	17.4	97	11.8192	1.305	763.7	NDNS
20736	Male	56	74.8	26.79	14.3	174	8.3502	1.1272	938.9	NDNS
20738	Male	51	76.9	25.53	15.4	31	22.3998	1.3906	1094.3	NDNS
20740	Male	52	85.2	31.26	14.3	33	12.3444	5.0706	1383.5	NDNS
20802	Female	39	53.4	21.06	14.2	55	15.9272	0.3502	699.6	NDNS
20806	Female	57	97	38.61	14.6	77	10.2215	0.4461	930.3	NDNS
20833	Male	33	88.5	26.91	14.2	166	6.9934	2.7805	646.3	NDNS
20834	Male	51	83.1	27.96	14.5	100	14.5828	2.1662	1001.5	NDNS
20836	Male	48	94.5	27.81	16.1	17	8.2427	0.6223	858.8	NDNS
20839	Male	36	94.8	28.22	15.8	80	19.6047	0.8691	1017.3	NDNS
20911	Female	31	44.1	18.12	13	35	5.8748	1.5893	665.4	NDNS
20914	Male	42	68.75	22.02	15.2	158	14.7931	2.5236	1176.4	NDNS
20917	Male	53	93.85	28.5	14.8	82	14.2972	2.6477	1442.2	NDNS
20929	Female	38	69	26.21	12.8	41	12.3063	3.4727	1411.1	NDNS
20930	Male	31	87.8	26.92	14.4	152	8.1993	3.152	608.7	NDNS
20931	Male	60	86.8	25.68	14	163	11.9994	3.9681	889.2	NDNS
21003	Female	46	61.95	20.63	12.7	33	13.581	1.7632	936.6	NDNS
21009	Male	55	83.3	31.84	14	20	12.5578	1.8309	1495.8	NDNS
21019	Female	44	56.95	22.99	12.7	40	7.832	2.0738	566.7	NDNS
21107	Female	33	55.05	20.71	14.1	65	8.3226	0.4501	489.9	NDNS
21109	Male	29	71.95	24.69	15.3	170	24.4548	3.7431	1048.4	NDNS
21112	Female	29	61	25.47	12.9	26	7.8947	1.8749	664.9	NDNS
21115	Male	23	72.4	25.01	16.9	155	12.0453	2.3329	1375.1	NDNS
21122	Male	48	107	30.24	15.6	48	22.0768	8.1043	1689.4	NDNS
21123	Female	37	62.3	24.69	13.8	18	5.6467	0.6414	580	NDNS
21125	Female	32	73.65	24.68	13	78	16.2634	1.7349	911.4	NDNS
21130	Male	38	105.9	32.2	15.8	245	15.9305	0.3478	1427.4	NDNS
21136	Male	41	70.8	23.01	14.6	24	21.5858	1.0022	1596.1	NDNS
21137	Male	35	93	30.45	17.2	184	20.1478	1.4739	1273.7	NDNS
21140	Female	36	57.45	24.3	13.9	39	13.8785	0.1732	1032.5	NDNS
21201	Female	39	66.9	24.56	12.3	48	20.3201	3.4785	1167	NDNS
21211	Female	31	74.5	23.91	12.6	44	13.8708	0.6225	932.9	NDNS
21215	Male	27	61.7	19.11	14.9	64	14.4262	1.9255	1069.8	NDNS
21216	Female	27	57.8	22.63	12.3	50	9.4096	0.5204	899.3	NDNS
21217	Female	31	58.65	22.17	13.5	73	10.4774	0.9092	527.8	NDNS
21220	Female	39	64.5	24.88	13.3	112	8.3768	0.5289	800.1	NDNS
21231	Male	55	80.5	29.71	16.6	208	10.1702	1.9035	842.5	NDNS
21233	Female	35	54.1	21.09	14.3	24	7.1293	1.641	635.1	NDNS
21238	Female	26	69.45	23.83	14	54	10.5298	1.6775	753.7	NDNS

21302	Female	22	80.2	27	12.9	54	11.961	1.9279	1088.4	NDNS
21311	Male	41	90.9	31.56	16	220	12.5414	2.692	1570.9	NDNS
21322	Male	34	68.55	22.08	15.8	100	15.0151	2.5634	1175.7	NDNS
21325	Male	37	94.25	30.88	14.8	149	20.6054	9.6688	1481.4	NDNS
21329	Male	32	83.25	28.45	15.6	188	8.4305	3.9179	928.8	NDNS
21333	Male	38	72.2	21.56	15.3	72	13.4283	2.6739	983.3	NDNS
21334	Female	21	60	24.45	14.6	21	8.4517	2.5212	589.7	NDNS
21335	Male	49	79.15	26.93	16.3	211	10.826	2.6985	389.7	NDNS
21339	Male	22	67.5	21.27	14.5	54	12.1453	1.7272	843.3	NDNS
21406	Female	42	53.3	20.92	12.4	12	9.2055	0	1056.2	NDNS
21408	Male	40	70.2	25.35	15.9	81	6.4682	1.783	487.1	NDNS
21411	Male	47	94.65	27.31	15.4	82	22.4965	0.5152	952.7	NDNS
21419	Female	63	90.55	32.57	12	66	4.4995	0.6565	170.5	NDNS
21423	Female	55	74.6	24.35	13.2	122	11.974	1.3116	975	NDNS
21424	Male	64	78.05	25.5	15.7	279	16.0332	2.1318	713.2	NDNS
21426	Male	58	72.85	26.14	13.3	76	10.5075	2.1876	698.8	NDNS
21502	Female	56	88.8	38.33	12.2	95	13.2003	0.1756	855.8	NDNS
21509	Female	46	62.4	23.98	13.6	26	10.7868	1.4507	591.3	NDNS
21518	Male	39	67.3	22.5	15.2	82	9.7297	2.3803	1207.5	NDNS
21522	Male	20	92.5	32.2	15.8	78	13.205	3.5192	536	NDNS
21536	Female	62	67.8	24.01	13.2	54	6.7002	1.3467	413.8	NDNS
21537	Female	50	95.3	34.56	13.4	75	10.6132	1.2795	1027.5	NDNS
21601	Male	58	77	25.7	15.2	204	13.452	3.7973	1001.7	NDNS
21605	Male	51	87.7	28.1	16.2	42	2.7819	0.3785	355.9	NDNS
21612	Male	27	70.25	22.11	14	47	9.3846	3.154	955.2	NDNS
21615	Female	23	65.95	23.7	12.6	32	7.7608	3.3742	715.9	NDNS
21619	Female	28	48.75	17.61	13.5	25	9.5256	1.3302	756.8	NDNS
21624	Female	27	66.9	27.26	14.5	35	3.0471	0.5668	379.8	NDNS
21629	Male	55	112.75	35.13	16.5	98	11.5768	3.6893	964.3	NDNS
21631	Male	54	81.25	24.8	14.9	86	13.851	4.1149	765.3	NDNS
21638	Male	42	75.25	24.17	15.6	224	8.6377	3.0468	659.1	NDNS
21704	Male	52	101.25	31.55	14.5	21	17.0741	2.2008	1827.8	NDNS
21706	Male	55	77.4	24.1	12.6	144	13.5534	2.4368	1729.6	NDNS
21711	Female	35	83.2	32.91	14.1	121	6.8624	1.6818	728	NDNS
21712	Female	30	59.25	23.57	13.3	22	9.3881	1.8842	847	NDNS
21725	Female	39	73.35	27.9	13.9	60	11.5407	1.4749	659.3	NDNS
21727	Male	35	78.5	24.35	14.7	115	12.6544	5.5818	668.3	NDNS
21732	Female	33	67.55	24.65	12.7	18	11.1159	1.0433	1347.2	NDNS
21737	Male	47	83.2	27.83	17.3	183	12.2171	2.4725	639.9	NDNS
21802	Female	33	57.2	24.44	15.6	40	3.5804	1.6801	430.6	NDNS
21805	Male	36	77.6	22.92	15.4	156	15.5018	2.5254	1157.7	NDNS
21826	Male	48	85.85	28.31	15.6	57	7.9948	1.9334	778.6	NDNS
21902	Female	56	84.5	34.35	13.6	58	16.2739	1.5821	757.9	NDNS
21903	Female	46	73.2	25.74	13.3	62	15.5591	2.5031	1257.3	NDNS
21906	Female	53	85.8	29.51	12.6	9	13.0645	1.1689	1068.6	NDNS
21910	Male	34	70.6	21.99	14.5	90	14.2463	2.7879	1041.6	NDNS
21913	Male	20	63.3	20.56	15.7	105	5.5132	1.0444	654.3	NDNS
21916	Male	54	86.6	32.86	15	119	11.7223	2.4211	1142.7	NDNS
21919	Female	43	75.9	28.01	11.5	18	8.7754	0.8853	961.5	NDNS
21920	Female	33	87.5	35.77	12.3	20	9.5621	0.7339	779.6	NDNS
21925	Female	37	58.85	21.77	14.5	44	15.7745	2.8702	1003.6	NDNS
21926	Female	38	57.1	21.27	13.5	25	11.9706	2.4401	763.4	NDNS
21927	Female	28	91.7	34.75	13.7	41	9.2442	0.9645	461.9	NDNS
21928	Female	24	57.3	21.24	14.4	38	12.08	2.2835	874	NDNS
21930	Male	63	83.6	27.5	14.3	22	20.4558	1.2409	1458.1	NDNS
22001	Male	37	80.4	28.12	14.1	76	10.9842	1.8015	845.9	NDNS
22008	Male	36	133.7	36.25	15.1	588	13.3247	1.8444	1270.9	NDNS
22013	Female	33	85.6	34.44	13.1	11	10.5684	0	698.5	NDNS

22015	Male	54	57.65	21.05	15.3	26	8.1347	0.6542	853.5	NDNS
22017	Male	54	71.65	22.2	15.8	278	12.9248	4.5665	1641.8	NDNS
22028	Male	26	79.9	26.07	16.6	39	24.891	0	951.3	NDNS
22030	Female	29	66.25	24.75	15	28	8.7235	1.693	1108.9	NDNS
22033	Female	61	77.95	29.7	14.6	20	14.1006	0.3294	894.2	NDNS
22036	Male	51	81.3	25.3	15.9	159	14.4762	3.5391	1405.4	NDNS
22037	Male	47	69.7	24.05	16.3	52	3.8619	0.6511	424.3	NDNS
22101	Female	29	48	21.09	13.2	27	7.4717	1.1131	630.5	NDNS
22107	Female	31	44.4	19.73	13.9	149	5.9009	0.7552	729.3	NDNS
22108	Female	33	72.8	27.3	12.6	43	22.1084	1.7056	1185.5	NDNS
22109	Male	62	65	22.5	14.6	74	23.2436	0	1394.1	NDNS
22116	Male	64	67.6	22.57	14	106	7.8526	2.1223	475.8	NDNS
22126	Female	38	92.9	33.94	15.4	21	2.5396	0.9769	200.3	NDNS
22131	Male	42	100	30.71	16.5	144	13.4973	3.8268	1116.5	NDNS
22203	Female	60	71.45	26.28	13.2	71	8.6894	1.7816	601.5	NDNS
22205	Female	50	78.55	29.44	13.8	46	10.5018	2.6551	1092.1	NDNS
22207	Male	44	94.05	30.36	14.3	61	14.6864	2.7448	860	NDNS
22209	Male	30	78.65	24.41	14.2	47	10.1613	1.9791	838.9	NDNS
22210	Male	36	94	31.08	14.8	80	27.5251	4.6314	1611	NDNS
22212	Male	46	86.85	26.09	14.8	92	19.6042	1.1373	1302.7	NDNS
22215	Female	29	57.6	21.21	13.4	29	7.538	1.7561	794.1	NDNS
22229	Male	47	83.9	23.24	15.3	64	13.1399	7.6499	1452.3	NDNS
22301	Male	63	97.6	28.18	13.9	72	9.9443	3.8464	859.4	NDNS
22311	Female	41	65.9	22.25	12.2	66	11.5294	2.2654	581.3	NDNS
22325	Male	59	90.65	27.04	13.5	327	15.9529	1.7813	824.4	NDNS
22402	Female	43	61.5	24.13	13.1	37	11.0506	0.4891	869.5	NDNS
22406	Female	38	79	30.52	14.2	76	8.7361	1.9058	715.4	NDNS
22407	Male	30	82.7	25.97	16.3	27	11.5332	1.4658	1238.4	NDNS
22416	Female	35	54.4	21	13	52	9.2136	1.7482	595	NDNS
22420	Male	36	103	34.22	14.7	124	7.8871	1.8801	832.7	NDNS
22421	Female	39	59.3	23.47	12.3	14	14.9882	0.3524	976.1	NDNS
22425	Male	51	87.85	27.53	15	109	13.4006	2.7669	799.7	NDNS
22426	Male	40	127	30.23	14.9	111	8.6972	1.8257	1014	NDNS
22429	Male	58	84.5	26.14	15.2	81	14.3087	3.2348	1141.6	NDNS
22430	Male	36	89.1	30.36	16.8	136	13.632	2.9115	1013.4	NDNS
22431	Male	37	82.6	28.51	15.2	38	10.7247	3.3926	984.3	NDNS
22432	Female	46	52.95	21.13	12.1	27	7.5836	2.2497	704.8	NDNS
22438	Female	37	80.1	29.49	14.1	17	5.2229	0.7917	687.4	NDNS
22507	Female	26	50.8	19.59	13.1	35	6.7077	1.1011	741.5	NDNS
22514	Male	30	74.8	25.24	15.2	95	11.6342	3.4733	816.9	NDNS
22517	Male	57	102.4	31.89	16.7	158	19.4487	4.2356	1104.5	NDNS
22519	Female	45	63.4	26.85	12.1	18	7.9164	1.5718	543.4	NDNS
22521	Male	50	73.7	25.53	14.6	80	11.6839	2.2396	750.1	NDNS
22522	Male	53	73.4	21.47	16.3	56	16.1163	0.8711	1067.4	NDNS
22523	Female	40	71.3	27.27	13.5	47	10.0541	1.6561	661.7	NDNS
22526	Male	43	94.5	28.47	15.4	67	14.7152	1.3295	1580.1	NDNS
22527	Female	34	55.8	22.49	12.2	22	7.7064	1.6264	699	NDNS
22528	Male	36	71.9	26.35	17.7	255	7.9553	0.5114	704.9	NDNS
22533	Female	51	94.7	39.52	11.4	66	13.1985	2.7381	1368.2	NDNS
22536	Female	64	74.3	29.65	14.8	120	8.896	2.4291	1376.9	NDNS
22601	Male	50	71.6	24.34	14.5	138	9.1625	3.1306	847.8	NDNS
22605	Male	63	91.15	30.54	15.2	121	13.1805	2.2358	1016.5	NDNS
22615	Female	63	69	27.09	14	122	7.6088	0.489	993.7	NDNS
22617	Male	62	91.3	27.99	14.1	320	14.6261	3.0415	1129.2	NDNS
22620	Female	39	76.8	27.99	15.3	51	12.3788	0	922.2	NDNS
22625	Female	60	61.4	24.43	11.9	13	11.9703	1.3844	1478	NDNS
22629	Male	51	76.7	26.34	16.4	53	17.3526	2.9798	1609.8	NDNS
22706	Male	36	80.1	25.28	14.5	67	14.5524	2.3898	1157.4	NDNS

22714	Female	55	83.7	34.39	14.3	30	4.9836	0.2683	776.7	NDNS
22719	Male	44	83.5	26.83	13.7	53	14.3847	7.549	764.8	NDNS
22727	Female	44	97	35.67	13.3	18	16.6121	0	920.7	NDNS
22738	Female	61	87.2	39.54	12.8	85	6.6952	1.1536	701.3	NDNS
22739	Female	42	71.3	26.8	11.5	27	8.4318	1.6391	925.4	NDNS
22802	Male	25	75.2	23.06	14.4	47	19.0225	1.5761	1126.2	NDNS
22808	Male	24	91.8	28.99	15.5	118	12.263	4.4354	1072.4	NDNS
22813	Female	40	53.4	20.82	13.4	35	7.2356	0.6055	681	NDNS
22814	Male	41	145.9	46.1	14.1	19	15.0354	1.5663	1005.4	NDNS
22817	Male	61	96.6	35.63	14.5	220	6.6017	1.7189	329.6	NDNS
22818	Male	38	68.6	22.49	12.6	53	21.201	5.5134	1887.2	NDNS
22828	Female	63	54	21.47	14.1	64	10.9141	0.8254	912.7	NDNS
22829	Female	32	69.2	28	12.2	11	9.4239	1.7036	689.8	NDNS
22830	Female	45	74.6	28.27	12.8	23	3.7004	0	319.6	NDNS
22831	Male	44	67.1	20.58	14.8	43	13.1285	3.9055	1245.2	NDNS
22834	Male	54	92.1	32.46	14.9	104	10.3592	2.726	982.1	NDNS
22902	Female	39	75.85	27.81	14.1	34	19.6839	1.2403	1214.4	NDNS
22903	Male	23	61.1	20.67	14.1	84	5.7252	2.4937	511.8	NDNS
22905	Female	34	64.15	24.72	13.5	50	6.5545	1.3924	484.7	NDNS
22907	Male	36	80.35	23.2	15.7	192	12.7525	3.3357	1081.1	NDNS
22911	Female	40	91.95	32.37	12.7	54	10.7894	1.4432	911.1	NDNS
22913	Female	27	92.7	33.64	13.9	39	9.8126	1.978	718.1	NDNS
22923	Female	56	75.8	27.74	13.7	22	8.3439	0.0715	653.4	NDNS
22927	Female	37	68.4	25.98	13.4	40	8.6014	1.9078	1146.3	NDNS
22929	Female	20	90.7	35.56	14	42	9.9589	3.1052	595.5	NDNS
22934	Female	33	61.3	23.87	11.5	10	5.694	0.2708	865.6	NDNS
22937	Female	51	64.3	24.49	12.9	21	7.2239	2.1811	496.5	NDNS
23001	Male	60	75.4	24.34	14.1	61	17.148	1.1651	1566.8	NDNS
23003	Male	57	104.55	41.38	9.8	6	20.5179	1.1725	1764.1	NDNS
23009	Male	62	66.3	24.64	13.4	106	16.4647	0	1646.5	NDNS
23012	Male	47	94.05	30.5	16.4	157	12.9843	4.2296	699.4	NDNS
23014	Male	32	80.9	25.02	15.7	161	13.3765	3.7017	734.8	NDNS
23018	Female	54	100.55	43.26	13.4	61	13.1152	0.7435	959.4	NDNS
23027	Female	31	51.8	18.19	13.1	11	12.8935	0.5829	1210.1	NDNS
23028	Male	19	95.3	30.01	14.6	69	9.0551	2.565	803.9	NDNS
23109	Male	26	79.1	25.78	16.1	28	14.5234	3.1337	1135.1	NDNS
23130	Male	37	103.5	36.02	14.5	69	13.9385	2.1856	1555.6	NDNS
23131	Male	62	60.2	21.59	14.6	143	5.4087	1.0586	904.7	NDNS
23135	Female	61	109.75	43.01	14.7	98	7.2925	2.6619	435.1	NDNS
23137	Male	44	71.25	24.51	15.3	60	16.7594	0	1514.7	NDNS
23209	Male	30	107.4	28.6	14.6	108	47.6643	7.1844	1874.5	NDNS
23211	Female	20	68.8	27.82	13.1	11	9.2611	3.5215	721	NDNS
23219	Female	61	75.65	27.54	14.4	72	9.2061	1.6145	875.9	NDNS
23224	Male	53	95.15	35.99	16.1	129	15.1582	1.7133	1035.1	NDNS
23229	Female	38	67.2	28.43	13.2	95	7.9949	1.5956	581.1	NDNS
23236	Female	36	49.3	18.31	12.5	55	7.6741	1.2893	753.8	NDNS
23308	Female	52	90.7	35.19	13	66	10.924	2.373	712.5	NDNS
23312	Female	57	73	31.18	14	104	9.319	1.2807	626	NDNS
23325	Female	47	95.85	37.09	15	31	7.3204	1.4016	693.8	NDNS
23326	Male	54	105.9	34.78	15.6	480	7.9152	2.5955	949.8	NDNS
23401	Female	55	54.4	20.16	14.1	52	19.9156	1.2138	722.9	NDNS
23411	Female	35	59.25	20.83	12.1	45	13.0101	2.8671	1040	NDNS
23425	Male	57	78.05	25.13	15.7	148	12.4925	2.2063	807.3	NDNS
23504	Male	45	72.1	22.77	15.1	122	19.1733	0.4962	1044.4	NDNS
23510	Female	57	73.05	28.11	13.8	76	8.8871	0.8607	1093.9	NDNS
23511	Female	45	109.25	43.41	16.3	101	7.4411	1.2697	480.1	NDNS
23519	Male	56	105.3	30.31	15.2	169	20.2622	4.5626	1670.6	NDNS
23520	Female	33	70.05	28.97	14.1	32	3.0606	1.4727	592.5	NDNS

23527	Male	22	67	21.13	14.4	87	11.7329	2.3781	1086.7	NDNS
23610	Female	36	50.9	17.79	12.1	19	15.1021	0	750.1	NDNS
23620	Male	44	91.4	31.81	15.2	96	12.3839	2.1962	514.4	NDNS
23735	Male	44	115.85	34.03	17.2	166	15.3265	3.0561	1251.5	NDNS
23817	Male	62	71.95	25.21	15.4	145	10.1699	1.1832	751.9	NDNS
23819	Female	21	101.1	38.83	15.6	84	7.4937	2.1163	612.1	NDNS
23822	Male	36	83	27.73	15.1	97	9.0604	2.3369	658.6	NDNS
23912	Male	52	101.9	31.61	14.1	371	14.232	4.1763	887.6	NDNS
23915	Male	41	82.8	25.22	14.3	68	16.0278	1.9718	805.8	NDNS
23917	Female	43	56.9	22.62	13.5	63	11.6236	2.6666	825.2	NDNS
23918	Male	37	68.2	20.82	15.2	82	9.4368	4.4451	637.5	NDNS
23919	Male	51	98.7	34.91	15.8	197	18.6196	4.4544	1188.4	NDNS
23921	Female	37	71.9	25.66	14	53	15.1634	1.3596	1309.8	NDNS
23930	Male	54	98.9	31.51	13.8	23	10.8115	3.0198	999.4	NDNS
23931	Male	39	80.05	27.26	14.6	166	31.6093	3.9085	1498	NDNS
23935	Female	59	77.8	33.45	14.6	122	9.7864	2.3311	1120.7	NDNS
23936	Male	44	90.2	32.38	15.6	542	12.574	5.7626	729.8	NDNS
24002	Male	60	104	37.65	14.4	54	18.3391	7.5202	1286.8	NDNS
24003	Female	33	78.7	29.6	14.4	46	9.5747	1.7969	727.3	NDNS
24008	Male	46	78.35	27.55	15.4	104	5.0499	1.5012	494.6	NDNS
24009	Male	33	61.6	19.65	14.6	85	12.631	3.4458	1040.9	NDNS
24018	Female	20	46.3	18.72	12.7	8	10.5735	1.5229	646.2	NDNS
24019	Female	27	52.5	23.74	13.8	17	9.5626	0	676.2	NDNS
24025	Male	21	82.2	25.5	14.6	23	13.0061	3.092	1417.6	NDNS
24029	Female	39	54.7	19.65	13	6	11.2965	0.8691	861.1	NDNS
24033	Female	33	78.8	31.95	13.4	63	6.5267	1.6167	418.7	NDNS
24037	Female	34	70.9	25.71	14.2	23	11.1929	2.3274	1047.6	NDNS
24116	Male	63	73.2	21.98	14.8	31	20.2883	0.4075	1221.9	NDNS
24121	Female	43	57.8	21.52	13.4	16	7.9685	0.6095	565.2	NDNS
24135	Male	41	92.1	28.02	16.1	66	13.8571	3.0752	1200.5	NDNS
24136	Male	53	71.3	22.01	14.8	236	10.5321	2.4618	772.1	NDNS
24228	Male	41	81.6	29.14	16.6	61	7.4497	2.5528	880.8	NDNS
24305	Female	42	74.7	26.63	14.6	29	8.225	0.5221	1210.2	NDNS
24314	Female	25	57.5	21.38	14.4	21	7.7543	1.0878	631.5	NDNS
24428	Male	37	66.5	22.35	16.6	82	8.9301	1.5547	964.6	NDNS
24437	Female	30	75.1	28.83	12.5	23	10.9877	0.9621	884.5	NDNS
24439	Female	41	64	24.09		25	6.887	3.1342	436.9	NDNS
24505	Male	32	64.95	23.93	15.8	88	10.1365	4.1645	670.8	NDNS
24511	Male	56	65.5	25.36	14.9	140	13.5814	1.3352	1385.9	NDNS
24513	Male	46	95.5	31.4	13.9	19	10.3126	3.2282	845.4	NDNS
24514	Female	32	63.5	24.64	13.7	11	6.0032	0.5082	867.2	NDNS
24526	Male	64	101	32.18	14.7	52	11.7009	2.9221	1045.9	NDNS
24531	Male	58	69.75	22.95	15.7	182	16.677	6.2221	1353.3	NDNS
24539	Female	55	40	15.89	15.2	28	14.743	7.6362	579.8	NDNS
24603	Female	39	60.05	24.21	13.5	87	11.1496	3.1808	839.7	NDNS
24614	Male	62	74.3	24.36	15.2	253	19.9812	4.4588	1043.8	NDNS
24701	Female	61	58.1	25.7	11.2	49	9.3933	1.0918	747.1	NDNS
24703	Female	62	120.5	40.95	14.3	352	11.274	1.8249	1140.5	NDNS
24707	Female	29	57.1	25.16	13.5	34	8.2271	0.5382	931	NDNS
24711	Female	27	48.3	19.24	13	33	8.1127	2.2374	647.3	NDNS
24717	Female	54	72.85	29.86	14.9	238	14.5886	5.1699	641.8	NDNS
24724	Male	42	57.15	23.36	15.6	48	22.8665	4.2513	1134.6	NDNS
24727	Male	38	109.5	34.81	15.4	237	11.1821	2.5296	816	NDNS
24730	Female	62	59.5	22.35	13.3	21	17.1899	1.1289	1280.4	NDNS
24733	Female	57	70.9	29.74	14.2	155	11.6907	3.3516	752	NDNS
24735	Female	25	81.9	28.79	12.3	19	13.2169	1.8602	708.9	NDNS
24739	Male	45	62	20.99	12.8	9	7.5597	2.7201	528.4	NDNS
24809	Female	63	85.25	34.61	13.9	99	9.0456	2.5839	771.9	NDNS

24812	Male	59	77.55	27.74	15.1	74	16.4882	4.1725	1187	NDNS
24816	Male	46	75.25	28.94	15.2	211	5.1801	2.5447	686.6	NDNS
24820	Female	45	85.6	33.19	14	29	8.1755	1.283	1417.7	NDNS
24832	Male	64	94.4	31.16	14.6	268	10.3533	1.7086	692.4	NDNS
24834	Male	29	105.9	32.79	14.5	257	8.8585	0.7393	906.7	NDNS
24901	Male	58	93.8	28.29	16.3	253	16.2985	2.8114	896.5	NDNS
24911	Female	46	59.8	24.26	14.4	52	14.9273	2.0184	975.5	NDNS
24913	Female	37	76.95	28.58	13.4	34	8.6583	0.351	679.3	NDNS
24917	Male	37	87.9	27.08	15.9	84	15.1599	1.2907	1186.5	NDNS
24918	Female	40	51.5	20.85	13.7	27	10.5922	1.0823	952.8	NDNS
24928	Female	37	67.45	27.16	13.9	89	8.2298	2.169	482.2	NDNS
25002	Female	19	62.55	23.64	13.8	25	12.5976	1.2348	740	NDNS
25011	Male	51	83.85	30.87	14.6	319	9.1427	1.9592	1113	NDNS
25013	Male	61	71.4	27.19	15.1	180	14.1356	2.8875	1070.8	NDNS
25019	Male	31	76.05	24.41	15	86	10.1219	1.5205	1019.9	NDNS
25032	Male	45	93.95	30.87	15.4	258	20.1256	3.5831	1121.6	NDNS
25033	Female	39	52.3	19.52	10.2	12	7.125	1.5731	472.8	NDNS
25034	Female	58	78.75	29.9	12.7	109	10.5293	1.5812	732	NDNS
25101	Male	22	91.85	29.25	15.6	112	14.9185	5.3207	1147.8	NDNS
25107	Male	29	84.05	27.78	16.5	80	11.9728	2.4409	1802.3	NDNS
25121	Female	32	46.45	20.94	13.9	38	7.4954	0.5818	450.4	NDNS
25126	Female	25	68.2	22.96	12.5	21	7.518	0	704.8	NDNS
25129	Male	62	77.35	27.89	15.3	74	13.7706	0.4271	1210.6	NDNS
25205	Male	31	88.5	27.04	16.3	112	19.5795	1.1827	1498.2	NDNS
25225	Female	22	64.5	23	13.8	59	9.4337	1.1126	637.9	NDNS
29904	Male	35	62.65	22.45	15.7	159	12.7908	0.6102	1716.2	NDNS
29905	Female	34	114.1	42.19	13	73	20.1064	1.8929	1318.1	NDNS
29908	Male	41	92.6	31.41		258	22.1125	3.8071	994.3	NDNS
29909	Female	62	74.8	28.82	13.9	90	9.0177	0.4602	993.8	NDNS
29910	Male	23	66	21.42	15.8	58	11.0465	5.448	676.4	NDNS
29911	Female	31	46.5	17.43	13.9	42	6.3786	0.1828	383.4	NDNS
29914	Female	47	86.3	35.03	12.1	44	14.9084	3.0441	729.5	NDNS
29917	Male	45	76.95	26.21	15.1	51	7.003	0.8613	524.1	NDNS
29918	Male	28	109.05	30.67	15.8	68	8.9503	3.8266	692.1	NDNS
29920	Male	24	78.5	23.45	15.8	60	12.0457	1.5828	1257.6	NDNS
29921	Female	34	62.6	22.22	13	37	6.0788	1.2786	535	NDNS
29923	Male	57	75.6	25.41	16.3	99	14.6409	7.0419	836.8	NDNS
29926	Male	30	106.4	33.04	16.7	158	22.6252	3.6159	1602.9	NDNS
29928	Female	53	94	33.05	13.7	66	14.4777	0.6741	1060.8	NDNS
29932	Male	28	77.75	22.71	15.6	167	12.7776	1.1448	1041	NDNS
29935	Female	55	81.95	29.09	14.5	33	13.9118	1.2973	1038.1	NDNS
29937	Male	52	82.8	27.01	16.6	62	16.4227	1.1326	1133.9	NDNS
200001	Male	66	109	32.2	15.5	210	12.29	1.386429	774	NUAGE
200002	Female	65	61.8	22.4	14.2	332	13.05	1.672286	885	NUAGE
200003	Female	75	75.9	27.9	13.5	90	12.82	1.382857	923	NUAGE
200004	Male	74	88	27.5	13.3	166	18.83	2.576571	1567	NUAGE
200005	Male	66	84.5	26.4	14.9	276	12.47	0.777143	1204	NUAGE
200007	Female	69	62.3	23.7	12.8	65	9.71	1.091429	933	NUAGE
200012	Female	67	67.1	26.2	15.2	154	11.3	0.361429	1093	NUAGE
200013	Female	65	96	35.3	15.2	114	18.65	1.507429	1733	NUAGE
200014	Female	66	55.6	21.2	12.9	93	8.87	0.548571	948	NUAGE
200016	Male	70	76.1	22.7	15.9	114	19.74	1.051143	1184	NUAGE
200017	Female	72	88.7	34.6	13.4	85	11.2	0.721857	901	NUAGE
200018	Male	71	79.8	26.1	14.5	13	22.44	1.214714	553	NUAGE
200019	Female	69	63.1	24.3	13.5	83	15.94	0.302857	951	NUAGE
200020	Male	75	91.1	26.3	15.5	191	19.92	0.295714	1514	NUAGE
200021	Male	73	95.4	33.4	14.4	119	18.53	0.558	1071	NUAGE
200023	Female	65	81.7	31.5	13.5	43	14.46	1.098143	844	NUAGE

200025	Female	68	74.7	29.9	13.6	42	8.4	1.456143	548	NUAGE
200026	Female	68	67.1	25.3	14.4	59	9.83	0.236286	1011	NUAGE
200027	Female	67	85.2	30.9	13.8	81	11.96	0.204571	1156	NUAGE
200028	Female	74	65.5	25	13.3	81	8.48	1.115286	946	NUAGE
200029	Female	65	64.6	25.9	13.2	14	11.2	0.919714	604	NUAGE
200030	Female	68	65	30.1	13.1	151	11.58	3.110857	835	NUAGE
200033	Female	69	81.7	29.3	12.8	8	17.36	0.717	1189	NUAGE
200035	Male	67	78	22.5	15.9	350	15.14	2.405286	1269	NUAGE
200036	Male	69	62.6	23.6	15	67	8.51	0.502857	644	NUAGE
200037	Male	70	85.5	28.9	15	364	9.5	1.605286	1073	NUAGE
200040	Male	69	82.2	28.8	15	103	13.87	0.582	908	NUAGE
200042	Male	73	88.4	30.6	14.8	75	12.11	1.799143	1316	NUAGE
200043	Male	78	68	23	13.3	171	19.98	0.448	1491	NUAGE
200046	Male	65	64.1	18.9	14	123	12.24	0	1152	NUAGE
200047	Female	65	74.8	25.6	15.2	75	21.8	0	1441	NUAGE
200049	Male	73	73.9	24.7	15.2	165	17.46	0.337	1099	NUAGE
200051	Female	67	74	26.2	13	279	15.96	0.179429	1088	NUAGE
200052	Female	76	66.1	25.2	12.1	48	9.3	0.516286	823	NUAGE
200053	Female	66	59.3	25.3	15.2	55	8.77	0.241429	1055	NUAGE
200054	Female	67	71.1	26.4	13.8	50	12.24	1.108571	1119	NUAGE
200055	Male	66	87.2	28.8	14.7	170	12.11	1.083857	860	NUAGE
200056	Female	65	79.2	28.4	13.7	179	10.13	1.035857	943	NUAGE
200057	Female	68	55.5	24	12.8	117	10.67	0.584571	1606	NUAGE
200058	Male	65	94.8	30.3	13.9	80	17.5	1.202429	1623	NUAGE
200059	Female	70	71.2	26.8	14.4	103	14	0.185143	1266	NUAGE
200060	Female	74	99.4	37.4	13.6	81	10.04	1.492429	900	NUAGE
200063	Female	67	73.2	25.9	15.3	155	10.22	1.318857	960	NUAGE
200064	Female	70	69.2	25.7	13.2	57	13.13	0.942429	1177	NUAGE
200065	Male	76	96.4	30.4	14.5	78	21.28	0.787571	1678	NUAGE
200066	Female	71	87.5	29.9	14.4	219	9.32	2.666857	729	NUAGE
200067	Male	70	79.4	26.5	15.4	76	13.96	0.829857	1008	NUAGE
200069	Female	70	64.1	26.3	14.8	75	10.74	0.756714	1288	NUAGE
200071	Female	79	59.2	25.3	13.4	45	9.59	0.444286	831	NUAGE
200072	Female	73	65.9	23.9	13.4	109	11.21	1.495	968	NUAGE
200073	Female	68	51.4	22.8	11.8	52	8.51	0.221286	1195	NUAGE
200074	Female	78	60.1	21.8	13.2	28	11.34	0.061429	1369	NUAGE
200075	Male	70	87	27.8	13.8	103	12.82	1.904714	980	NUAGE
200077	Male	69	94.9	31	14.5	72	14.61	1.048571	878	NUAGE
200078	Female	65	80.1	30.9	14.1	58	11.4	1.086	856	NUAGE
200080	Male	66	87.8	27.1	15.3	249	17.64	2.502857	1257	NUAGE
200081	Female	65	64.3	27.1	13.3	68	8.93	1.49	852	NUAGE
200082	Female	67	66.5	26.3	14.2	265	10.18	1.200572	937	NUAGE
200083	Male	79	72.5	26.3	14.5	107	14.21	0.550857	1182	NUAGE
200084	Female	72	71.6	26.9	-9	23	10.41	0.809	1246	NUAGE
200085	Male	68	69.4	22.7	13.9	190	13.24	1.144	1302	NUAGE
200086	Female	68	68	24.7	-9	132	7	0.751	609	NUAGE
200087	Female	68	55.6	24.1	13.5	80	11.81	1.631857	1092	NUAGE
200088	Male	69	78.5	27.2	13.2	150	13.63	1.822143	1269	NUAGE
200090	Male	70	70.8	21.1	13.6	93	25.32	0.261714	1432	NUAGE
200091	Male	66	84.9	26.5	14.7	114	10.97	1.674	892	NUAGE
200092	Female	69	86.1	31.2	14.6	85	6.45	0.293571	888	NUAGE
200093	Male	73	87.8	29	16.7	303	19.86	1.418429	1318	NUAGE
200097	Female	67	59.4	23.2	13.6	142	12.55	0.462143	920	NUAGE
200099	Female	74	73.2	29	13.1	83	8.43	0.973286	705	NUAGE
200101	Male	71	79.9	22.6	14.6	72	13.11	1.291714	878	NUAGE
200103	Male	72	68.9	24.4	15.2	125	10.87	1.414057	873	NUAGE
200104	Male	76	93.9	32.5	15.3	321	9.99	1.286	948	NUAGE
200105	Female	65	67	25.5	11.5	15	13.91	0.221143	1361	NUAGE

200106	Female	67	87.9	25.1	12.4	20	12.45	0.201714	1412	NUAGE
200107	Female	67	61.7	23.8	13.8	133	13.85	3.133572	827	NUAGE
200108	Female	66	67.6	26.7	-9	80	20.24	0.813571	908	NUAGE
200109	Female	71	58.9	24.5	13.7	52	13.53	0.382857	768	NUAGE
200110	Male	68	68.8	23.3	15.4	84	15.66	0.639286	918	NUAGE
200111	Male	69	77.1	27.6	16.4	108	10.25	2.696143	1052	NUAGE
200112	Female	77	56.5	22.6	12.5	103	14.52	0.447571	917	NUAGE
200116	Male	65	63.5	21.5	14.9	70	12.68	1.132429	749	NUAGE
200117	Female	69	86.2	28.5	11.3	11	10.76	0.740286	1010	NUAGE
200118	Male	67	93.8	34.9	15.5	232	12.52	1.394714	874	NUAGE
200120	Female	67	62.3	26.3	13.8	130	11.46	0.647857	651	NUAGE
200121	Male	69	108.9	34.4	14.4	122	12.05	0.859572	912	NUAGE
200124	Male	79	92.7	29.6	15.5	72	12.8	0.974143	793	NUAGE
200127	Female	65	57.7	25	12.5	177	14.69	0.613857	1663	NUAGE
200128	Female	65	78.2	28.7	12.9	90	10.77	0.488	939	NUAGE
200129	Male	71	68.2	22.8	14	243	16.99	0.808	1309	NUAGE
200134	Male	69	83.5	24.9	12.7	66	11.66	0.203429	1470	NUAGE
200135	Female	68	65.8	25.7	15.1	277	17.37	1.905714	1155	NUAGE
200136	Male	77	84.2	30.9	14.9	127	12.98	1.478286	1039	NUAGE
200138	Female	71	57	20.4	13.2	114	8.48	0.127714	744	NUAGE
200140	Male	65	72.9	24.6	14	13	16.98	1.530743	1560	NUAGE
200142	Female	77	72.2	29.3	13.4	113	11.56	2.041657	655	NUAGE
200146	Female	67	53.8	19.8	14.1	87	10.17	0	989	NUAGE
200147	Female	78	66	26.4	13.7	25	6.98	0.390143	929	NUAGE
200148	Male	73	99.5	32.9	15.3	486	13.61	3.025	1225	NUAGE
200149	Female	65	102.5	43.2	13.4	96	7.65	1.620857	493	NUAGE
200150	Female	69	49.5	19.8	13.4	71	13.39	1.456	1010	NUAGE
200151	Male	68	78.3	24.4	12.7	123	10.08	1.235714	921	NUAGE
200152	Female	71	85.7	33.9	13.9	154	9.48	0.881857	1278	NUAGE
200153	Female	76	70.2	28.5	13.8	90	5.99	1.108286	1037	NUAGE
200154	Male	72	86.6	29.3	15.6	118	14.52	0.735286	1362	NUAGE
200155	Female	71	70.3	29.6	12.8	33	10.35	1.189857	1084	NUAGE
200156	Female	70	56.2	25.3	12.6	45	7.75	0.461143	677	NUAGE
200157	Female	72	69.3	27.4	13.8	109	10.86	2.421714	821	NUAGE
200158	Male	73	72.3	24.7	13.8	67	17.97	2.664572	1654	NUAGE
200159	Female	75	59.5	23	13.5	111	10.14	1.898429	1135	NUAGE
200162	Male	67	79.9	28.3	15.1	158	13.84	1.340429	1049	NUAGE
200163	Female	71	54.1	22.5	13.9	167	15.41	1.619	788	NUAGE
200164	Male	73	73.8	24.1	13.9	90	13.49	0.948857	1357	NUAGE
200166	Male	67	71.2	22.7	15.2	80	13.17	0.756143	1399	NUAGE
200168	Female	70	82.1	28.7	14	152	8.18	1.471571	791	NUAGE
200169	Male	66	72.8	24.6	15.4	138	16.16	3.077714	1013	NUAGE
200170	Female	78	67.2	25.3	13.5	70	13.05	0	1392	NUAGE
200173	Female	72	63.1	22.9	12.4	82	10.13	0.683286	887	NUAGE
200176	Male	67	85.3	30.6	16.5	85	15.9	0.286857	1649	NUAGE
200177	Female	71	48.6	21.3	13.1	46	17.29	1.180571	825	NUAGE
200178	Female	66	63.5	23.6	12.7	84	9.38	0.912143	1033	NUAGE
200179	Male	71	77.8	24.3	14.1	332	16.42	1.389428	1394	NUAGE
200180	Female	67	53.8	20	13.4	51	12.09	1.357429	898	NUAGE
200181	Female	69	65	29.7	12.7	85	8.82	0	840	NUAGE
200182	Female	69	65.4	26.9	15	236	11.42	2.984429	876	NUAGE
200183	Male	71	72.5	26	14.6	264	12.92	3.064286	900	NUAGE
200184	Female	71	66.9	23.4	13.6	166	13.92	0.385571	1644	NUAGE
200185	Female	69	79.2	26.8	11.8	280	8.44	1.134143	1181	NUAGE
200187	Male	70	69.5	24.6	13.9	26	11.18	1.697143	964	NUAGE
200189	Female	72	73.1	25.9	14	96	8.81	0.32	869	NUAGE
200190	Female	78	62.4	24.1	12.7	66	11.04	0.114286	1286	NUAGE
200191	Male	76	70.6	25	13.8	129	13.56	0.9558	1370	NUAGE

200192	Female	68	67.2	25	13	100	10.75	0.912657	558	NUAGE
200193	Female	73	77.4	29.9	13.4	230	12.14	1.244286	1034	NUAGE
200194	Male	72	91.4	28.5	14.2	118	18.21	1.565571	1112	NUAGE
200197	Male	66	96	31	14	74	21.34	2.069286	1485	NUAGE
200200	Female	69	59.3	25	13.7	107	8.16	1.197572	674	NUAGE
200201	Female	67	83.8	29	12.2	297	12.6	1.045143	841	NUAGE
200202	Female	70	80.5	27.5	13.9	188	11.36	0.929429	1228	NUAGE
200204	Female	69	50.7	21.1	12.6	95	22.98	0.642143	1117	NUAGE
200205	Female	67	47.6	20.3	13	66	10.64	0.985714	1031	NUAGE
200206	Male	66	88.6	28	15.8	127	12.69	0.621286	1622	NUAGE
200207	Female	70	68.3	24.2	14.9	159	13.74	0.965571	1017	NUAGE
200208	Female	71	77.3	26.7	13.6	69	9.7	0.504857	1049	NUAGE
200209	Male	79	108.9	32.9	14.4	205	14.81	0.839143	1271	NUAGE
200211	Female	75	77.2	29.8	14.1	112	11.03	1.310429	761	NUAGE
200212	Female	69	63.9	25.9	13.3	55	10.52	0.764429	1309	NUAGE
200214	Male	68	83.3	24.1	13.9	20	17.06	2.147572	1288	NUAGE
200215	Female	67	52.6	22.5	13.1	76	15.4	1.600143	1167	NUAGE
200216	Female	66	68.5	25.5	14	141	17.41	1.192143	1212	NUAGE
200217	Female	69	66	22.8	14.2	62	14.98	1.694429	1010	NUAGE
200219	Female	68	57.8	23.4	12.8	56	11.86	0.336429	1035	NUAGE
200220	Male	70	89.4	30.2	14	117	10.71	0.459286	860	NUAGE
200224	Female	77	62	25.5	15.6	88	14.77	1.229857	1033	NUAGE
200225	Male	72	78.9	27	14.5	507	10.14	1.098857	525	NUAGE
200226	Male	76	74.3	24	13.1	42	13.48	2.884286	950	NUAGE
200228	Female	76	73.7	29.2	12.8	53	8.28	0.565714	1036	NUAGE
200229	Female	76	65.3	25.8	14.5	112	15.49	1.093	1015	NUAGE
200231	Female	70	66.8	27.4	12.9	16	10.9	1.040143	735	NUAGE
200232	Male	69	109.1	28.4	15.6	240	11.11	1.428571	836	NUAGE
200233	Female	66	67.4	27.3	14	137	8.91	0.808857	607	NUAGE
200234	Male	65	63.8	20.6	13.6	56	13.32	2.188143	770	NUAGE
200237	Female	69	61.3	22.5	12.4	89	14.36	1.592	1119	NUAGE
200238	Male	65	58.7	18.5	15	27	12.35	0.698571	1087	NUAGE
200239	Female	66	58	24.1	14.6	24	13.44	0.931	1235	NUAGE
200240	Female	77	62.4	25	13.7	87	8.79	0.139857	1077	NUAGE
200241	Male	72	71.4	24.7	15.1	61	11.7	0.303714	1591	NUAGE
200243	Male	65	101.7	34	15.3	187	15.69	0.967143	468	NUAGE
200247	Female	66	78.6	26.3	15	111	11.23	1.434857	983	NUAGE
200248	Female	76	74.7	31.5	13	172	12.15	0.157143	1051	NUAGE
200250	Male	74	96.8	31.3	13.8	30	7.56	0.08	1104	NUAGE
200251	Male	69	81.3	27.5	14.8	39	12	3.446857	758	NUAGE
200252	Male	78	88.4	31.3	14.8	205	13.51	0.872714	1126	NUAGE
200253	Male	71	66.6	23.6	14.8	302	14.26	3.812143	1153	NUAGE
200254	Male	70	74.4	25.7	14.1	106	12.6	0.945714	1506	NUAGE
200256	Male	67	94.2	27.5	14.5	72	14.79	0.588571	1202	NUAGE
200259	Female	77	67.2	27.3	12.5	95	8.42	0.580143	666	NUAGE
200260	Male	68	89	27.8	16.5	123	11.72	1.247	1012	NUAGE
200261	Female	72	84.7	31.5	13.5	131	7.15	0.138286	455	NUAGE
200262	Male	67	69.6	26.9	15	261	13.18	0.753572	1502	NUAGE
200263	Female	66	70.9	26	14.5	124	9.36	0.42	1215	NUAGE
200264	Female	69	70.1	28.4	13.7	244	10.74	0.861429	881	NUAGE
200267	Male	72	108.1	33.7	14	194	13.93	0.543572	1347	NUAGE
200268	Female	78	84.8	33.5	13.9	165	9.92	0.868857	977	NUAGE
200269	Female	66	65	30.1	12.7	87	9.38	0.571429	734	NUAGE
200271	Female	78	79.7	28.6	12.8	197	13.62	1.470714	991	NUAGE
200272	Female	72	63	24.9	13	280	10.88	0.853857	814	NUAGE
200275	Female	65	85.8	30.4	13.5	93	12.34	1.219857	969	NUAGE
200276	Female	68	61.3	24.6	14.1	97	11.77	0.666	779	NUAGE
200277	Female	67	62.9	20.8	12.5	127	8.85	1.13	957	NUAGE

200280	Female	67	70.1	26.1	12.7	26	12.68	2.427286	1011	NUAGE
200281	Female	71	78.9	28.3	13.4	33	16.96	2.819429	1247	NUAGE
200283	Male	68	79.2	28.4	14.1	57	11.11	0.644286	1240	NUAGE
200284	Female	66	51.2	21	12.7	37	9.78	0.296571	991	NUAGE
200285	Female	69	69.4	24.9	11.7	58	13	0.846286	1084	NUAGE
200287	Female	67	69.6	24.7	14	47	13.63	0	987	NUAGE
